# The PLK4 inhibitor RP-1664 demonstrates potent efficacy in neuroblastoma preclinical models through a dual mechanism of sensitivity

**DOI:** 10.1038/s41467-026-74061-5

**Published:** 2026-06-13

**Authors:** Isabel Soria-Bretones, Matias Casás-Selves, Minu Samanta, David Groff, Jayne Murray, Jamie I. Fletcher, Alvin Farrel, Steven Pastor, Khushbu Patel, Elliot Goodfellow, Li Li, Cathy Caron, Ariya Shiwram, Hyeyeon Kim, Danielle Henry, Nancy Laterreur, Julian Bowlan, Kateryna Krytska, Steven B. Neuhauser, Timothy M. Stearns, Jeffrey A. Schubert, Jinhua Wu, Lea F. Surrey, Daniel Martinez, Crystal Mak, Jennifer Brand, Caitlin Wesley, Klaartje Somers, Alejandro Álvarez-Quilón, Frédéric Vallée, Parham Nejad, Joseph D. Schonhoft, Joanna Li, Artur Veloso, Jordan T. F. Young, Marc L. Hyer, Stephen J. Morris, Yael P. Mossé, C. Gary Marshall, Michelle Haber, Michal Zimmermann, John M. Maris

**Affiliations:** 1 Repare Therapeutics, St. Laurent, QC Canada; 2 Divison of Oncology and Center for Childhood Cancer Research, Children’s Hospital of Philadelphia, Philadelphia, PA USA; 3 Children’s Cancer Institute at Minderoo Children’s Comprehensive Cancer Centre, Sydney, NSW Australia; 4 School of Clinical Medicine, UNSW Sydney, Kensington, NSW Australia; 5 Repare Therapeutics, Cambridge, MA USA; 6 The Jackson Laboratory, Bar Harbor, ME USA; 7 Department of Pathology and Laboratory Medicine, Children’s Hospital of Philadelphia, Philadelphia, PA USA; 8 Department of Pediatrics, Perelman School of Medicine at the University of Pennsylvania, Philadelphia, PA USA

**Keywords:** Paediatric cancer, Target validation, Cell division

## Abstract

It was recently shown that inhibition of polo-like kinase 4 (PLK4) induces synthetic lethality in cancers with chromosome 17q-encoded *TRIM37* copy number gain due to cooperative regulation of centriole duplication and mitotic spindle nucleation. We show here that chromosome 17q/TRIM37 gain is a defining feature of high-risk neuroblastoma and renders patient-derived cell lines hypersensitive to the novel PLK4 inhibitor RP-1664. We demonstrate that centriole amplification at low doses of RP-1664 contributes to this sensitivity in a *TRIM37*-independent fashion. CRISPR screens and live cell imaging reveal that upon centriole amplification, neuroblastoma cells succumb to multipolar mitoses due to an inability to cluster or inactivate supernumerary centrosomes. RP-1664 monotherapy showed robust anti-tumor activity in 14/15 human neuroblastoma-derived xenograft models, and significantly extended survival in a transgenic *MYCN*-driven murine model of neuroblastoma. RP-1664 combined with GD2-directed chemoimmunotherapy resulted in maintained complete responses in 6/9 mice with established *MYCN*-driven murine neuroblastomas. These data support clinical development of PLK4 inhibitors for high-risk neuroblastoma and other cancers with somatically acquired *TRIM37* overexpression.

## Abstract (web summary)

Inhibition of PLK4 is synthetic lethal in cancers with chromosome 17q TRIM37 copy number gain. Here, the authors show that while RP-1664 (PLK4 inhibitor) causes centrosome depletion in a TRIM37-dependent manner as high doses, low dose causes cell death in a TRIM37-independent manner via centrosome amplification and demonstrate its efficacy in preclinical models of neuroblastoma with TRIM37 gain.

## Introduction

High-risk neuroblastoma is a childhood cancer with a 5-year survival rate of approximately 50% despite dose-intensive chemotherapy including autologous stem cell transplantation, surgery, radiation therapy, and immunotherapy^1^. Genomic gain of the long arm of chromosome 17 (17q) by unbalanced translocation is among the most recurrent molecular alterations in neuroblastoma^2–6^, but the exact prevalence of this genomic aberration as well as its biologic and clinical relevance remains poorly defined. Chromosome 17q gain often spans a region between 17q11 and 17q25, which contains the *TRIM37* gene located at 17q22-23^2,7,8^. Recently, *TRIM37* genomic gain and high expression was shown to confer a cancer therapeutic vulnerability due to synthetic lethality with inhibition of polo-like kinase 4 (PLK4)^9,10^.

PLK4 is a dimeric serine-threonine kinase that plays key roles in centrosome formation^11^. The centrosome, the main microtubule-organizing center of the cell, is composed of two centrioles and the peri-centriolar material (PCM), which together nucleate microtubules that form the mitotic spindle^11,12^. The formation of centrosomes is tightly regulated. Each mitotic cell normally has two centrosomes, which drive bipolar division and equal separation of genetic material (as well as centrosomes) into two daughter cells. In turn, each G1 daughter cell contains one centrosome with one centriole doublet that is duplicated in S-phase, ensuring that the subsequent mitosis will again contain two centrosomes^12^. PLK4 is critical for centriole biogenesis: it interacts with, and phosphorylates, several centriolar and PCM proteins to drive duplication of the parent centriole^13–17^. Consequently, PLK4 downregulation leads to progressive loss of centrioles, whereas PLK4 overexpression leads to centriole amplification and supernumerary centrosomes^13,14,18^. To guarantee that centriole duplication occurs only once per cell cycle, PLK4 also regulates its own stability: trans-autophosphorylation of several serine and threonine residues within a PLK4 dimer activates degradation of the protein by the SCF-βTrCP ubiquitin ligase^18–21^. Catalytically inactive PLK4 therefore accumulates in the cell^18–21^.

Synthetic lethality between high TRIM37 levels and PLK4 inhibition is based on their complementary roles in centrosome biogenesis^9,10^. PLK4 inhibition causes centriole loss, for which normal cells compensate in part by using the PCM to nucleate the mitotic spindle^9,10^. However, as *TRIM37* encodes an E3-ubiquitin ligase that negatively regulates the stability of multiple PCM components through proteasomal degradation^9,10,22^, *TRIM37* overexpression compromises the PCM’s integrity. PLK4 inhibition in the context of high TRIM37 abundance thus leads to depletion of both centrioles and the PCM, an inability to form the mitotic spindle, and mitotic failure or delay^9,10^. Prolonged mitosis upon PLK4 inactivation is also sensed by the mitotic surveillance (or ‘stopwatch’) pathway driven by the USP28-53BP1-p53 axis, which triggers a p21-mediated cell cycle arrest and cell death^9,10,23–26^. Based on these synthetic lethality paradigms, we developed RP-1664: a selective, potent, and orally bioavailable PLK4 inhibitor^27^. RP-1664 is highly selective for PLK4: it does not engage the majority of ~300 kinases in mammalian cells and shows over 2000-fold selectivity against the structurally related Aurora A and B kinases^27^. RP-1664 also displays favorable ADME properties and oral bioavailability in multiple pre-clinical mammalian species^27^, and is presently being evaluated in a first-in-human Phase 1 clinical trial for patients with refractory solid malignancies showing gain of the *TRIM37* locus (NCT06232408).

The effect of pharmacologic PLK4 inhibition on centriole biogenesis is bimodal. Whereas complete PLK4 inactivation at higher concentrations of a PLK4 inhibitor leads to centriole loss, lower concentrations induce centriole amplification^28–31^. This centriole amplification has been attributed to an intermediate inhibition state of PLK4 with enough inactive PLK4 monomers existing to reduce trans-autophosphorylation and increase total PLK4 levels, but not enough PLK4 molecules are inhibited to suppress centriole duplication^28,32,33^.

Here we show that centriole overduplication driven by low concentrations of RP-1664 contribute to PLK4i-sensitivity of neuroblastoma tumor cells, independent of *TRIM37* and *TP53*. This is because at low doses of RP-1664, neuroblastoma cells are unable to compensate for supernumerary centrosomes, whereas at higher doses there is the anticipated centriolar loss and mitotic delay. We then show that 17q gain including the *TRIM37* locus is a near universal feature of high-risk neuroblastoma and that RP-1664 shows robust single agent efficacy across a panel of human neuroblastoma cell line derived xenograft (CDX) and patient-derived xenograft (PDX) models, as well as a genetically engineered mouse model (GEMM) of this disease including potenty synergy with GD2-directed chemoimmunotherapy, supporting clinical development of PLK4 inhibition strategies for high-risk neuroblastoma.

## Results

### RP-1664 is a PLK4 inhibitor with pre-clinical activity against *TRIM37*-amplified and *TP53* wild-type tumors

**Fig. 1:**
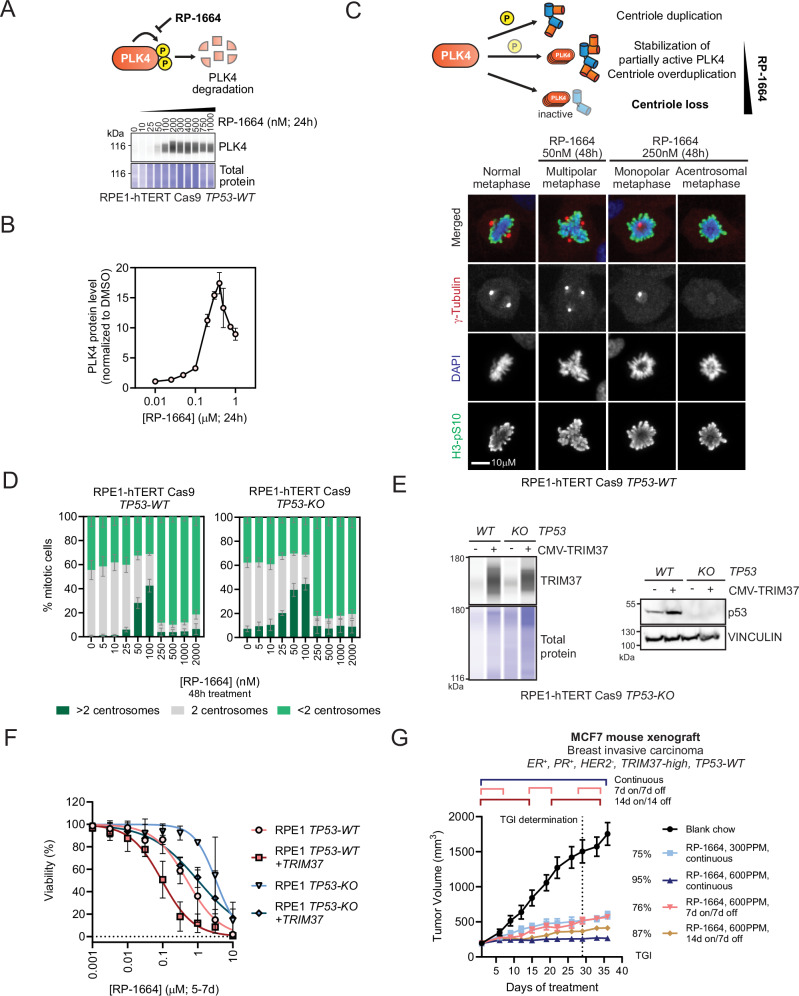
RP-1664 is a potent and selective PLK4i. **A**, **B** RP-1664 induces PLK4 stabilization. A. *Top:* Model of PLK4 self-regulation. PLK4 autophosphorylation leads to its degradation. This is blocked by RP-1664. *Bottom:* Representative capillary immunodetection of PLK4 in RPE1-hTERT Cas9 *TP53-KO* whole cell extracts. Total protein shown as a loading control. B. Representative (of *N* = 3 independent experiments) quantification of PLK4 protein levels in capillary immunodetection assays. Mean of two technical replicates from one representative experiment ±SD. **C**, **D** RP-1664 modulates centrosome biogenesis. C. *Top:* Schematic of bimodal modulation of centriole numbers by RP-1664. Low concentrations induce centriole amplification, higher concentrations lead to centriole loss. *Bottom:* Representative micrographs of RPE1-hTERT Cas9 *TP53-KO* cells after no treatment or treatment with indicated RP-1664 concentrations and immunofluorescence staining with γ-Tubulin (visualizing centrosomes) and H3-pS10 (mitotic marker) antibodies. DAPI is a nuclear counterstain. **D** Quantification of mitotic RPE1 Cas9 *TP53-WT* and *KO* cells with <2, 2, and >2 centrosomes at indicated RP-1664 concentrations in *N* = 3 independent experiments. Mean value (bars) is shown ±SD. **E**–**G** WT p53 and high TRIM37 sensitize to RP-1664. **E** Representative (of *N* = 2 independent experiments) TRIM37 capillary immunodetection (left) and TP53 immunoblot (right) of RPE1-hTERT Cas9 *TP53-WT* and KO cells with or without CMV-TRIM37 overexpression. Total protein and vinculin are loading controls. **F** Dose-response of RP-1664 on growth (measured by Incucyte) of RPE1 *TP53-WT* and *KO* cells, with or without CMV-TRIM37. Mean of *N* = 3 independent experiments ±SD. Solid lines show a non-linear regression fit to a four-parameter dose-response model. **G** Tumor volume measurements of MCF7 mouse xenograft tumors in animals fed blank chow or RP-1664-containing chow at indicated doses and schedules. TGI = percent tumor growth inhibition relative to blank chow. Mean of *N* = 6 mice/group ±SEM. Source data: SourceData_Figure1.xlsx and SourceData_uncropped_blots.pdf.

RP-1664 is a highly selective and orally bioavailable PLK4 inhibitor^27^. To confirm that RP-1664 inhibits PLK4 in human cells, we assessed its ability to induce PLK4 stabilization, modulate centrosome numbers, and activate the *TP53* mitotic surveillance pathway (Fig. 1A–D; and Supplementary Fig. 1A, B). First, we analyzed the effect of RP-1664 on PLK4 protein level, as inhibition of PLK4 counteracts its SCF-βTrCP-mediated degradation^18–21^. RP-1664 treatment led to a dose-dependent increase in total PLK4 in RPE1-hTERT Cas9 *TP53-*wild type (WT) cells when measured by capillary-based immunodetection, with a maximal effect observed above 100 nM and partial PLK4 stabilization occurring below this concentration (Fig. 1A, B). To determine whether RP-1664 disrupts PLK4-dependent centriole biogenesis, we quantified the number of centrosomes (visualized by anti-γ-Tubulin immunofluorescence^34^) per mitosis (marked by phosphorylated serine 10 on histone H3 – H3pS10^35^) using high-content fluorescence microscopy (Fig. 1C, D). In both *ΤP53*-proficient and -deficient RPE1 cells, RP-1664 induced centrosome loss at and above 250 nM, with most mitotic cells showing one or no centrosomes after approximately two population doublings (Fig. 1C, D). Concentrations between 25–100 nM induced supernumerary centrosomes, as expected from partial PLK4 inhibition^28^ (Fig. 1C, D). Finally, to test if RP-1664 activates the mitotic surveillance pathway, we stained *TP53*-WT RPE1 cells for the p53 transcriptional target CDKN1A/p21 (Supplementary Fig. 1A). RP-1664 increased p21 expression in a dose-dependent manner, at concentrations in agreement with those inducing PLK4 stabilization and modulating centrosome numbers (Supplementary Fig. 1A, B). Together, these data show that RP-1664 induces cellular phenotypes expected from a PLK4i^28,29,31,36^.

To evaluate the contributions of *TRIM37* and *TP53* to RP-1664 sensitivity^9,10^, we overexpressed the TRIM37 open reading frame in RPE1-hTERT Cas9 *TP53-WT* and *TP53-KO* cells. Lentiviral TRIM37 transduction led to a 3-5x increase in TRIM37 protein level (Fig. 1E; and Supplementary Fig. 1C). We then compared RP-1664 sensitivity of TRIM37-overexpressing cells with parental *TP53-WT* and *TP53-KO* cells by real-time monitored growth assays (Incucyte), revealing an ~30-fold increase in sensitivity from TRIM37-normal/*TP53-KO* cells to TRIM37-high/*TP53-WT* cells, with TRIM37-high/*TP53-KO* and TRIM37-normal/*TP53-WT* cells showing intermediate sensitivity (Fig. 1F). Concentrations of RP-1664 that completely inhibited growth of TRIM37-high/*TP53-WT* cells were consistent with the doses of RP-1664 that induce centrosome loss (at and above 250 nM; Fig. 1D). These data confirm that high TRIM37 level and functional p53 cooperatively increase cellular sensitivity to PLK4 inhibition by RP-1664.

To determine the activity of RP-1664 against *TRIM37-high TP53-WT* tumors in vivo we engrafted MCF7 breast carcinoma cells subcutaneously into immunodeficient mice. MCF7 cells express estrogen and progesterone receptors but no human epithelial growth factor receptor (ER^+^, PR^+^, HER2^-^), carry a *TRIM37*-gain, are *TP53-WT*, and have been previously shown to be sensitive to PLK4i in a *TRIM37*-dependent manner^9^. For successful implantation, mice were irradiated and supplemented with estradiol in their drinking water. Mice with implanted tumors were treated with RP-1664 delivered in chow at multiple doses and schedules (Fig. 1G), leading to dose-dependent anti-tumor activity of RP-1664 with maximal tumor growth inhibition (TGI) of 95% at 600 parts per million (ppm) RP-1664 chow (Fig. 1G). RP-1664 demonstrated schedule flexibility, with an intermittent 14 days on/7days off schedule showing only modest reduction in efficacy compared to continuous delivery (Fig. 1G). All doses and schedules were well tolerated with only one animal showing body weight (BW) loss of >20% (22% on the last day of dosing) in the continuous 600 ppm cohort, despite estradiol supplementation negatively impacting body weight loss in all groups (Supplementary Fig. 1D). We conclude that RP-1664 shows efficacy in a pre-clinical tumor model with *TRIM37* gain and WT *TP53*.

#### Chromosome 17q gain is a pathognomonic biomarker of high-risk neuroblastoma

**Fig. 2:**
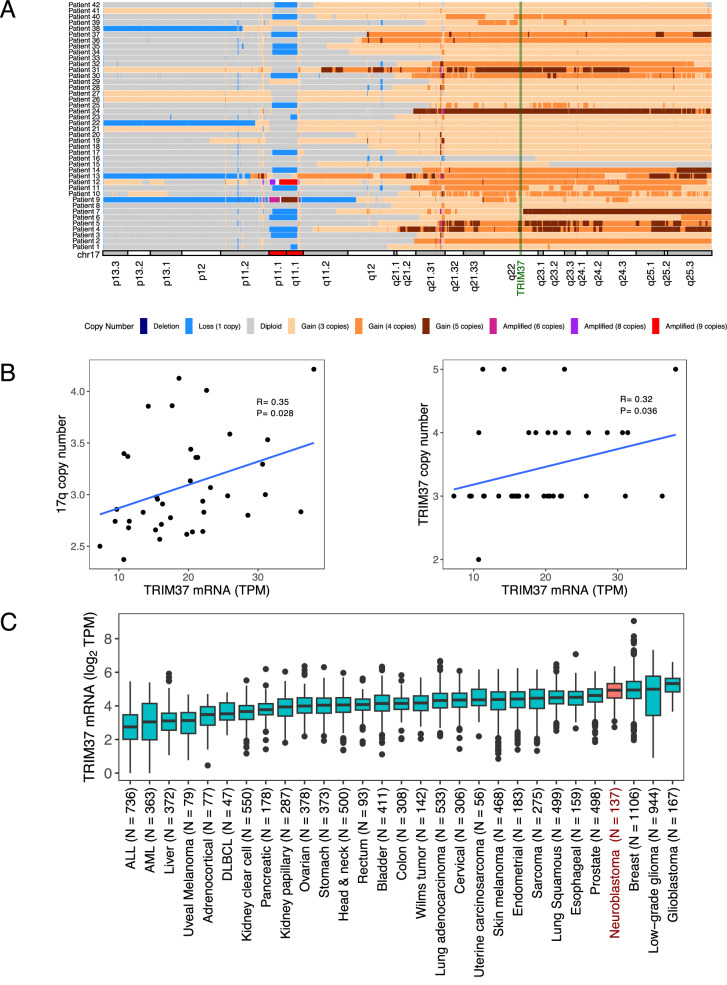
*TRIM37* gain is a pathognomonic feature of high-risk neuroblastoma. **A** Copy number variation across chromosome 17 in *N* = 42 high-risk neuroblastomas from the Gabriella Miller Kids First (GMKF) patient cohort. Colors indicate copy number as described below the plot. Cytogenetic map of chromosome 17 is shown for reference. **B** Correlation between *TRIM37* mRNA expression and 17q (left) or *TRIM37* (right) copy number in the GMFK neuroblastoma dataset as in A. *P* values were calculated using a two-tailed unpaired T-test based on the Pearson’s correlation coefficient (*R*) and the sample size (*N*) where the *T*-statistic = $R\cdot \sqrt{(N-2)/(1-{R}^{2})}$. **C**. *TRIM37* mRNA expression across tumor indications in the OpenPedCan dataset. Source Data (copy number and mRNA expression) can be retrieved from the OpenPedCan project (https://github.com/d3b-center/OpenPedCan-analysis).

The reported high prevalence of 17q gain in neuroblastoma presents a compelling rationale for development of a PLK4i in this disease, but estimates of the exact frequency of this aberration vary widely in the literature^2–6^, likely due to evolving genomic analytic technologies. To define the frequency of *TRIM37* copy number gain in high-risk neuroblastoma, we analyzed whole genome sequencing data from the Gabriella Miller Kids First (GMFK) cohort of primary diagnostic neuroblastomas (dbGaP Study Accession: phs001436.v1.p1). After extensive quality control to remove cases with low tumor content and/or low sequencing coverage (see “Methods”), all 42 high-risk neuroblastomas showed evidence for 17q gain (37 with segmental 17q gain, 5 with whole chromosome 17 gain), with all but one sample showing gain at the *TRIM37* locus (Fig. 2A). To further validate this finding, we reviewed 45 consecutive newly diagnosed high-risk neuroblastoma samples submitted for NGS using the CHOP solid tumor gene panel^37^. All 45 cases showed 17q gain including the TRIM37 locus by inference from surrounding genes on the NGS panel (39 with segmental 17q gain, 6 with whole chromosome 17 gain). Finally, *TRIM37* copy number and mRNA levels were directly associated and neuroblastoma showed nearly the highest median mRNA expression level of all human cancers (Fig. 2B, C).

#### *TRIM37/TP53*-dependent and -independent PLK4i sensitivity in neuroblastoma cells

**Fig. 3:**
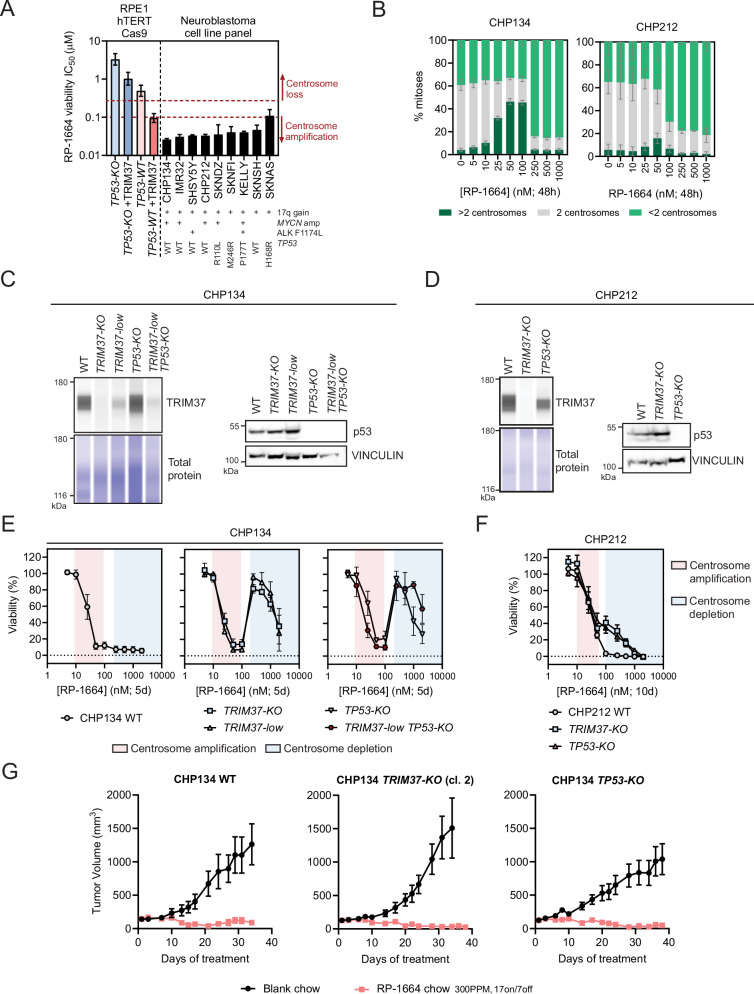
TRIM37- and p53-independent sensitivity of neuroblastoma cells to centrosome amplification. **A** RP-1664 cell growth IC_50_ values for indicated cell lines. Data from non-linear least square fitting of mean viability values (*N* = 3 independent experiments) in growth (Incucyte (RPE1, CHP134) or CellTiter Glo (all others)) assays ±95% confidence interval. Dashed line show concentrations inducing centrosome amplification and loss, respectively, in RPE1 cells. **B** Quantification of mitotic CHP134 and CHP212 cells with <2, 2, and >2 centrosomes at indicated RP-1664 concentrations in *N* = 3 independent experiments. Mean value (bars) is shown ±SD. **C**, **D** Representative TRIM37 capillary immunodetection (left) and p53 immunoblots (right) of CHP134 (**C**) and CHP212 (**D**) of indicated genotypes. Total protein and vinculin are loading controls. **E**, **F** Cell viability of CHP134 (**E**; measured by Incucyte) and CHP212 (**F**; CellTiter Glo) cells of indicated genotypes treated with indicated RP-1664 concentrations. Pink area shows concentrations causing centrosome amplification, blue represents centrosome depletion. Mean of *N* = 3 (**F**) and 5 (**E**) independent experiments ±SD. **G** Tumor volume measurement of CHP134 WT, *TRIM37-KO* and *TP53-KO* mouse xenografts treated with blank chow or 300 ppm RP-1664 chow using a 17 d on / 7 d off schedule. Mean of 7 mice/group ±SEM. Source data: SourceData_Figure3.xlsx and SourceData_uncropped_blots.pdf.

Due to near universal prevalence of *TRIM37* gain in neuroblastoma, we sought to define the potency of RP-1664 in preclinical models of this disease. We observed high sensitivity to RP-1664 across a panel of nine neuroblastoma cell line models carrying *TRIM37* gain, with a median viability IC_50_ of 35 nM (range 26-109 nM; Fig. 3A). *TP53* mutated cell lines tended to show a higher IC_50_, but the trend was subtle (median for *TP53* WT vs. *TP53* mutant: 33 and 40 nM, respectively; *P* = 0.11, Mann-Whitney test). Neuroblastoma cell lines also showed overall higher RP-1664 sensitivity when compared to a panel of nine breast cancer cell lines that each carry at least one extra copy of *TRIM37* (Supplementary Fig. 2A, Supplementary Table 1), as well as RPE1 cells and four additional non-transformed human cell lines derived from various tissues (MCF10A – originating from a fibrocystic mammary epithelium, COL-hTERT – an hTERT-immortalized colon cell line, BRONCH2 – SV40-immortalized bronchial epithelial cells, and HK2 – E6/E7-immortalized kidney epithelium; Supplementary Fig. 2A).

Interestingly, the median IC_50_ across all neuroblastoma cell lines was below the 250 nM concentration that is required for depletion of centrosomes in RPE1 cells and instead correlated with centrosome amplification (Fig. 3A, B). This was not due to cell type-dependent differences in modulation of centrosome number by RP-1664, as we observed centrosome amplification between 10 to ~50–100 nM, and centrosome loss at or above 100-250 nM RP-1664 not only in RPE1 cells, but also in three different neuroblastoma cell lines (Fig. 3B; and Supplementary Fig. 2B, C). Since centriole loss underlies the sensitivity of TRIM37-high and WT p53 cells to PLK4i^9,10^, we hypothesized that cytotoxicity observed in neuroblastoma cells at low RP-1664 concentrations is independent of TRIM37 and p53 status. To test this hypothesis, we generated *TRIM37-* and *TP53-*CRISPR/Cas9 *KO* clones in the neuroblastoma cell lines CHP134 and CHP212 (Fig. 3C, D)–as well as a clone of CHP134, referred to as *TRIM37-low*, that retains reduced TRIM37 expression due to residual wild-type alleles (Fig. 3C; and Supplementary Fig. 2D). We also made a CHP134 *TRIM37-low*/*TP53-KO* cell line, which has p53 inactivated on top of lowered TRIM37 levels (Fig. 3C). We then compared RP-1664 sensitivity of these cell lines to parental WT cells (Fig. 3E, F).

Inactivation of p53, lowering TRIM37 levels, and/or ablating TRIM37 completely, reduced RP-1664 sensitivity of both CHP134 and CHP212 cells at concentrations associated with centrosome loss, but did not induce resistance to RP-1664 at lower doses that lead to centrosome amplification (Fig. 3E, F). The rescue of sensitivity to higher, but not lower, PLK4i doses by reducing TRIM37 level in CHP134 cells was reproduced with the published selective PLK4i Centrinone B^36^, ruling out compound-specific effects (Supplementary Fig. 2E–G). Cellular sensitivity to different RP-1664 concentrations correlated with p53 activation: whereas RP-1664 induced p21 expression in CHP134 cells at doses leading to centrosome amplification as well as depletion, lowering TRIM37 levels dampened p21 expression only at centrosome depletion concentrations (Supplementary Fig. 3A, B). Furthermore, whereas a dose of RP-1664 causing centrosome depletion prolonged mitotic duration in CHP134 cells but not CHP134 *TRIM37-low* as expected from the TRIM37-PLK4 synthetic lethality model^10,23^, a dose causing centrosome amplification did not (Supplementary Fig. 3C, D), again suggesting a different mechanism of action. Sensitivity of CHP134 and CHP212 cells to different RP-1664 concentrations was also associated with induction of apoptosis and cell death as measured by Annexin V and Cytotox (reflective of membrane permeability) staining (Supplementary Fig. 3E, F). Altogether, our data suggest that neuroblastoma cells are sensitive to lower and higher PLK4i concentrations in two distinct ways: at lower doses, neuroblastoma cells are killed by PLK4i regardless of *TRIM37* and *TP53* status, whereas at higher doses neuroblastoma sensitivity depends on high TRIM37 and functional p53.

#### *TRIM37-* and *TP53-*independent sensitivity of neuroblastoma tumors to RP-1664 in vivo

To test whether the *TRIM37-* and *TP53-*independent mechanism of sensitivity contributes to anti-tumor activity of RP-1664 in vivo, we engrafted immunodeficient mice with parental CHP134 cells, alongside a *TRIM37-KO* clone (different from the one used in our in vitro studies but showing the same RP-1664 sensitivity, Supplementary Fig. 4A) and a *TP53-KO* clone. We confirmed that the resulting tumors maintained the desired TRIM37 and p53 status (Supplementary Fig. 4B) and treated tumor-bearing mice with either blank chow or 300 ppm RP-1664 chow, which in this model led to a daily free plasma RP-1664 concentration of ≤100 nM on average, consistent with centriole amplification (Supplementary Fig. 4C). This regimen led to marked efficacy in the parental CHP134 model, with regression of 6/7 tumors (Fig. 3G). Interestingly, inactivation of neither *TRIM37* nor *TP53* affected the efficacy of RP-1664 (Fig. 3G), suggesting that the TRIM37 and p53-independent mechanism of sensitivity in CHP134 cells is active in vivo. The treatment was well tolerated in all models with no weight loss observed (Supplementary Fig. 4D). These data suggest that low doses of RP-1664 are sufficient to elicit *TRIM37-* and *TP53-*independent anti-tumor activity in vivo.

#### CRISPR screens confirm a dual mechanism of RP-1664 sensitivity

**Fig. 4:**
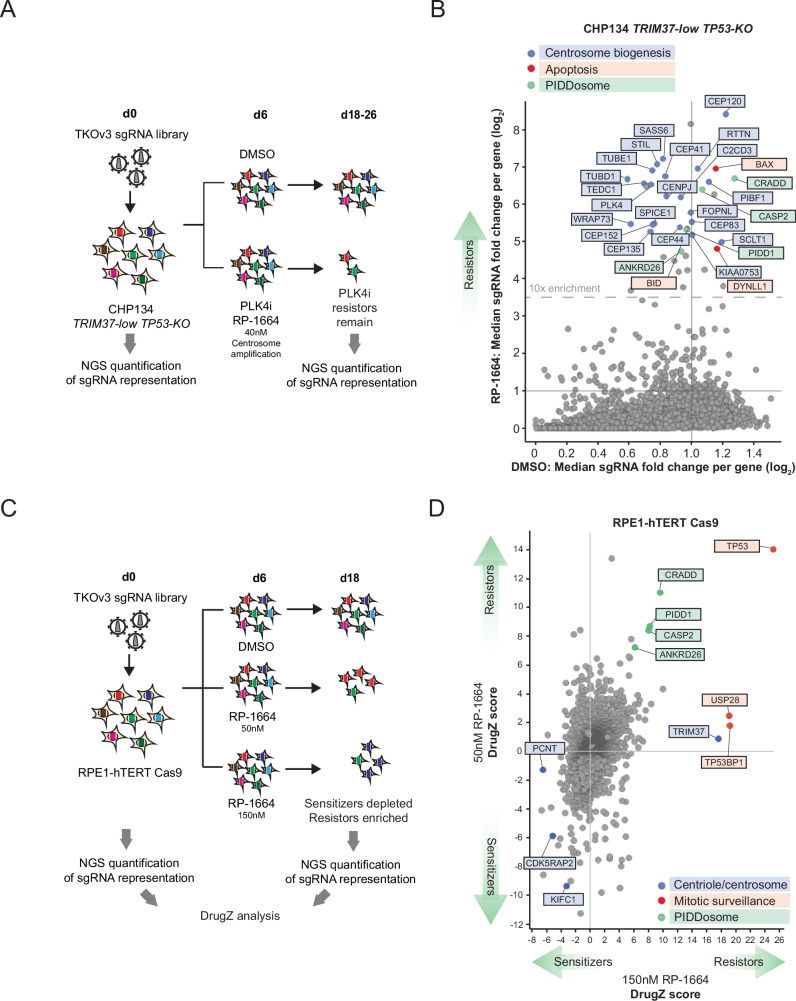
CRISPR screens for genes modulating sensitivity to RP-1664. **A**, **B** Screen for gene knockouts causing RP-1664 resistance in CHP134 *TRIM37-low/TP53-KO* cells. A. Experimental design. See methods for details. **B** Screen results. Median sgRNA fold changes per gene in RP-1664-treated cells (*Y* axis) vs. untreated (*X* axis). Resistor hits with known functions in centriole/centrosome biogenesis (blue), apoptosis (red) or the PIDDosome (green) are highlighted. **C**, **D** Screen for genes knockouts causing RP-1664 resistance or sensitivity in RPE1-hTERT Cas9 cells. **C** Experimental design. See methods for details. **D** Screen results. Gene-level DrugZ^85^ scores in cells treated with 50 nM RP-1664 (*Y* axis) vs. 150 nM (*X* axis). Hits with known functions in centriole/centrosome biogenesis (blue), the mitotic surveillance pathway (red) or the PIDDosome (green) are highlighted. Source Data: Supplementary Data 1 and 2.

Our cellular studies implied that neuroblastoma cells are sensitive to low concentrations of PLK4i that lead to centrosome amplification. We therefore asked whether centrosome amplification per se underlies neuroblastoma sensitivity. To that end, we utilized unbiased CRISPR/Cas9-enabled screening, to map TRIM37- and p53-independent mechanisms of neuroblastoma sensitivity to RP-1664. We transduced CHP134 *TRIM37-low/TP53-KO* cells with the genome-wide TKOv3 Cas9/sgRNA library^38,39^, and treated the resulting cell pool with 40 nM RP-1664 (dose leading to centrosome amplification) or DMSO as a control. Over the course of the screen 40 nM RP-1664 resulted in ~80% loss of cell number as compared to DMSO-treated cells. We used NGS to determine which sgRNAs were enriched in the final RP-1664-treated cell population over the initial cell pool, as these are likely to cause resistance to the compound (Fig. 4A,** Methods**). We identified several genes with median sgRNA representation increased upon RP-1664 compared to DMSO treatment (Fig. 4B, and Supplementary Data 1). Analyzing known functions of these genes revealed striking patterns: Out of 41 genes showing at least 10-fold median sgRNA enrichment, 20 are involved in centrosome biology, 3 regulate apoptosis, and 4 are components of the PIDDosome complex (Fig. 4B). The presence of multiple centrosomal components (e.g., *STIL, CEP120, CEP152* etc.), as well as *PLK4* itself, in the list of hits suggests that blunting the cells’ ability to produce centrosomes alleviates RP-1664 cytotoxicity. The PIDDosome triggers apoptosis upon recognizing supernumerary centrosomes^40,41^ and therefore its inactivation likely allows CHP134 cells to survive despite centrosome amplification. The PIDDosome component caspase 2 is known to induce apoptosis through the pro-apoptotic BCL2 family of factors including BID and BAX^42^, both of which scored as hits in our screen (Fig. 4B). In aggregate, these data implicate centrosome amplification as the bona fide cause of TRIM37- and p53-independent cell death in CHP134 neuroblastoma cells at low concentrations of RP-1664.

To further explore the genetic framework of response to RP-1664, we performed a chemogenomic screen in Cas9-expressing RPE1-hTERT *TP53-WT* cells treated with DMSO, 50 nM RP-1664 (centrosome amplification), or a higher dose, 150 nM RP-1664 (Fig. 4C, D; and Supplementary Data 2). The lower sensitivity of RPE1 cells to RP-1664 compared to CHP134 allowed us to determine not only which gene knockouts lead to RP-1664 resistance, but also which genes, when inactivated, increase RP-1664 sensitivity. The results were in agreement with a published screen performed using Centrinone B^28^ (Supplementary Data 2). We successfully identified *TRIM37*, and the mitotic surveillance factors *USP28* and *TP53BP1*, as required for cell sensitivity to 150 nM RP-1664, consistent with this concentration leading to centrosome depletion. At the same time, PIDDosome components were among the strongest resistor hits at the lower concentration (Fig. 4D; note that the PIDDosome factors scored as hits also in the higher dose arm, albeit not as strongly as *TRIM37* or *TP53BP1*). These data support the interpretation that cells can become sensitive to RP-1664 by two orthogonal mechanisms - centrosome amplification and depletion.

Of note, in RPE1 cells, inactivation of *TP53* caused resistance to both doses of RP-1664 (Fig. 4D). This was confirmed by a screen using cytosine-to-adenine base editor (CBE^FNLS^)^43^-expressing RPE1 cells, and an adaptation of a published sgRNA library that generates 5855 single-nucleotide variants in 298 cancer-related genes (Supplementary Fig. 5A)^44^. *TP53* mutations caused resistance to both 50 nM and 150 nM RP-1664 (Supplementary Fig. 5B, C; and Supplementary Data 3). It should be noted, however, that RPE1 cells with normal TRIM37 levels show only minimal sensitivity to 50 nM RP-1664 (see Fig. 1F) suggesting that enrichment of cells carrying *TP53* sgRNAs in our CRISPR screens may be reflective of altered growth kinetics of *TP53*-deficient cells upon low-dose PLK4i as compared to *TP53*-proficient ones, rather than a bona fide rescue of cell death.

#### Neuroblastoma cells lack mechanisms to tolerate excess centrosomes

Among the genes whose inactivation sensitized RPE1 cells to 50 nM RP-1664 was *KIFC1* (Fig. 4D). Its gene product, KIFC1 (or HSET) is a kinesin motor protein previously implicated in cellular tolerance to supernumerary centrosomes^45,46^. KIFC1 facilitates centrosome clustering if more than two centrosomes are present, enabling formation of a (pseudo-)bipolar spindle^46^. The inability to cluster supernumerary centrosomes may thus underlie sensitivity to low PLK4i concentrations.

**Fig. 5:**
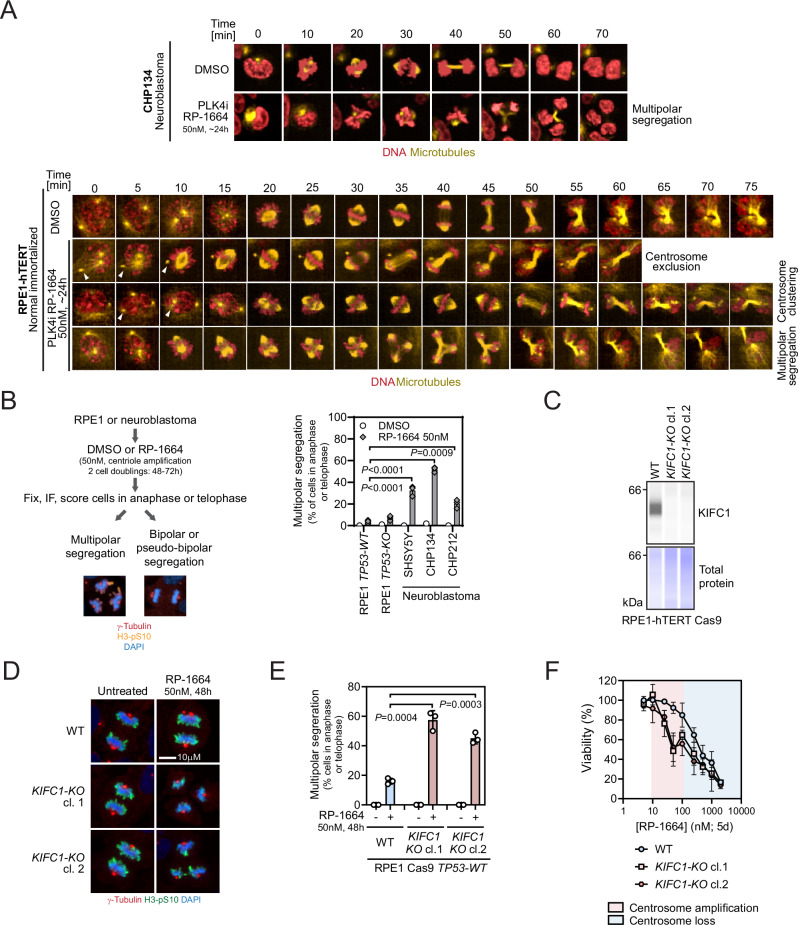
Lack of clustering/exclusion of extra centrosomes leads to RP-1664 sensitivity. **A** Representative tempograms from time-lapse imaging of CHP134 (top) and RPE1-hTERT Cas9 (bottom) cells stained with SPY555-Tubulin for microtubules (yellow) and SPY650-DNA for DNA (red) and treated with DMSO or 50 nM RP-1664. Example cells undergoing normal division, multipolar segregation, or pseudo-bipolar division with centrosome clustering or centrosome exclusion are shown. **B** Left: Workflow for quantification of multipolar segregation frequency. RPE1 or neuroblastoma cells were treated with 50 nM RP-1664, fixed and immunostained for centrosomes (γ-Tubulin) and mitosis (H3-pS10). DAPI was a nuclear counterstain. Anaphase and telophase cells undergoing either bipolar or multipolar division were quantified. Right: Frequency of multipolar segregation in anaphase or telophase with or without RP-1664 in indicated cell lines. Data from *N* = 3 independent experiments (open symbols) with mean (bars) ±SD. *P* value calculated with an unpaired two-sided T-test. **C** Representative (of *N* = 2 independent experiments) KIFC1 capillary immunodetection of *KIFC1-WT* and *KIFC1-KO* RPE1-hTERT Cas9 cells. Total protein is a loading control. **D** Representative micrographs of *KIFC1-WT* and *KO* cells processed for immunofluorescence with γ-Tubulin and H3-pS10 antibodies with or without RP-1664 treatment. DAPI used as a nuclear counterstain. **E** Quantification of *KIFC1-WT* and *KO* cells in anaphase or telophase undergoing multipolar vs. bipolar division in presence or absence of RP-1664. Data from *N* = 3 independent experiments (open symbols) with mean (bars) ±SD. *P* values determined with an unpaired two-tailed T-test. **F** RP-1664 sensitivity of *KIFC1-WT* and *KIFC1-KO* cells. Mean viability measurements from *N* = 3 independent Incucyte growth assays ±SD. Source data: SourceData_Figure5.xlsx and SourceData_uncropped_blots.pdf.

To test this hypothesis, we stained DNA and microtubules in CHP134 cells with fluorescent dyes (SPY650-DNA and SPY555-Tubulin) and followed mitotic progression with or without 50 nM RP-1664 by live cell imaging. As a ‘normal’ cell control we used RPE1-hTERT *TP53-WT* cells. Whereas under unperturbed conditions CHP134 cells divided normally, we observed multipolar segregation once cells were treated with RP-1664 at low nM concentrations (Fig. 5A; and Supplementary Video 1, 2). In contrast, RPE1-hTERT cells displayed a wider range of phenotypes upon RP-1664 treatment: In addition to occasional multipolar segregation, RPE1 cells also performed pseudo-bipolar mitoses with supernumerary centrosomes, utilizing either centrosome clustering or exclusion (Fig. 5A; Supplementary Video 3–5)–both known mechanisms of adaptation to extra centrosomes^47–49^. To quantify the frequency of multipolar and bipolar mitoses in neuroblastoma versus normal cells upon RP-1664 treatment, we fixed and stained with anti-γ-Tubulin and H3-pS10 antibodies three neuroblastoma cell lines (CHP134, CHP212, SHSY5Y), as well as RPE1 controls, and counted the frequency of cells in anaphase or telophase that underwent bipolar versus multipolar segregation (Fig. 5B). Whereas immortalized RPE1 cells infrequently displayed multipolar mitosis in presence of 50 nM RP-1664, multipolar mitoses were common in neuroblastoma cells, suggesting that it is indeed the inability to cope with supernumerary centrosomes that underlies PLK4i hypersensitivity (Fig. 5B).

To examine whether disrupting centrosome clustering would sensitize the normally resistant RPE1 cells to low dose PLK4i, we CRISPR-inactivated *KIFC1* in RPE1-hTERT Cas9 *TP53-KO* cells and confirmed that the frequency of multipolar cell divisions robustly increased in two *KIFC1-KO* clones upon 50 nM RP-1664 treatment (Fig. 5C-E). Consistent with our CRISPR screen data, *KIFC1-KO* cells were more sensitive to doses of RP-1664 causing centrosome amplification than parental cells, whereas sensitivity to doses leading to centrosome depletion was comparable (Fig. 5F). We conclude that the ability to cluster (or inactivate) supernumerary centrosomes governs cellular sensitivity to partial PLK4 inhibition.

#### RP-1664 shows potent single-agent efficacy in human neuroblastoma-derived xenograft models

**Fig. 6:**
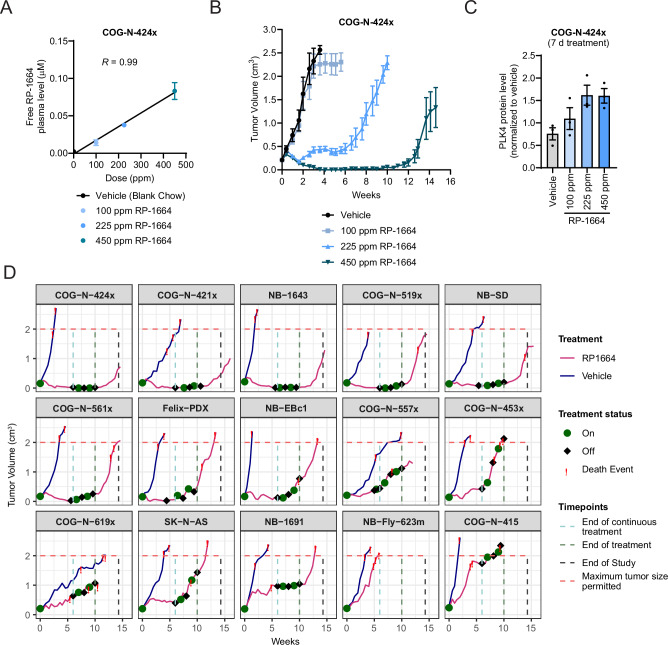
Potent single-agent efficacy of RP-1664 in neuroblastoma xenograft models. **A** Free (not bound to plasma protein) plasma concentrations of RP-1664 in mice treated with indicated doses of RP-1664 chow. Mean of *N* = 3 mice ±SEM. **B** Tumor volume measurement of COG-N-424x mouse xenografts treated with blank chow or indicated doses of RP-1664 chow using a continuous dosing schedule of RP-1664 for six weeks, followed by two cycles of one week on and one week off as detailed in method section. Mean of *N* = 6 mice/group ±SEM. **C** PLK4 protein level quantification by capillary immunodetection in lysates from COG-N-424x tumors treated for 7 days with indicated doses of RP-1664. Mean of *N* = 3 mice ±SEM. **D** Tumor volume measurements in 15 xenograft models of high-risk neuroblastoma treated with vehicle or 450 ppm of RP-1664 chow. Treatment periods and endpoint events are indicated. Data are mean of *N* = 3 mice/group. Source data: SourceData_Figure6.xlsx.

Next, we sought to determine whether the dual sensitivity of neuroblastoma cells to centrosome amplification and depletion translates broadly into anti-tumor activity and tolerability in vivo. We deployed a panel of fifteen human neuroblastoma xenograft models (5 CDX and 10 PDX), all showing 17q gain including the *TRIM37* locus (Supplementary Table 2**;** no xenograft models without 17q gain were available). To explore the range of efficacious RP-1664 doses, we first performed a dose-response study in one of our models, COG-N-424x, at 100, 225, and 450 ppm RP-1664 chow. The RP-1664 chow formulation showed pharmacokinetic dose-linearity across 100 ppm, 225 ppm, and 450 ppm (Fig. 6A). After three days of dosing, 225 and 450 ppm achieved a free plasma RP-1664 concentration of 38 ± 5 and 83 ± 19 nM, respectively (Fig. 6A)–at or above the median cell line IC_50_ previously measured in growth assays. Consistent with this observation, RP-1664 showed dose-dependent anti-tumor activity, with minimal tumor growth inhibition at 100 ppm, tumor regression at 450 ppm, and intermediate efficacy at 225 ppm (Fig. 6B, Supplementary Fig. 6A, B). To assess whether this efficacy correlated with target engagement and modulation by RP-1664, we analyzed PLK4 protein stabilization and p21 expression in COG-N-424x tumors by capillary immunodetection and immunohistochemistry, respectively. Doses that induced anti-tumor activity elevated both PLK4 and p21 protein levels in a dose- and time-dependent manner, confirming target modulation by RP-1664 at biologically active exposures (Fig. 6C and Supplementary Fig. 6C–E). Target engagement was also confirmed in a second model, COG-N-421x (Supplementary Fig. 6F–H). Importantly, we observed multipolar mitoses in RP-1664-treated COG-N-421x tumors but rarely in vehicle-treated samples, supporting the idea that centrosome amplification by RP-1664 may contribute to its in vivo efficacy (Supplementary Fig. 6I, J).

Next, we explored RP-1664 efficacy across the entire fifteen-model panel. We chose a 450 ppm dose of RP-1664, which elicited tumor regression in our dose-response experiment and was in a historically well-tolerated range on both continuous and intermittent schedules (Fig. 1G)^27^. Mice with established xenografts averaging 200 mm^3^ were randomized to receive RP-1664 chow at 450ppm or standard chow, *N* = 3/arm, with the treated mice exposed continuously for 42 days, followed by an intermittent, 7 days on/7 off, schedule until day 70. Using response criteria established by the Pediatric Preclinical Testing Consortium^50,51^, 14/15 (93%) of the xenografts showed evidence of anti-tumor activity, with an objective response rate (ORR) of 53% (seven complete responses, two of which were maintained), one partial response, and six significant growth delays extending survival (Supplementary Data 4, Fig. 6D, Supplementary Fig. 7A, B). RP-1664 was generally well tolerated, with mice harboring three xenografts requiring brief interruptions in dosing due to >15% weight loss (Supplementary Fig. 7C). No clear biomarker of RP-1664 response was evident on review of candidate markers examined, and notably two *TP53* mutated models (COG-N-519x, NB-SD) showed robust responses (Fig. 6D).

#### RP-1664 monotherapy significantly increases survival in an immunocompetent mouse model of neuroblastoma

**Fig. 7:**
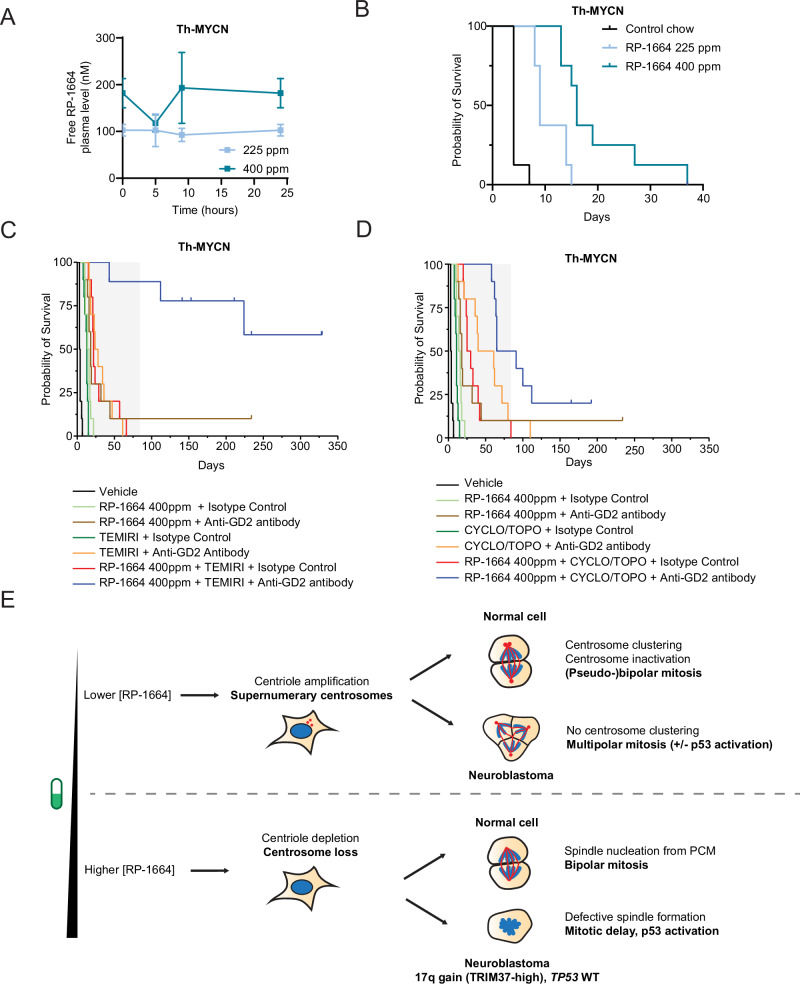
RP-1664 extends survival of immunocompetent mice with spontaneous neuroblastomas. **A** Free (not bound to plasma protein) plasma concentrations of RP-1664 in Th-MYCN mice treated with indicated doses of RP-1664 chow. Mean of *N* = 4 mice at indicated time points ±SEM. **B** Kaplan-Meier survival curves showing survival of Th-MYCN mice treated with blank chow or RP-1664 at presentation of small palpable tumor. *N* = 8 mice/group. **C**, **D** Kaplan-Meier survival curves showing survival of Th-MYCN mice upon indicated treatments at presentation of small palpable tumor. *N* = 10 mice/group. **E** A two-component model of neuroblastoma sensitivity to PLK4i. See main text for details. Source data: SourceData_Figure7.xlsx.

We next tested the efficacy of RP-1664 in an immunocompetent setting, using a genetically engineered model of neuroblastoma. The Th-MYCN mouse model, targeting human *MYCN* expression to the neural crest, recapitulates poorly differentiated late-stage neuroblastoma^52,53^. All mice homozygous for the *MYCN* transgene develop tumors by 7 weeks of age^53,54^. We treated tumor-bearing mice with control chow, or RP-1664 chow at 225 and 400ppm. As we observed sporadic body weight loss in our prior xenograft panel upon continuous dosing, we delivered the RP-1664 chow on an intermittent, 14 days on/7 days off schedule. In the Th-MYCN model these doses led to average free RP-1664 plasma concentrations of 100 ± 5 nM and 169 ± 34 nM (Fig. 7A), covering concentrations associated with centriole amplification and loss, respectively. Mice treated with RP-1664 demonstrated extension of survival in a dose-dependent manner. Mice on control chow had an average survival of 4.375 days, which was extended to 10.75 and 19.5 days in the 225 ppm and 400 ppm RP-1664 treated groups, respectively (*n* = 8/arm; *P* < 0.0001 at both doses compared to control, Fig. 7B). The higher dose also significantly extended survival over the lower dose (*P* = 0.0031). Tumor regression was observed in 3/8 animals on the higher dose, while the remainder demonstrated slower tumor progression. Weight loss was minimal over the course of treatment; however, weights did fluctuate when mice cycled on and off the medicated chow (Supplementary Fig. 8A). Target engagement was confirmed at both doses of RP-1664, as we observed stabilization of the mouse PLK4 protein in RP-1664-treated tumors (Supplementary Fig. 8B, C).

#### RP-1664 combined with GD2-directed chemoimmunotherapy shows potent efficacy in the Th-MYCN model

Next, we treated Th-MYCN mice with 400 ppm RP-1664, as above, in combination with either 2 days of 2 mg/kg irinotecan and 5 mg/kg temozolomide (TEMIRI) or 2 days of 10 mg/kg cyclophosphamide and 0.5 mg/kg topotecan (CYCLO-TOPO), each with or without 2 doses of 15 µg anti-GD2 antibody (a minimal schedule designed to demonstrate combinatorial effects). Survival curves for all groups are shown in Fig. 7C,D, with objective responses and statistical tests in Supplementary Fig. 8D and Supplementary Table 3. Combining RP-1664 with TEMIRI enhanced response and extended median survival (18.5 days) compared to RP-1664 (15 days) and TEMIRI (13 days). RP-1664 combined with CYCLO-TOPO extended median survival to 27.5 days compared to CYCLO-TOPO alone at 11 days. Strikingly, the addition of anti-GD2 antibody to RP-1664/TEMIRI induced deep and durable complete and long-term tumor-free survival in 6/9 mice. The addition of anti-GD2 antibody to RP-1664/CYCLO-TOPO was less striking, but still substantial benefit, with MCRs in 2/10 mice, CRs in 7/10 mice, and extension of median survival to 78 days.

We conclude that tolerable doses of RP-1664 are broadly efficacious across multiple pre-clinical models of high-risk neuroblastoma, and that clinical development of combinations with standard of care chemoimmunotherapy may benefit patients.

## Discussion

PLK4 inhibition was suggested as a therapeutic strategy for high-risk neuroblastoma due to a high prevalence of *TRIM37* gain^10^. We now demonstrate that 17q/*TRIM37* gain is more prevalent than previously reported, suggesting that selection of neuroblastoma patients based on *TRIM37* copy number status may not be necessary. We then showed that the PLK4i RP-1664 therapy produces robust single agent anti-tumor activity across a spectrum of high-risk neuroblastoma pre-clinical models reflecting the genetic heterogeneity of this disease. However, this exquisite PLK4 sensitivity cannot be attributed solely to *TRIM37* overexpression. We uncovered that PLK4i sensitivity of neuroblastoma cells is in fact a composite of two complementary but distinct mechanisms. At lower doses, PLK4i (including RP-1664) amplify centrosomes through stabilization of partially active PLK4^28–33^. Neuroblastoma cells are unable to tolerate supernumerary centrosomes, as they do not employ centrosome clustering or exclusion. In turn, neuroblastoma cells undergo multipolar segregation, presumably leading to aneuploidy and genomic instability (Fig. 7D). On the other hand, at higher PLK4i concentrations neuroblastoma cells lose centrosomes and succumb to mitotic delay or failure as a consequence of high TRIM37 levels^10^ (Fig. 7D). This dual mechanism makes neuroblastoma an attractive target population for PLK4 inhibition therapy such as RP-1664 and adds to a growing list of factors predictive of PLK4i sensitivity, including high *TRIM37* levels^9,10^ or β-catenin hyperactivation^55^, the latter of which is rarely observed in high-risk neuroblastoma.

We demonstrate that centrosome clustering is a bona fide mechanism of resistance to PLK4i, as inactivation of a centrosome clustering factor, *KIFC1*, sensitizes normally resistant cells to low dose RP-1664. However, an important outstanding question is what exact genetic basis underlies the inability to compensate for supernumerary centrosomes in neuroblastoma. In addition to *KIFC1*, over 30 genes are already known to facilitate centrosome clustering^47^, including factors involved in microtubule organization, the spindle assembly checkpoint, sister chromatid cohesion, as well as the actin cytoskeleton and cell adhesion^46,48,56–60^. Future studies will be needed to assess the mutational status or altered expression of these genes in neuroblastoma and whether these contribute to centrosome clustering defects. Similarly, it will be important to determine whether any of the recurrent genetic alterations in neuroblastoma such as *MYCN* amplification^1^, *ALK* gain-of-function mutation/amplification^1^, *LIN28B* overexpression^61,62^, and chromosome 1p or 11q loss^63^, contribute to defects in centrosome clustering. Multiple potential links between these alterations and centrosome biology exist. For example, we showed that *LIN28B* overexpression has been linked to overactivation of the mitotic kinase Aurora A (AURKA)^61^ involved in centrosome maturation, spindle assembly and cytokinesis^64^. *MYCN* amplification has been previously associated with centrosome amplification^65,66^, but another study did not corroborate these data^67^. Future correlative studies using our high-risk neuroblastoma models may prove useful in uncovering the genetic mechanisms that govern PLK4i sensitivity or resistance. Of note, in a recent pre-print, Moreno-Marin et al. describe a remarkable diversity in centrosome clustering proficiency among the NCI60 cancer cell line panel, suggesting that sensitivity to centrosome overduplication may be found in additional tumor lineages^68^.

Our study also brings further support for a complex role of the p53 pathway in the response to abnormal centrosome numbers. Whereas in neuroblastoma cells p53 was dispensable for cell death at doses of RP-1664 causing centrosome amplification, p53 inactivation reduced sensitivity of RPE1 cells at equivalent RP-1664 concentrations. The simplest reconciliation of this apparent discrepancy is that cell death upon centrosome amplification can occur both in a p53-dependent and p53-independent fashion, consistent with the complete responses in two *TP53* mutant xenograft models. Our data implicate the PIDDosome and caspase 2 in mediating RP-1664 cytotoxicity. Canonically, the PIDDosome is activated by recruitment to distal appendages of supernumerary centrioles by ANKRD26, leading to caspase 2-mediated cleavage of the p53 inhibitor MDM2^69^. However, caspase 2 can trigger apoptosis also in a p53-independent manner, through release of pro-apoptotic factors (such as BID or BAX) from mitochondria^70^. It is therefore possible that this pathway contributes to PLK4i cytotoxicity in neuroblastoma when p53 is inactivated, which is consistent with our CRISPR screen observations.

The contribution of centriole overduplication to PLK4 inhibitor efficacy raises important translational questions. It is not unreasonable to imagine that in mice and humans both mechanisms of sensitivity may operate in parallel, depending on the dose, schedule, pharmacokinetic profile of a given inhibitor and local concentration of RP-1664 in each tumor area. While monitoring centrosome numbers in tumor tissues is non-trivial^71^, emergence of multipolar mitoses may provide a useful pharmacodynamic readout during PLK4 inhibitor clinical dose optimization and should be taken into consideration when interpreting clinical data in distinct patient populations (e.g., neuroblastoma vs. other TRIM37-overexpressing tumor types, in which higher-dose depletion strategies may predominate). Future studies will be aimed at unraveling the mechanistic basis of the TRIM37-dependent and -independent centriole deregulation in neuroblastoma as compared to other human cancers.

The single agent anti-tumor activity seen in this study with an ORR of 53% compares favorably with the historical ORR of 14% seen in the NCI preclinical testing program^71^. Indeed, across the 20-year history of this program for in vivo screening of anti-cancer agents, the majority of drug showed an ORR of 0, with higher response rates in more recent years to antibody drug conjugates with potent payloads^50^. The most robust single agent activity reported to date in high-risk neuroblastoma preclinical models is the ALK inhibitor lorlatinib specific to ALK mutated models^72^, where a clear mechanistic basis was tested and translated to robust clinical activity^73^. Our data now extend this paradigm by showing that RP-1664 retains substantial activity in an immunocompetent Th-MYCN model and, importantly, that combining RP-1664 with standard-of-care TEMIRI- or CYCLO-TOPO–based chemoimmunotherapy significantly enhances efficacy. In particular, the addition of anti-GD2 antibody to RP-1664/TEMIRI induced durable, long-term tumor-free survival in most treated mice, highlighting a strong functional interaction between PLK4 inhibition and GD2-directed immune effector mechanisms. These findings argue that PLK4 inhibitors may be most effective when integrated into multi-agent chemoimmunotherapy regimens rather than used in isolation. Future studies will focus on testing the hypothesis that RP-1664 sensitizes the innate and/or adaptive immune system to synergistically enhance the efficacy of GD2-directed chemoimmunotherapy.

With the extremely high prevalence of 17q gain in high-risk neuroblastoma, there is a tremendous opportunity for biomarker-directed clinical development of RP-1664 building on the striking preclinical activity of RP-1664 in combination with standard-of-care chemoimmunotherapy in the relapse setting. As chemoimmunotherapy is being incorporated into frontline induction therapy, incorporation of RP-1664 may ultimately provide a rational therapeutic strategy to improve initial response rates and potentially enable de-escalation of the current highly dose-intensive chemotherapy standard of care.

## Methods

### Cell culture

Cell lines were purchased from the following vendors: RPE1, MCF7, CHP212, SHSY5Y, SKNAS, SKNDZ, SKNFI, SKNSH, HK2 - ATCC; KELLY, MCF10A – Sigma Aldrich; CHP134, IMR32 – DSMZ, COL-hTERT, BRONCH2 – Applied Biological Materials. RPE1-hTERT Cas9 *TP53-WT* and *TP53-KO* were described before^74,75^. Cells were cultured at 37 °C and 5% CO_2_ in the following media (all supplemented with 10% fetal bovine serum (FBS; VWR, 76419-584), 100 U/ml penicillin and 100 mg/ml streptomycin (Pen/Strep; Corning, 30-001-CI), unless indicated otherwise): RPE1, CHP212, CHP134, SKNDZ, SKNFI, COL-hTERT, HK2 – DMEM (Corning, 10-013-CV); IMR32, SKNAS, KELLY, BRONCH2 – RPMI (Corning, 10-104-CV); MCF7 – EMEM (Corning, 10-009-CV) + 1% non-essential amino acids + 10 mg/ml insulin; SKNSH – MEM (Corning 10-010-CV); SHSY5Y – DMEM: F12 (1:1; Corning 10-090-CV); DMEM: F12 (1:1), 5% horse serum (Gibco, 16050122), 20 ng/ml EGF (Thermo Fisher, PHG0311L), 500 μg/ml hydrocortisone (StemCell Technologies, 07925), 10 μg/ml insulin (Millipore-Sigma, I9278-5ML) and Pen/Strep.

The breast cancer cell line panel in Supplementary Fig. 2A was maintained by Crown Bio, Inc. The cell lines were cultured at 37 °C and 5% CO_2_ in the following media: RPMI-1640 + 10% FBS + Pen/Strep - HCC1500, DU4475, ZR751; HCC1428, ZR7530, MX1; MEM + 10% FBS + 0.1 mM non-essential amino acids + Pen/Strep - CAMA1; RPMI-1640 + 20% FBS + Pen/Strep – EFM192A; MEM + 10% FBS + 0.1 mM non-essential amino acids + 10μg/ml bovine insulin + Pen/Strep – MCF7.

### Genomic and transcriptomic data analysis

The whole genome sequencing data (WGS) utilized in this project was obtained through the Gabriella Miller Kids First Project (dbGaP phs001436.v1.p1)^76^. Genomic sequencing was performed at Hudson Alpha Institute for Biotechnology (DNA) and St. Jude Children’s Research Hospital’s Genomic Sequencing Laboratory (RNA). WGS was performed using Illumina’s HiSeq X System at a mean coverage of 30x. Whole exome sequencing (WES) was performed using Illumina’s HiSeq 4000 System at a mean coverage of 150x. Total RNA sequencing (RNA-seq) was performed using Illumina’s HiSeq 4000 System.

Tumor DNA samples with a confirmed sequencing coverage of at least 20x were subjected to consensus somatic copy number variant (CNV) analysis. Tumor/normal sample pair concordance was verified using NGSCheckMate^77^. CNV calling was performed using Control-FREEC^78^, CNVkit^79^, and GATK^80^ to enable consensus-based CNV detection, as previously described^81^. RNA-Seq data comprising a minimum of 20 million total reads, with at least 50% of reads successfully aligning to the human genome. RNA reads were aligned to the HG38 reference genome using STAR^82^. Gene level quantification was performed using RSEM^83^ with gene annotations based on Gencode release v39.

Validation of 17q copy number gain status in CHOP tumor samples was undertaken under Children’s Hospital of Philadelphia Institutional Review Board (IRB) protocol 25-023583. Sequencing was performed using targeted capture DNA sequencing using Illumina’s HiSeq 4000 at a mean coverage of 2000x. Sequence data was analyzed for copy number analysis using NextGENe V2 NGS analysis software and visual inspection.

### CRISPR/Cas9 knockout of *TRIM37*, *TP53, KIFC1*

Cells were transfected with Cas9:sgRNA complexes using Lipofectamine CRISPRmax (Thermo Fisher Scientific) according to manufacturer’s protocol, allowed to recover for 2-3 days and then seeded for clonogenic outgrowth. Knockout (KO) clones were characterized by Sanger sequencing, ICE analysis^84^ and/or immunoblotting/Simple Western analysis. The following sgRNA target sequences were used: *TRIM37* – TCGCATCAGTGTGCACTTTG or GATGAAGTAAATCAGCTCGA; *TP53* – CAGAATGCAAGAAGCCCAGA; *KIFC1* – GTCCCCCCTATTGGAAGTAA. sgRNA oligonucleotides were synthesized by Integrated DNA Technologies.

### Chemical compounds

RP-1664 was synthesized by Repare Therapeutics^27^. Centrinone B was purchased from MedChem Express. Compounds were made up at stock concentrations of 10 mM from powder in dimethyl sulfoxide (DMSO) and kept at -20 °C for long-term storage.

Cyclophosphamide (Baxter Healthcare, Australia), topotecan (Sandoz, Australia), irinotecan (Accord Healthcare, Australia) and temozolomide (Sigma-Aldrich, USA) were diluted from stock using 5% glucose. In vivo monoclonal antibodies to GD2 (14G2a) (BE0318) and isotype control (IgG2a)(BE0085) were from BioXCell, USA.

### Simple Western capillary immunodetection

For cell lysate preparation, 150,000 cells/well were plated in 12-well plates (Falcon, 353043). Where applicable, increasing doses of RP-1664 were added to the cells using a Tecan D300E dispenser the next day. 24 h later, cells were washed with 1 ml PBS and lysed in-plate with 50 µl of RIPA buffer (Sigma, R0278-50ML), supplemented with 1X protease and phosphatase inhibitors (Thermo-Fisher, 78440).

Xenograft tumor lysates were prepared as follows: Lysis buffer (MSD Tris + 1x protease and phosphatase inhibitors) was added to tumor samples at a w/v ratio of 1:9 (tumor: buffer) and tissue was homogenized using an OMNI bead ruptor 24 bioruptor (speed 5.5 m/s, 3 cycles, 10 s/cycle, 3 min break between cycles on ice). Homogenate was transferred to fresh Eppendorf tubes and cleared by centrifugation at 13,000RPM / 16,000 × *g*, 5 min at 4 °C. Clarification steps were repeated until lysate was completely clear.

Following lysis, protein concentrations were measured using a DC Protein Assay Kit (Bio-Rad, 5000112) and protein levels in all samples were normalized to 1 µg/µl. Immunodetection was performed using a Simple Western JESS instrument (Bio-Techne) according to the manufacturer’s instructions. 3 µl of lysate was loaded per capillary and proteins were detected with their respective primary antibodies (see below). Separation time was set to 25 min, voltage to 375 V, primary antibody incubation to 90 min, and secondary antibody incubation to 60 min.

### Immunoblotting

Cells were lysed in 2x Novex Tris-Glycine SDS sample buffer + 200 mM DTT (Thermo Fisher Scientific LC2676) at 1×10^6^ cells/ml and boiled at 95 °C for 5 min. 20-30 ml of lysate were run on Novex Tris-Glycine SDS gels (Thermo Fisher Scientific). Proteins were transferred to 0.2 mM nitrocellulose membranes at 90 V for 2-2.5 h. Membranes were blocked with 5% milk/TBST for 30 min at room temperature (RT) and incubated with primary antibodies at 4 °C overnight. Membranes were washed 3x 5 min with TBST and incubated with secondary antibodies for 1 h at RT, after which they were washed and developed using the Pierce ECL Pico or Femto Western Blotting substrate (Thermo Fisher Scientific). Chemiluminescence was detected on an Amersham Imager 680 instrument (GE).

### Immunofluorescence

Cells were seeded on black, clear bottom, poly-D-lysine coated 96-well plates (PhenoPlate 96; Revvity, 6055500) and RP-1664 was added the following day using a Tecan D300E dispenser. 48-72 h later, cells were rinsed with PBS and fixed with 4% paraformaldehyde/PBS (PFA; Thermo Fisher, J19943.K2) for 10 mins at RT. Plates were rinsed 2x with PBS and stored at 4 °C. For immunofluorescent staining, cells were permeabilized with 0.3% Triton X-100/PBS for 30 min, and rinsed 2x with PBS. Plates were blocked with blocking buffer (5% goat serum / 0.1% Triton X-100 / PBS) for 1 h. Cells were then incubated overnight at 4 °C or 2 h at RT with primary antibodies in blocking buffer. Next day, plates were rinsed 3x with 0.1% Triton X-100/PBS and incubated for 1 h at RT with secondary antibodies supplemented with 0.5 mg/ml 4′,6-diamidino-2-phenylindole (DAPI). Plates were rinsed 3x with 0.1% Triton X-100/PBS and fresh PBS was added to image on an Operetta CLS or Opera Phenix automated high-content microscope in confocal mode, using a 40x NA1.1 water immersion objective (Revvity).

### Cell growth assays

Cells were seeded on white, clear bottom 96-well plates (Corning, 3903) and compounds were added the next day using a Tecan D300E dispenser. The following seeding densities were used (cells/well): RPE1 – 300; CHP134 – 1500; CHP212 – 1000; SHSY5Y – 1000; IMR32 – 700; SKNFI – 3000; SKNSH – 500; SKNDZ – 2000; KELLY – 700; SKNAS – 700-800; MCF10A – 800; COL-hTERT – 800; BRONCH2 – 600; HK2 - 1000. HCC1500 - 6000; CAMA1 - 1000; DU4475 - 1200; ZR751 - 1500; EFM192A - 1000; HCC1428 - 1500; ZR7530 - 2500; MX1 - 800; MCF7 - 420. When cells reached near-confluence ( ~ 4-6 population doublings / ~ 6-12 days), plates were imaged on an Incucyte S3 automated microscope. Images were analyzed using the Incucyte 2022B software to determine % confluence. Alternatively, cells were processed using the CellTiter Glo cell viability assay (Promega) according to manufacturer’s instructions. Luminescence was measured with a FlexStation 3 plate reader. Data were normalized to untreated controls and IC_50_ values were obtained by non-linear least squares fitting of the data to a four-parameter dose-response model in GraphPad PRISM 10.

### Cytotox and Annexin V cell viability assays

Cells were seeded on black poly-lysine coated 96-well plates (Revvity, 6055500). Two days later, cells were incubated with Incucyte dyes to detect cell death (Cytotox; Sartorius 4633; 1:500) or apoptosis (Annexin V; Sartorius 4642; 1:2000). RP-1664 was added to the plate using Tecan D300E. Plates were imaged on an Incucyte S3 instrument in both phase-contrast and green fluorescent channels every 8 h for 5 days and analyzed using Incucyte 2022B software. Cell death induction by RP-1664 was calculated as the % area stained with green dye (Cytotox or Annexin V) normalized to % confluence, and relative to DMSO.

### Antibodies

The following antibodies and dilutions were used for immunoblotting (IB), immunofluorescence (IF) or capillary immunodetection (JESS): rabbit anti-PLK4 E6A7R (Cell Signaling Technologies 71033; JESS 1:200), rabbit anti-TRIM37 (Bethyl A301-174A; JESS 1:50), mouse anti-p53 DO-1 (sc-126; IB 1:1000), rabbit anti-p21 12D1 (Cell Signaling Technologies 2947; IF 1:500, JESS 1:300), rabbit anti-g-Tubulin EPR16793 (Abcam 179503; IF 1:500), mouse anti-H3pS10 3H10 (Sigma Aldrich 05-806; IF 1:1000), rabbit anti-KIFC1 (ProteinTech 20790-1-AP; JESS 1:50), rabbit anti-DYKDDDDK tag (FLAG) D6W5B (Cell Signaling Technologies 14793; IB 1:1000), rabbit anti-Vinculin E1E9V (Cell Signaling Technologies 13901; IB 1:5000), Alexa Fluor 488/555/647-conjugated goat anti-rabbit or anti-mouse IgG (H + L) (Thermo Fisher Scientific A-11008/A-21428/A-21245/A-11001/A-21422/A-21235; IF 1:500-1:1000), HRP-conjugated goat anti-mouse IgG (BioRad L005680; 1:5000 IB), HRP-conjugated goat anti-rabbit IgG (Jackson ImmunoResearch 111-035-144; IB 1:5000), HRP-conjugated anti-rabbit secondary antibody (Bio-Techne 042-206; JESS undiluted).

### Live cell imaging

Cells were seeded on 96-well poly-lysine coated PhenoPlates (Revvity, 6055500) and incubated in phenol red-free media supplemented with SPY650-DNA (Cytoskeleton CY-SC501) and SPY555-Tubulin (Cytoskeleton CY-SC203) at 1:6000 dilution for 2 h before imaging. RP-1664 or DMSO treatments were added 24 h prior and maintained during imaging. Time-lapse images were captured on an Opera Phenix automated high-content microscope in confocal mode at 37 °C and 5% CO_2_, using a 40x NA1.1 water immersion objective (Revvity). Images were captured every 4–5 minutes for ~6 h and analyzed manually using Harmony. Mitotic duration was recorded for each dividing cell, counted as the time from nuclear envelope breakdown to mitotic exit (i.e., anaphase or chromatin decondensation).

### CRISPR/Cas9 chemogenomic screening

CRISPR/Cas9 screens were performed according to a published protocol^39^. Specifically, The RP-1664 resistance screen was performed in a CHP134 *TRIM37-low TP53-KO* neuroblastoma cell line. Cells were transduced with lentivirus carrying the TKOv3 sgRNA library at a multiplicity-of-infection (MOI) of ~0.3. The screen was conducted in technical duplicates, and library coverage of >100 cells per sgRNA was maintained at every step. Puromycin-containing medium (0.5 µg/ml) was added 2 days after infection to select for transductants. Selection was continued until 96 h after infection, which was considered the initial time point (*t*_0_). 40 nM RP-1664 was added to the cells starting from day 6 (*t*_6_). From *t*_10_ onwards, RP-1664-containing medium was refreshed every four days until the screen was terminated at *t*_26_. To identify genes whose deletion caused resistance to RP-1664, genomic DNA was isolated from surviving cells using the QIAamp Blood Maxi Kit (Qiagen) and genome-integrated sgRNA sequences were amplified by PCR using NEBNext Ultra II Q5 Master Mix (New England Biolabs). i5 and i7 multiplexing barcodes were added in a second round of PCR and final gel-purified products were sequenced on an Illumina NextSeq500 system to determine sgRNA representation in each sample.

The RPE1-hTERT Cas9 *TP53-WT* screen was performed with cells transduced with the TKOv3 sgRNA library at an MOI of 0.4. Technical duplicates for each condition and library coverage of >400 cells per sgRNA were maintained throughout the screen procedure. 2 days post-infection, transduced cells were selected with 2 mg/ml puromycin. As above, the initial timepoint (*t*_0_) was 96 h post-infection. At (*t*_6_), RP-1664 was added to the cells at 50 nM and 150 nM. Compound-containing media was refreshed every four days until the screen reached 18 days. Genomic DNA was isolated at every timepoint, and processed as described above. Sample data analysis was performed using the DrugZ (https://github.com/hart-lab/drugz)^85^ algorithm.

### Base editing screens

RPE1-hTERT *TP53-WT* stably expressing CBE^FNLS^ ^43^ were transduced with lentivirus carrying a modified HBES^44^ sgRNA expression library (lacking the sensor) at MOI of ~0.3. The screen was conducted in technical triplicates, and library coverage of >1,000 cells per sgRNA was maintained at every step. Puromycin-containing medium (2 µg/ml) was added 2 days after infection. Selection was continued until 96 h after infection, which was considered the initial time point (*t*_0_). RP-1664 was added to the cells at day 6 (*t*_6_) and 10 (*t*_*10*_) at the indicated concentrations and the screen was terminated at *t*_18_. Sample processing was carried out as in CRISPR/Cas9 chemogenomic screening. FASTQ files were aligned to the library reference sequences using Bowtie to generate read counts for each sample replicate at the guide level. Raw read counts from the *t*_*18*_ timepoint of each treatment condition were compared to DMSO control at T18 using DESeq2 to calculate log_2_FoldChange values, and the PCR2 forward primer used for each sample replicate was modeled as a covariate.

### MCF7 and CHP134 xenograft studies

MCF7 mouse xenograft studies were performed at Oncodesign under regulations from the Canadian Council on Animal Care and the National Research Council Guide. Female BALB/c Nude mice were supplemented with estradiol in drinking water (2.5 µg/mL) and gamma irradiated (1.2 Gy) 1 week and again 24–72 h prior to inoculation in the right flank using 10 million cells. Animals were placed under blank chow for acclimatization 3–5 days prior to randomization. Mice were randomized into 6 groups of 6 animals each when tumor volume reached a mean of 100–200 mm^3^ and treatment with RP-1664 formulated chow began thereafter. Body weights and tumor volume were both measured twice a week, the latter being assessed with calipers. Tumor volume was calculated using the formula 0.52 × L × W^2^, with percent tumor growth inhibition (% TGI) defined as: % TGI= ((TVvehicle_last_ – TVvehicle_day0_) - (TVtreated_last_ – TVtreated_day0_)) / (TVvehicle_last_ – TVvehicle_day0_) × 100. Percentage changes in body weight (% BW) were calculated using the formula: % BW change = (BW_last_-BW_day0_/ BW_day0_) × 100.

CHP134 mouse xenograft studies were performed at Repare Therapeutics in a vivarium accredited by the Canadian Council on Animal Care with an Institutional Animal Care Committee-approved protocol. Female CB/17 SCID mice were inoculated in the right flank with 10 million cells of either parental CHP134, CHP134 *TP53-KO* or CHP134 *TRIM37-KO* and animals placed under blank chow for acclimatization 3–5 days prior to randomization. Mice were randomized into groups of 7 animals per cell line when tumor volume reached a mean of 100–150 mm^3^. Treatment with RP-1664 formulated chow began thereafter. Body weights and tumor volume were measured three times a week, the latter being assessed with digital calipers. Tumor volume, growth inhibition and body weight changes were calculated as above. Maximal tumor size endpoint for this study was 1500 mm^3^, below the IACUC maximum tumor volume of 2000 mm^3^.

### Human neuroblastoma-derived murine xenograft studies

The preclinical murine xenograft trials were conducted at the Children’s Hospital of Philadelphia (CHOP) and approved by the Institutional Animal Care and Use Committee (IACUC 000643). Fox Chase CB17 SCID mice (CB17/Icr-*Prkdc*^*scid*^/IcrIcoC) were purchased from Charles River Laboratories. The mice were then housed in the CHOP Department of Veterinary Research (DVR) in SuperMouse 750 caged with a 12-h light/dark cycle and a temperature range of 68–74 F and humidity range of 30–70%.

PDX and CDX models are maintained at CHOP and xenograft models were generated as described previously^51,71^. Briefly, viably cryopreserved neuroblastoma PDX tumor fragments or 10 million neuroblastoma cells suspended in Matrigel (Corning Catalog No. 354248) were engrafted subcutaneously into the flanks of mice and passaged once. Twelve mice per model were engrafted to ensure six mice with uniform tumor size of 200 mm^3^ ± 50 mm^3^ were available populate studies. Mice were allocated to either RP-1664 containing chow at 450 ppm (*N* = 4) or standard chow (*N* = 4) stratified by tumor volume at enrollment. One mouse per arm was sacrificed at day 14 and tumor harvested for biomarker analyses (see below). We chose a continuous dosing schedule of RP-1664 for six weeks, followed by two cycles of two weeks on and two weeks off to preliminary study the impact of intermittent dosing. Tumor volumes and weight were measure twice weekly and exposure to RP-1664 was paused if mouse weight decreased by 15% or more. Dosing was re-initiated when body weight recovered to >90%. Study endpoint was tumor volume of 2 cm^3^ or day 70.

### Response evaluation and statistical methods for murine xenograft studies

The six-week time point (end of continuous dosing) was used for all analyses. An event was defined as the quadrupling of a mouse’s tumor volume from day 0 (or baseline measurement). The exact time-to-event (in days) was estimated by interpolating between the measurements directly preceding and following the event, assuming log-linear growth. Differences in event-free survival (EFS) between experimental groups were tested using the Peto and Peto modification of the Gehan-Wilcoxon test (α = 0.05, two-sided alternative).

Initial tumor volume (V0) was measured at initiation of treatment. The mean and standard deviation of V0 was computed within each treatment group, and comparisons between treatment groups were performed using the Wilcoxon rank sum test. At subsequent tumor measurements, the relative tumor volume (RTV) was defined for each mouse as the ratio of its current tumor volume divided by V0. At the conclusion of six weeks, the minimum RTV (minRTV) for each mouse was computed across all measurements except the initial (baseline) one. The mean and standard deviation within each treatment group of minRTV was computed, and comparisons between treatments groups were performed using the Wilcoxon rank sum test.

The objective response measure (ORM) categories are progressive disease (PD, which is subdivided into progressive disease without and with growth delay, PD1 and PD2, respectively, defined only for treated mice), stable disease (SD), partial response (PR), complete response (CR), and maintained complete response (MCR).

ORM categories are defined as:

- PD when <50% tumor regression throughout study and >25% tumor growth at end of study
- PD1 when PD and the mouse’s time-to-event ≤200% the median time-to-event in control group
- PD2 when PD and the mouse’s time-to-event is >200% the median time-to-event in control group
- SD when <50% tumor regression throughout study and ≤25% tumor growth at end of study,
- PR when ≥50% tumor regression at any point during study, but measurable tumor throughout study period
- CR when disappearance of measurable tumor mass during the study period occurs up to two times consecutively or intermittently any number of times
- MCR when no measurable tumor mass for at least three consecutive readings at any time after treatment has been completed

Overall group response is determined by the median response among evaluable mice as follows: Each individual mouse is assigned a score from 0 to 10 based on their ORM: PD1 = 0, PD2 = 2, SD = 4, PR = 6, CR = 8, and MCR = 10, and the median for the group determines the overall response. If the median score is half-way between an ORM number category, the objective response is assigned to the lower response category (e.g., an objective response score of 9 is scored CR). Studies in which toxic deaths are greater than 25% or in which the control group is not SD or worse are considered unevaluable and are excluded from analysis. Treatment groups with PR, CR, or MCR are considered to have had an objective response. Agents inducing objective responses are considered highly active against the tested line, while agents inducing SD or PD2 are considered to have intermediate activity, and agents producing PD1 are considered to have a low level of activity against the tested line.

The average minimum relative tumor volume (minRTV) was also utilized as a tumor volume response measure. A value of 0 indicates that the tumor is no longer detectable, while values < 1.0 indicate some level of tumor regression.

### Efficacy of RP-1664 in Th-MYCN immunocompetent model

Th-MYCN animal experiments were conducted at Children’s Cancer Institute and approved by the University of New South Wales Animal Care and Ethics Committee (ACEC 22/145B) according to the Animal Research Act, 1985 (New South Wales, Australia) and the Australian Code for the Care and Use of Animals for Scientific Purposes (2013). All mice were housed in a specific pathogen-free, Physical Containment level 2 facility, in Ventirack cages (Tecniplast) with the temperature controlled between 22–24 °C and a 12-h day/night. Mice were provided food and water *ad libitum*, with environmental enrichment.

The *Th-MYCN* (Tg(Th-MYCN)41Waw, 129/SvJ*Ter* backcross (ARC, Perth, Australia) mouse model of neuroblastoma overexpresses human *MYCN* in the neuroectodermal cells, and mice develop neuroblastoma as a result^52^. The mice are maintained by breeding hemizygous mice together. All experiments utilized only Th-MYCN^*+/+*^ mice with 8 mice per treatment group and equal numbers of both sexes. Mice were placed on control chow for acclimatization 1–5 days prior to randomization to either control, 225 ppm or 400 ppm RP-1664 chow once tumors reached 5 mm in diameter by abdominal palpation. Body weights were measured daily and calculated based on individual body weight changes relative to the start of treatment. Treatment schedule was 14 days on, 7 days off and mice were euthanized when the tumor reached 10 mm in diameter or when signs of a thoracic tumor manifested. Blood was collected in citrate buffer (3:1) via the saphenous vein from Th-MYCN mice (*N* = 4/arm) on day 5 at 3 timepoints (7:30am, 12:30 pm and 4:30 pm) for PK analysis.

For combination studies, Th-MYCN homozygotes were randomized to treatment groups upon development of a 5 mm diameter tumor. Chemotherapy was given on the first 2 days of treatment i.p. at the following doses: cyclophosphamide (10 mg/kg), topotecan (0.5 mg/kg), irinotecan (2 mg/kg) and temozolomide (5 mg/kg). Anti-GD2 antibody or isotype control were diluted in saline and administered on days 1 and 5, 15 µg per i.p. injection as previously described^86^.

### RP-1664 blood plasma level determination

Micro-sampled whole blood was collected for mouse pharmacokinetic determinations as described^87^. Samples were extracted with 4 volumes of acetonitrile containing an internal standard. The sample extracts were centrifuged, diluted 1:1 with water and quantified against a standard curve using a reversed-phase liquid chromatography gradient coupled to electrospray mass spectrometry operated in positive mode. Whole blood concentrations were converted to plasma by dividing by the mouse blood to plasma ratio of 1.03. The calculated plasma levels were converted to free plasma levels by multiplying by the fraction unbound in mouse plasma f_u_ = 0.125. PK parameters were calculated using non-compartmental analysis using WinNonlin version 8.5.1.3 (Certara, Pennsylvania, USA).

### p21 Immunohistochemistry

All stainings were performed at HistoWiz, Inc. (Brooklyn, NY) using the Leica Bond RX automated stainer (Leica Microsystems), following a standardized operating procedure and fully automated workflow. Samples were processed, embedded in paraffin, and sectioned at 4μm. Slides were dewaxed using xylene and alcohol-based dewaxing solutions. Epitope retrieval was performed via heat-induced epitope retrieval (HIER) using a Tris-based pH 9 solution (Leica Microsystems, AR9640) for 20 minutes at 95 °C. Tissue sections were first incubated with a peroxide block buffer (Leica Microsystems), followed by a 30-minute incubation with rabbit anti-P21 antibody (Abcam, ab109520) at a 1:200 dilution. Detection was performed using DAB rabbit secondary reagents, including polymer, DAB refine, and hematoxylin (Bond Polymer Refine Detection Kit, Leica Microsystems), according to the manufacturer’s protocol. Slides were dried, coverslipped (TissueTek-Prisma Coverslipper), and scanned using a Leica Aperio AT2 slide scanner (Leica Microsystems) at 40x magnification.

### H&E staining and analysis of multipolar mitoses in vivo

For H&E staining slides were stained on a Shandon Gemini automated stainer (ThermoFisher). Briefly, slides were deparaffinized in xylene, rehydrated through a series of descending ethanol concentrations (100% –70%) and rinsed in water. Slides were stained in hematoxylin (Azer Scientific) for 1 min followed by a rinse in water then a quick dip in acid alcohol (0.1%HCl, 50% EtOH). Slides are then rinsed in tap water for 15 min followed by 1 min in Eosin (Azer Scientific). Slides were then dehydrated through a series of ascending ethanol concentrations (70%-100%) followed by xylene before coverslipping with cytoseal (Fisher Scientific). The frequency of multipolar cell division was quantified manually by counting at least 47 cells in metaphase, anaphase or telophase per sample.

### Reporting summary

Further information on research design is available in the Nature Portfolio Reporting Summary linked to this article.

## Supplementary information

Supplementary Information

Description of Additional Supplementary Files

S Data1

S Data2

S Data3

S Data4

S Video 1

S Video 2

S Video 3

S Video 4

S Video 5

Reporting Summary

Transparent Peer Review file

## Source data

Source Data

## Data Availability

The datasets generated and analyzed during the current study are available from the corresponding authors upon request. Previously published whole-genome sequencing, whole-exome sequencing, and RNA-seq data from the Gabriella Miller Kids First cohort are available from dbGaP https://dbgap.ncbi.nlm.nih.gov/beta/study/phs001436.v1.p1/#study, subject to controlled access and data use agreements^76^. Additional de-identified genomic and clinical data from CHOP high-risk neuroblastoma samples are available under an Institutional Review Board–approved protocol and can be shared in aggregate form upon request. The targeted gene panel sequencing data were generated as part of patient care and are available from corresponding author upon request. CRISPR screen results are provided as Supplementary Data. All other data supporting the findings of this study are provided as Source Data within the article and its supplementary information files or are available from the corresponding authors upon reasonable request. Source data are provided with this paper.
