# Supplementary material for: The PLK4 inhibitor RP-1664 demonstrates potent efficacy in neuroblastoma preclinical models through a dual mechanism of sensitivity
Source: Nat Commun. 2026 Jun 13;17:7531. doi: 10.1038/s41467-026-74061-5 (PMC13408883; doi:10.1038/s41467-026-74061-5)
Supplement: Supplementary file 1 — Supplementary Information [file 41467_2026_74061_MOESM1_ESM.pdf]

SUPPLEMENTARY INFORMATION

Supplementary Figure 1.

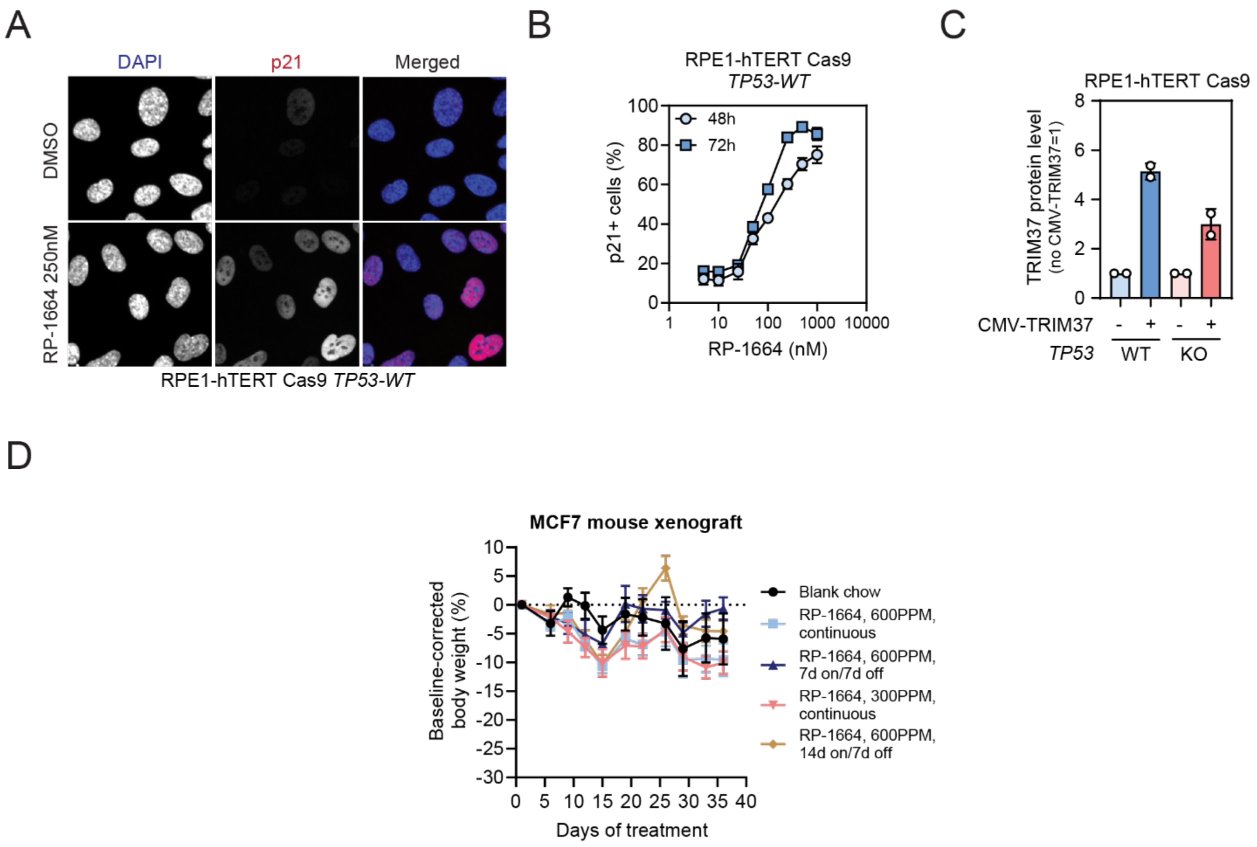

Supplementary Figure 1. Related to Figure 1. A. Representative micrographs of RPE1-hTERT Cas9 cells after no treatment or treatment with RP-1664 and p21 immunostaining. DAPI is a nuclear counterstain. B. Quantification of p21-positive (p21+) RPE1-hTERT Cas9 cells after 48 or 72h treatment with indicated concentrations of RP-1664. Mean of *N*=3 independent experiments ±SD. C. Quantification of TRIM37 protein levels in RPE1 TP53-WT and KO cells with or without CMV-TRIM37 overexpression. Measured by capillary immunodetection, data from *N*=2 independent experiments (circles) with mean (bars) ±SD. D. Baseline-corrected body weight change in mice bearing MCF7 xenograft tumors upon indicated treatments and schedules. Mean of *N*=6 mice/group ±SEM. Source data: SourceData_SuppFigure1.xlsx.

Supplementary Figure 2.

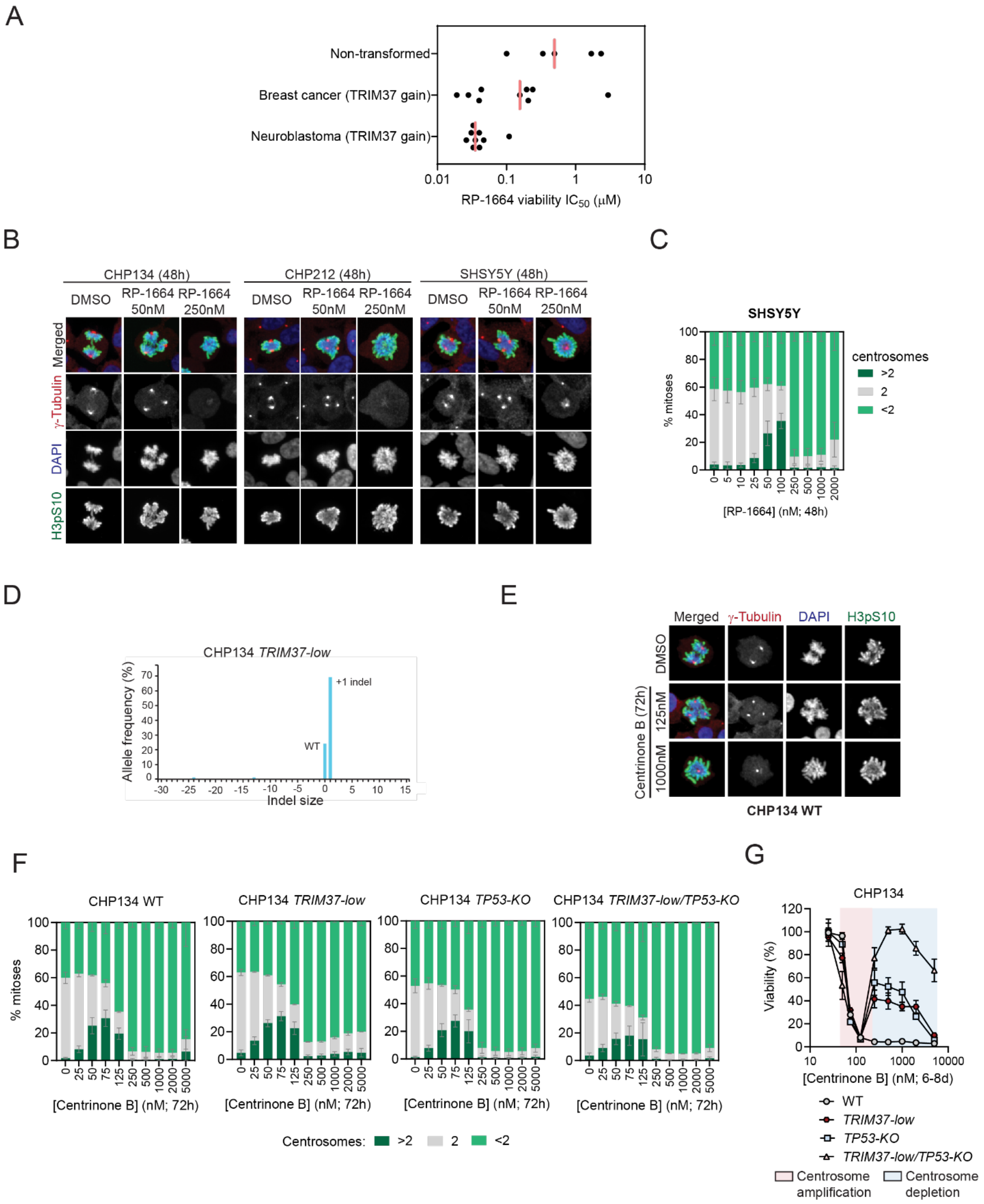

Supplementary Figure 2. Related to Figure 3. A. RP-1664 IC_{50} values from Incucyte or CellTiterGlo cell growth assays in a panel of non-transformed cell lines (RPE1, MCF10A, COL-hTERT, BRONCH2, HK2) and a panel of breast cancer cell lines carrying extra copies of *TRIM37* (Supplementary Table 1), as compared to neuroblastoma cell line IC_{50} values (Figure 3A). Data from non-transformed cell lines originates from *N*=3 independent experiments. Breast cancer data were derived from *N*=3 technical replicates per cell line B. Representative micrographs of CHP134, CHP212 and SHSY5Y cells after no treatment of treatment with indicated RP-1664 concentrations and immunofluorescence staining with γ-Tubulin (visualizing centrosomes) and H3-pS10 (mitotic marker) antibodies. DAPI is a nuclear counterstain. C. Quantification of mitotic SHSY5Y cells with <2, 2, and >2 centrosomes at indicated RP-1664 concentrations in *N*=3 independent experiments. Mean value (bars) is shown ±SD. D. ICE[84] quantification of indel allele frequency in CHP134 *TRIM37-low* cells. E. Representative micrographs of CHP134, CHP212 and SHSY5Y cells after no treatment of treatment with indicated centrinone B concentrations and immunofluorescence staining with γ-Tubulin (visualizing centrosomes) and H3-pS10 (mitotic marker) antibodies. DAPI is a nuclear counterstain. F. Quantification of mitotic CHP134 WT, *TRIM37-low*, *TP53-KO* and *TRIM37-low/TP53-KO* cells with <2, 2, and >2 centrosomes at indicated centrinone B concentrations in *N*=2-3 independent experiments. Mean value (bars) is shown ±SD. G. Viability of CHP134 cells of indicated genotypes upon treatment with indicated concentrations of centrinone B as measured by Incucyte growth assays. Concentrations inducing centrosome amplification (pink) and depletion (blue) are highlighted. Mean of *N*=3 independent experiments ±SD. Source data: SourceData_SuppFigure2.xlsx.

Supplementary Figure 3.

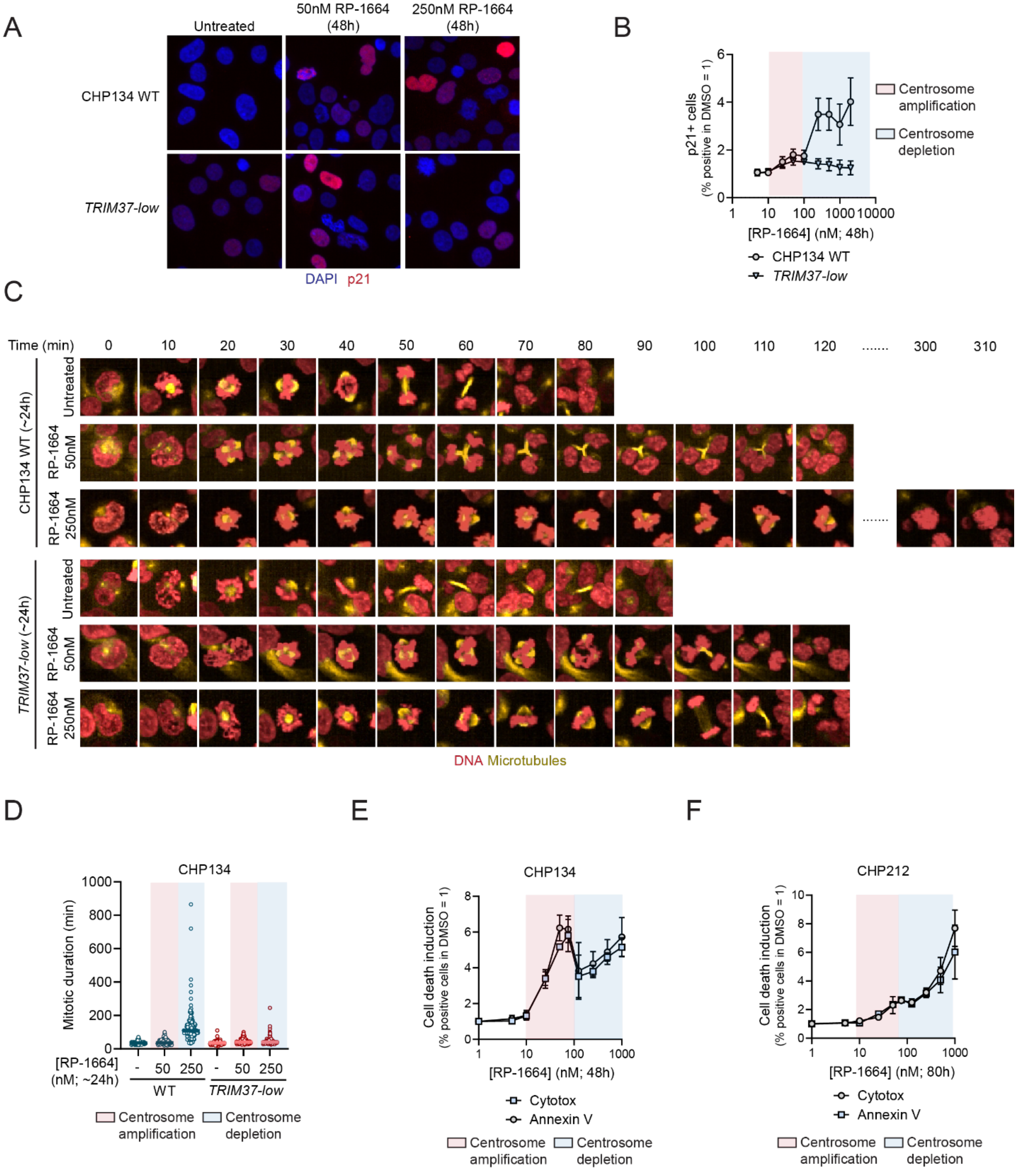

Supplementary Figure 3. Related to Figure 3. A. Representative micrographs of CHP134 WT and *TRIM37-low* cells after no treatment or treatment with RP-1664 and p21 immunostaining. DAPI is a nuclear counterstain. B. Quantification of p21-positive (p21+) CHP134 WT and *TRIM37-low* cells after 48h treatment with indicated concentrations of RP-1664. Mean of *N*=3 independent experiments ±SD. Concentrations inducing centrosome amplification (pink) and depletion (blue) are highlighted. C. Representative tempograms from time-lapse imaging of CHP134 WT and *TRIM37-low* cells stained with SPY555-Tubulin for microtubules (yellow) and SPY650-DNA for DNA (red) with or without treatment with indicated RP-1664 concentrations. D. Quantification of mitotic length in CHP134 WT and *TRIM37-low* cells by live-cell imaging. Cells were treated with DMSO, 50nM RP-1664 (centrosome amplification) or 250nM RP-1664 (centrosome depletion). Data from *N*=2 independent experiments. Each data point is a single cell with median (solid line). E,F. Induction of cell death by RP-1664 in CHP134 (E) and CHP212 (F) neuroblastoma cells. Fold change from DMSO in the percent of Cytotox- and Annexin V-positive cells after indicated RP-1664 concentrations. Mean of N=3 independent experiments ±SD. Concentrations of RP-1664 inducing centrosome amplification (pink) and depletion (blue) are highlighted. Source data: SourceData_SuppFigure3.xlsx.

Supplementary Figure 4.

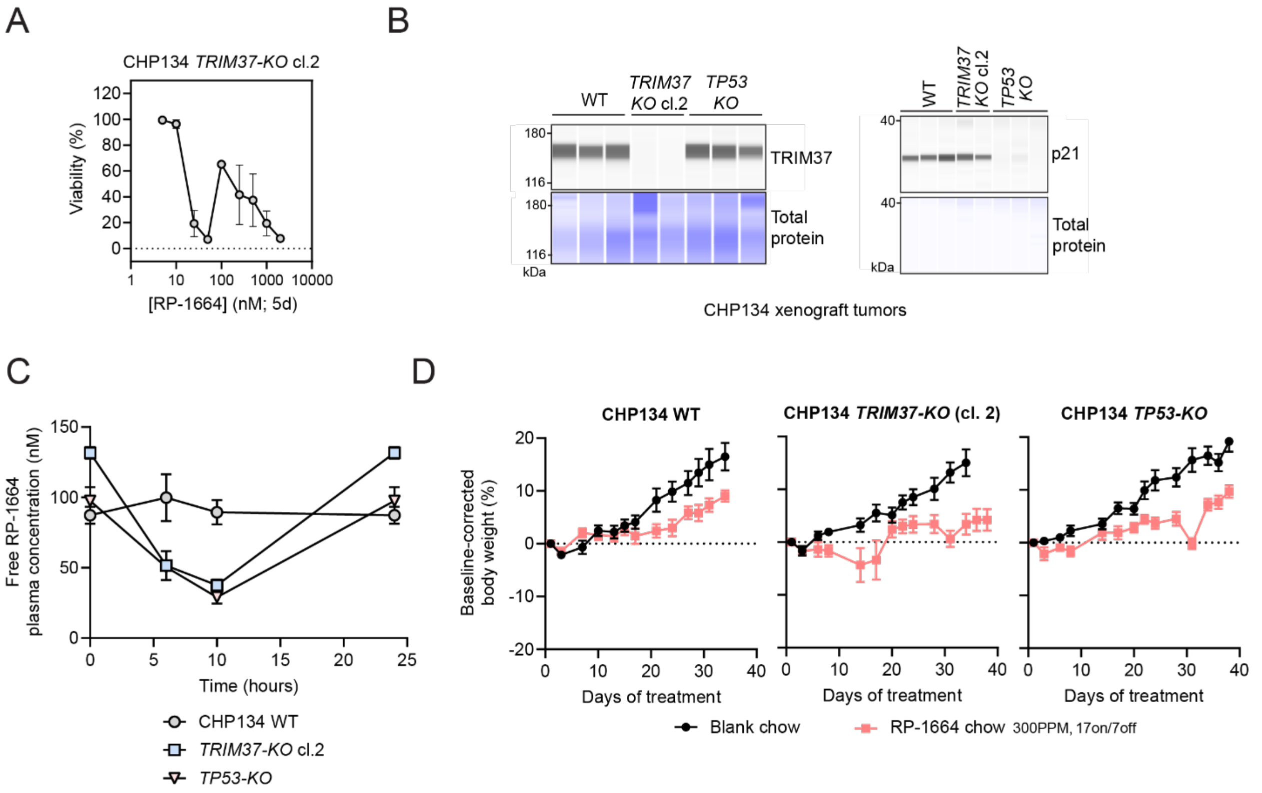

Supplementary Figure 4. Related to Figure 3. A. RP-1664 sensitivity of CHP134 *TRIM37-KO* clone 2 cells used as a xenograft model. Mean cell viability values from *N*=3 Incucyte growth assays ±SD. B. TRIM37 (left) and p21 (right) capillary immunodetection in tumor lysates of CHP134 xenografts of indicated genotypes. Each lane represents an individual tumor. Total protein is a loading control. C. Free (not bound to plasma protein) plasma concentrations of RP-1664 in mice bearing CHP134 tumors of indicated genotypes treated with 300ppm RP-1664 chow. Mean of *N*=3 mice at indicated time points post dosing is shown ±SEM. D. Baseline-corrected body weight change in mice bearing CHP134 WT, *TRIM37-KO* and *TP53-KO* xenograft tumors upon indicated treatments. Values in A,B are mean of 7 mice/group ±SEM. Source data: SourceData_SuppFigure4.xlsx.

Supplementary Figure 5.

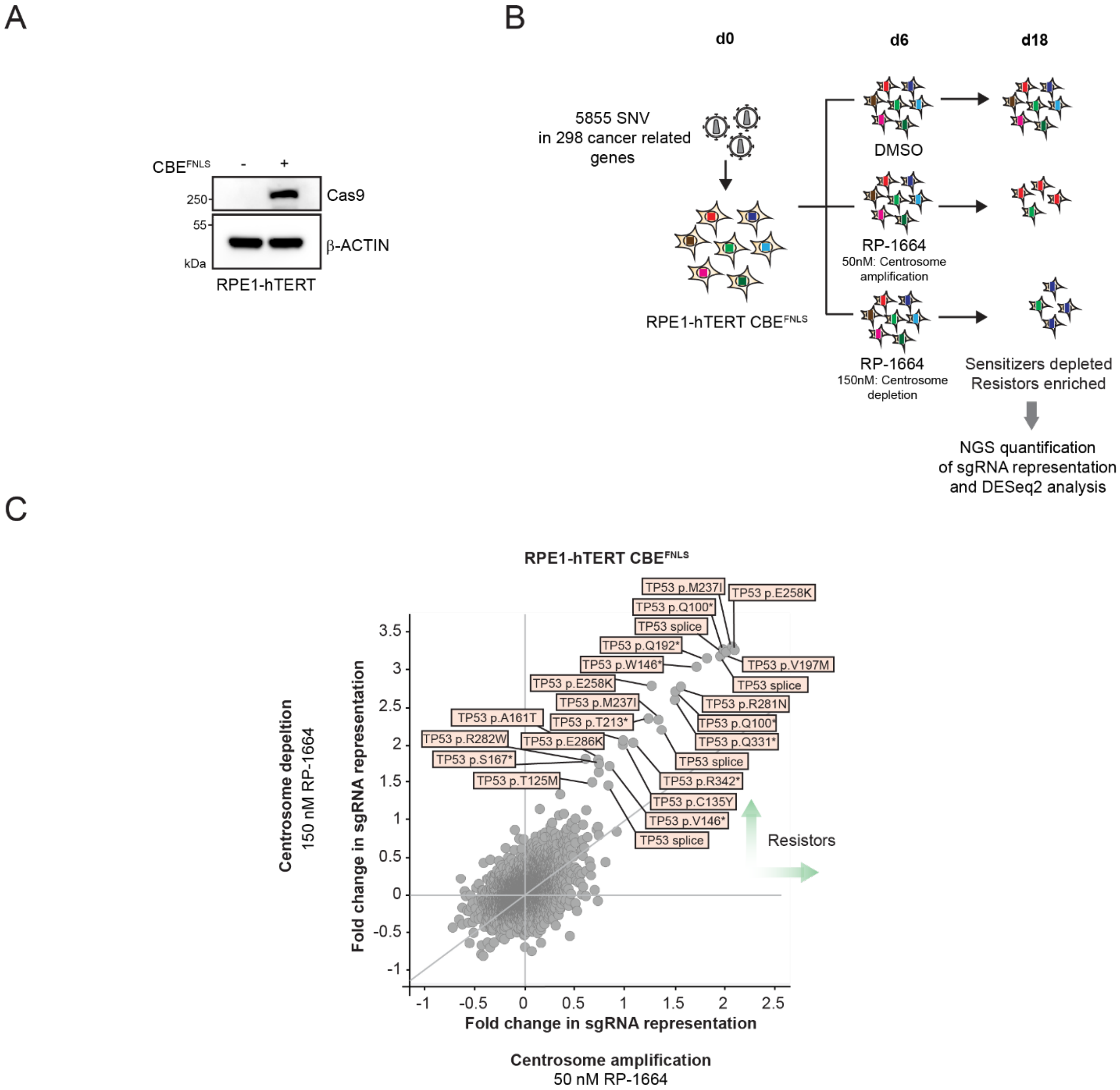

Supplementary Figure 5. Related to Figure 4. A. Expression of the CBE^{FNLS} base editor in RPE1-hTERT cells. Representative anti-spCas9 immunoblot of whole cell extracts. β-ACTIN is a loading control. B. Experimental design of a base-editing CRISPR screen for cancer-relevant single-nucleotide variants (SNVs) that modulate RP-1664 sensitivity in RPE1-hTERT CBE^{FNLS} cells. See Methods for details. C. Screen results. Fold changes in representation of individual sgRNAs in cells treated with 50nM RP-1664 (X axis) vs. 150nM (Y axis). sgRNAs inducing *TP53* mutations that cause RP-1664 resistance are highlighted. Source data: Supplementary Data 3.

Supplementary Figure 6.

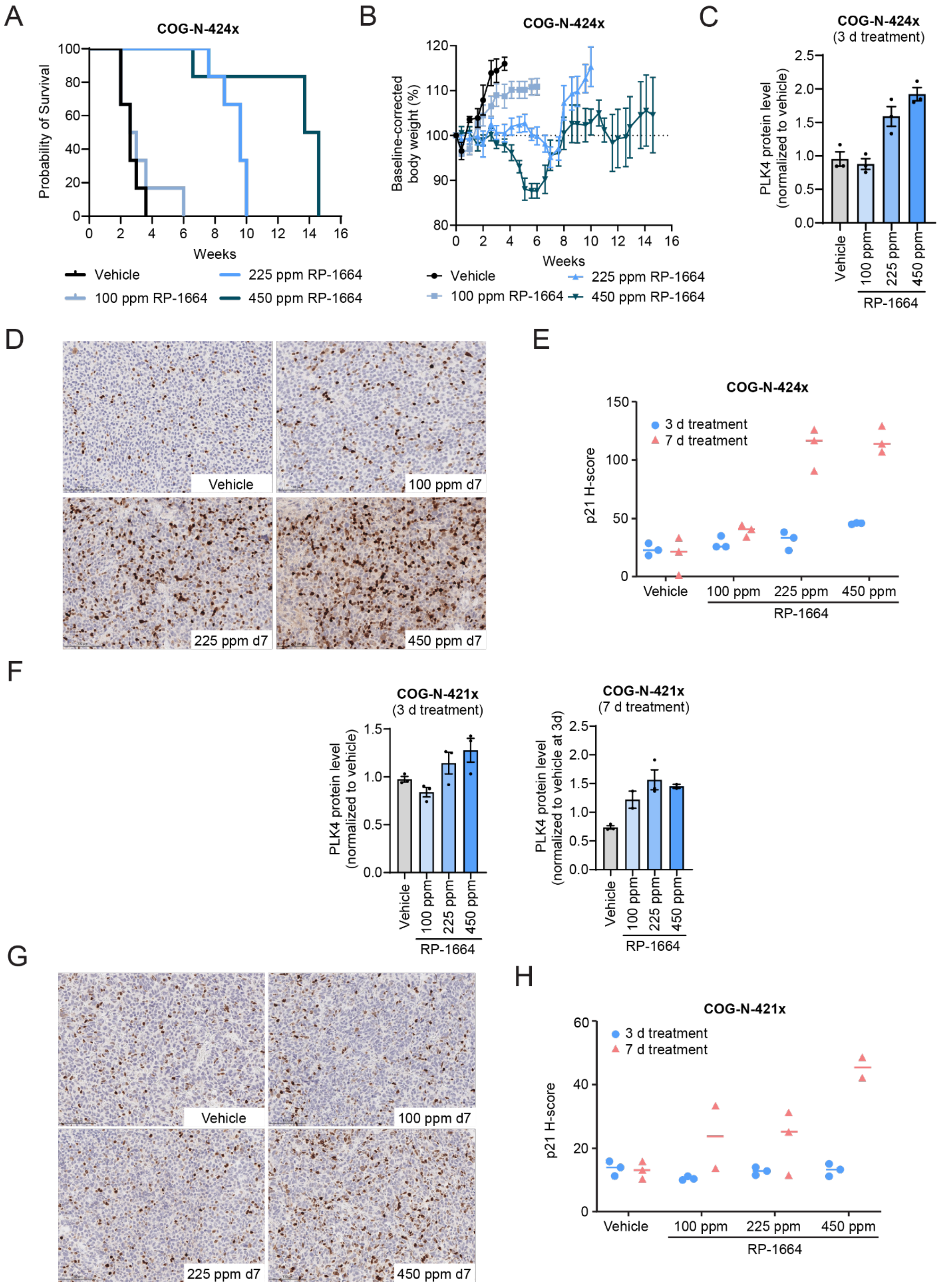

Supplementary Figure 6. Related to Figure 6. A. Kaplan-Meier plot showing survival of mice bearing COG-N-424x tumors treated with blank chow or indicated doses of RP-1664 chow. B. Body weight changes in COG-N-424x tumor-bearing mice upon indicated treatments. Mean of N=6 mice/group ±SEM. C. PLK4 protein level quantification by capillary immunodetection in lysates from COG-N-424x tumors treated for 3 days with indicated doses of RP-1664. Mean of *N*=3 mice ±SEM. D. Representative micrographs of formalin-fixed paraffin-embedded (FFPE) COG-N-424x xenograft tumor sections processed for immunohistochemistry (IHC) with a p21 antibody upon indicated treatments. E. p21 IHC quantification (H-score) in COG-N-424x tumors upon indicated treatments at indicated time points. Symbols represent individual mice (*N*=3/group), bars show mean. F. PLK4 protein level quantification by capillary immunodetection in lysates from COG-N-421x tumors treated for 3 (left) or 7 (right) days with indicated doses of RP-1664. Mean of *N*=2-3 mice ±SEM. G,H. Representative micrographs (G) and p21 H-score quantification (H) in COG-N-421x tumors upon indicated treatments at indicated time points. Symbols represent individual mice (*N*=2-3/group), bars show mean. I. Example images of multipolar mitoses in RP-1664-treated COG-N-421x mouse xenografts stained with H&E. J. Quantification of multipolar mitoses in vehicle- or RP-1664-treated COG-N-421x tumors. Each bar represents one animal, *N*=3 mice/group. Source data: SourceData_SuppFigure6.xlsx.

Supplementary Figure 7.

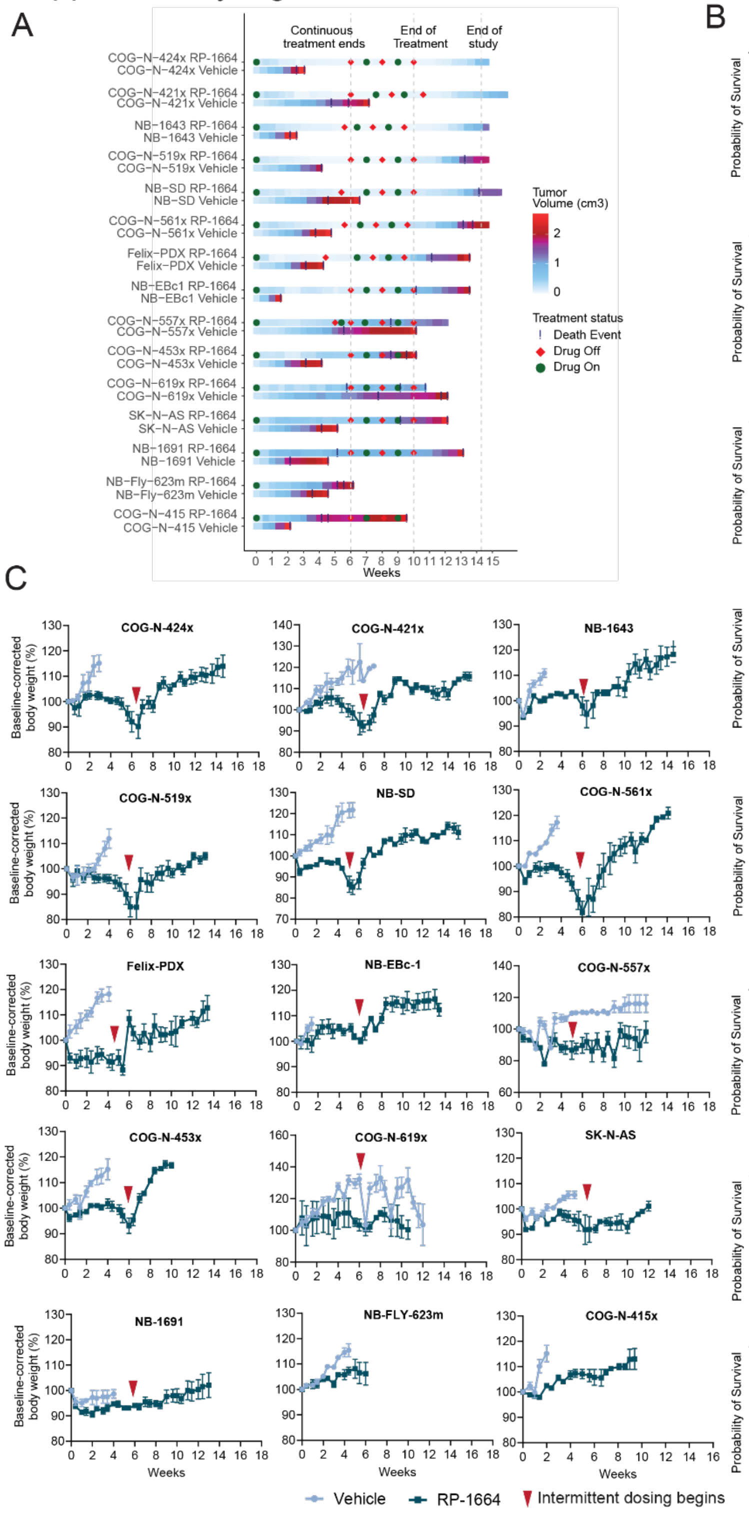

Supplementary Figure 7. Related to Figure 6. A. Swimmer plots of vehicle- and RP-1664-treated neuroblastoma xenograft tumor volumes over time. Color indicates mean tumor volume (N=3 mice/group). Treatment periods and death events are indicated. B. Kaplan-Meier plots showing survival of mice bearing indicated xenograft tumors treated with blank chow or RP-1664 chow. C. Relative body weight changes in mice bearing indicated neuroblastoma xenografts treated with vehicle or RP-1664 chow. Mean of N=3 mice/group. SourceData_SuppFigure7.xlsx

Supplementary Figure 8.

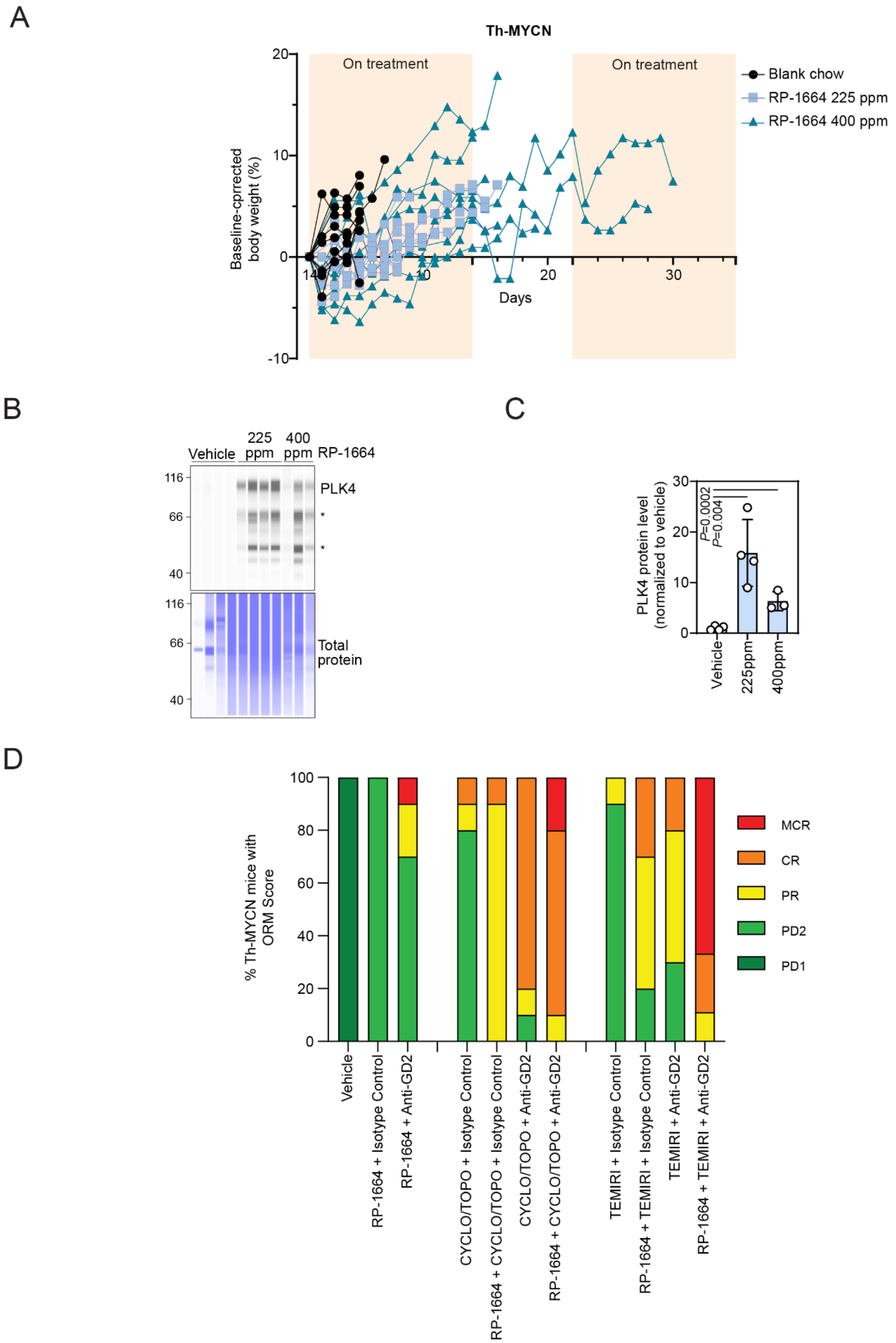

Supplementary Figure 8. Related to Figure 7. A. Percentage weight loss of Th-MYCN tumor-bearing mice treated with blank chow or RP-1664 over time. Data show individual mice. B. SimpleWestern immunocapillary detection of PLK4 in blank chow (vehicle)- or RP-1664-treated Th-MYCN mouse tumor lysates. Total protein was used as a loading control. Asterisks indicate degradation products of PLK4. C. Quantification of PLK4 protein level in Th-MYCN tumors after indicated treatments from experiment shown in B. Data from individual mice (circles, *N*=3-4 mice/group) with mean (bar) ±SD. D. Distribution of objective response measures (ORM) of Th-MYCN mouse tumors upon indicated treatments. *N*=10 mice/group. SourceData_SuppFigure8.xlsx

Supplementary Table 1. Related to Supplementary Figure 2A. *TRIM37* copy number in breast cancer cell lines from Supplementary Figure 2A. Data derived from DepMap.

**Cell line**	**Extra *TRIM37* copies**<br>**(***TRIM37* copy number – ploidy)
HCC1500	1
CAMA1	1
DU4475	2
ZR751	3
EFM192A	4
HCC1428	4
ZR7530	4
MX1	5
MCF7	30

Supplementary Table 2. Related to Figure 6 and Supplementary Figures 6,7. Characteristics of 15 models used in xenograft panel. PLK4 and TRIM37 expression level, TRIM37 copy number, and genotypes for selected driver alterations. FPKM=Fragments Per Kilobase of transcript per Million mapped reads WT=wildtype.

MODEL	MYCN Amplification Status	PLK4 FPKM	TRIM37 FPKM	chr17q CN	TRIM37 CN	ALK	HRAS	KRAS	NRAS	PTPN11	TP53	BRAF
COG-N-424x	Amplified	16.3	27.5	gain	gain	WT	WT	WT	WT	WT	WT	V600E
COG-N-421x	Amplified	26.3	38.4	gain	gain	WT	WT	WT	WT	WT	WT	WT
NB-1643	Amplified	17.9	35.1	gain	gain	R1275Q	WT	WT	WT	WT	WT	WT
COG-N-519x	Amplified	10.5	14.3	gain	gain	WT	WT	WT	WT	WT	G245S	WT
NB-SD	Amplified	23.5	29.0	gain	gain	F1174L	WT	WT	WT	WT	C176F	WT
COG-N-561x	Amplified	15.8	27.1	gain	gain	F1245I	WT	WT	WT	WT	WT	WT
Felix-PDX	Non-Amplified	23.5	44.6	gain	gain	F1245C	WT	WT	WT	WT	WT	WT
NB-EBc1	Amplified	19.7	28.3	gain	gain	WT	WT	G12D	WT	WT	WT	WT
COG-N-557x	Amplified	22.6	33.3	gain	gain	F1245L	WT	WT	WT	WT	WT	WT
COG-N-453x	Amplified	21.5	38.7	gain	gain	F1174L	WT	WT	WT	WT	WT	WT
COG-N-619x	Non-Amplified	3.5	8.3	gain	gain	WT	WT	WT	WT	G503V	WT	WT
SK-N-AS	Non-Amplified	8.8	13.7	gain	gain	WT	WT	WT	Q61K	WT	H168R	WT
NB-1691	Amplified	19.2	34.0	gain	gain	WT	WT	WT	WT	E69K	WT	WT
NB-Fly-623m	Non-Amplified	21.9	38.1	gain	gain	R1275Q	WT	WT	WT	WT	WT	WT
COG-N-415x	Amplified	16.3	25.3	gain	gain	F1174L	WT	WT	WT	WT	WT	WT

Supplementary Table 3. Efficacy of RP-1664 with chemoimmunotherapy in Th-MYCN mice. Related to Figure 7 and Supplementary Figure 8D.

Treatment Group	Animal Counts/Status						EFS Evaluation					ORM	
	N[1]	Nd[2]	Nx[3]	Na[4]	Nev[5]	Nt[6]	KM med[7]	EFS T - C[8]	EFS T/C[9]	P-value[10]	P-value[11]	Median ORM score	Heat Map
Vehicle	10	0	0	10	10	0	3.5					PD1	
RP-1664 + Isotype	10	0	0	10	10	0	15	12.5	4.3	<0.0001		PD2	
RP-1664 + Anti-GD2	10	0	0	10	7	2	18	14.5	5.1	<0.0001	0.0454	PD2	
CYCLO/TOPO + Isotype	10	0	0	10	10	0	11	8.5	3.1	<0.0001		PD2	
CYCLO/TOPO + Isotype + RP-1664	10	0	0	10	10	0	27.5	24	7.9	<0.0001	<0.0001	PR	
CYCLO/TOPO + Anti-GD2	10	0	0	10	4	6	50.5	47	14.4	<0.0001		CR	
CYCLO/TOPO + Anti-GD2 + RP-1664	10	0	0	10	5	3	78	74.5	22.3	<0.0001	0.0215	CR	
TEMIRI + Isotype	10	0	0	10	10	0	13	9.5	3.7	<0.0001		PD2	
TEMIRI + Isotype + RP-1664	10	0	0	10	9	1	22	18.5	6.3	<0.0001	<0.0001	PR	
TEMIRI + Anti-GD2	10	0	0	10	7	3	26	22.5	7.4	<0.0001		PR	
TEMIRI + Anti-GD2 + RP-1664	10	0	1	9	2	1	Undefined			<0.0001	<0.0001	MCR	

[1] N = total number of mice entering experiment

[2] Nd = number of mice euthanized before endpoint was reached due to >20% weight loss or other treatment-related health effects

[3] Nx = number of additional mice excluded from analysis

[4] Na = number of mice in analysis

[5] Nev = number of events, defined as tumours reaching 10mm

[6] Nt = number of events, defined as thoracic tumour

[7] KM med = Kaplan-Meier estimate of median time-to-event (days)

[8] EFS T – C = Tumour Growth Delay (LGD) = difference in median time-to-event (days) between T and C groups

[9] EFS T/ C = relative difference in median time-to-event (days) between T and C groups

[10] P-value comparing EFS (treatment group) to EFS (vehicle-treated group), computed using Log-rank testing

[11] P-value comparing EFS (RP-1664 treatment group) to EFS (relevant control group), computed using Log-rank testing

Uncropped Blot Images for Supplementary Figures

Supplementary Figure 4B.

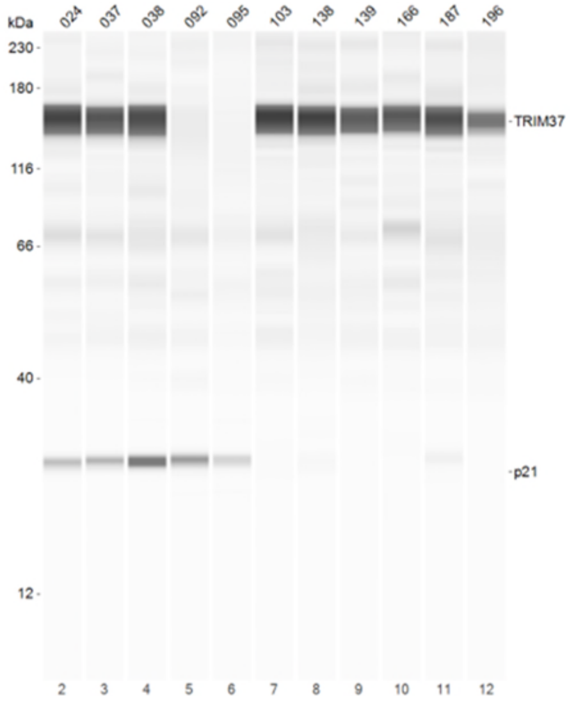

WT TRIM37 KO TP53 KO

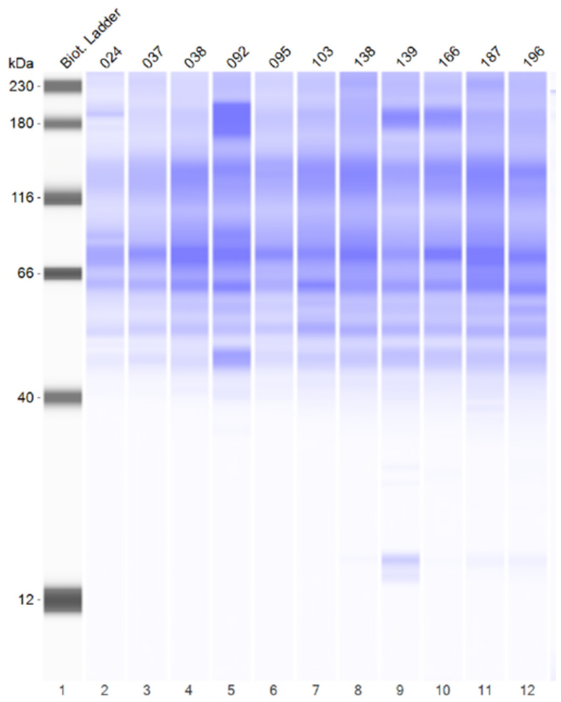

Total Protein

Supplementary Figure 5A.

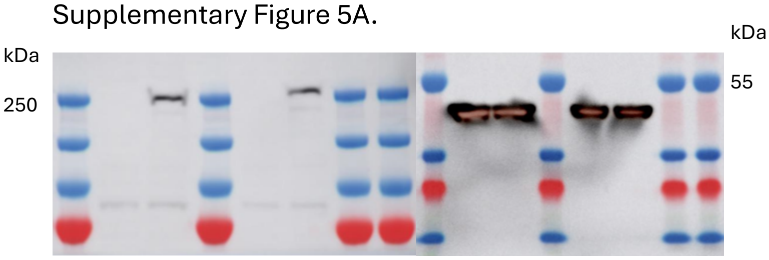

Cas9 β-Actin

Supplementary Figure 8B.

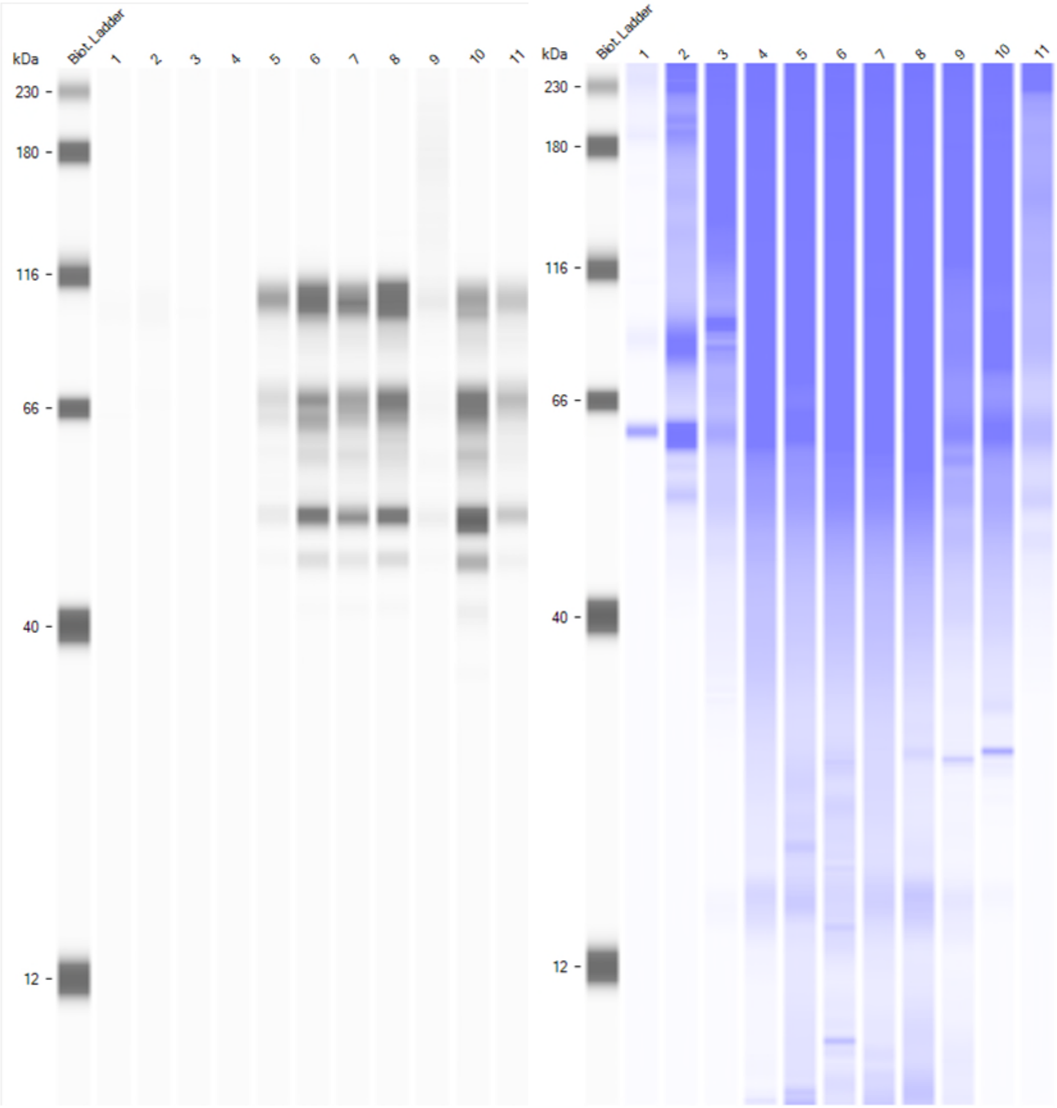

PLK4 Total Protein
