# Supplementary material for: The PLK4 inhibitor RP-1664 demonstrates potent efficacy in neuroblastoma preclinical models through a dual mechanism of sensitivity
Source: Nat Commun. 2026 Jun 13;17:7531. doi: 10.1038/s41467-026-74061-5 (PMC13408883; doi:10.1038/s41467-026-74061-5)
Supplement: Supplementary file 2 — Description of Additional Supplementary Files [file 41467_2026_74061_MOESM2_ESM.pdf]

Title: Supplementary Data 1

Description: Related to Figure 4B. CHP134 TRIM37-low TP53 KO CRISPR screen results. Median sgRNA fold-changes per gene (enrichment over initial time point) at indicated timepoints in DMSO- or RP 1664-treated cells.

Title: Supplementary Data 2

Description: Related to Figure 4D. RPE1 CRISPR screen results. Gene-level DrugZ data. Columns labeled 'C' refer to 50nM RP-1664 treatment (centrosome amplification) and columns labeled 'D' refer to 150 nM (centrosome loss).

Title: Supplementary Data 3

Description: Related to Supplementary Figure 4D. RPE1 base editing screen results. Target mutations, fold-changes (RP 1664 over DMSO at final time point) and P-values for individual sgRNAs in the 50nM RP-1664 (centrosome amplification) and 150nM (centrosome loss) treatment arms.

Title: Supplementary Data 4

Description: Efficacy of RP-1664 in neuroblastoma xenograft models. Details of statistical analysis used to evaluate time-to-event outcomes following treatment with RP 1664 versus control in neuroblastoma xenograft models. KM Med: Kaplan Meier estimate of median event-free survival (EFS). EFS T – C: Absolute difference in median EFS between treatment and control groups. EFS T/C: ratio of median EFS in the treatment group to control. P value from the Gehan Wilcoxon test: statistical significance of differences in EFS. minRTV: Average minimum relative tumor volume observed in each group ±SD. Med resp: Objective response measures. CR = complete response, MCR = maintained CR, and PD/PD1/PD2 = progressive disease. See Methods for more information.

Title: Supplementary Video 1,2

Description: Related to Figure 4A. Example time-lapse video of CHP134 cells with (Supplementary Video 2) or without (Supplementary Video 1) RP-1664 treatment. Microtubules are labeled in yellow, DNA in red.

Title: Supplementary Video 3-5

Description: Related to Figure 4A. Example time-lapse video of RPE1-hTERT Cas9 TP53 WT cells with (Supplementary Video 4,5) or without (Supplementary Video 3) RP-1664 treatment. Microtubules are labeled in yellow, DNA in red.
