# Supplementary material for: The PLK4 inhibitor RP-1664 demonstrates potent efficacy in neuroblastoma preclinical models through a dual mechanism of sensitivity
Source: Nat Commun. 2026 Jun 13;17:7531. doi: 10.1038/s41467-026-74061-5 (PMC13408883; doi:10.1038/s41467-026-74061-5)
Supplement: Supplementary file 12 — Reporting Summary [file 41467_2026_74061_MOESM12_ESM.pdf]

nature portfolio

Corresponding author(s): Michal Zimmermann
John M. Maris

Last updated by author(s): Feb 19, 2026

Reporting Summary

Nature Portfolio wishes to improve the reproducibility of the work that we publish. This form provides structure for consistency and transparency in reporting. For further information on Nature Portfolio policies, see our Editorial Policies and the Editorial Policy Checklist.

Statistics

For all statistical analyses, confirm that the following items are present in the figure legend, table legend, main text, or Methods section.

n/a	Confirmed	
☐	☒	The exact sample size (n) for each experimental group/condition, given as a discrete number and unit of measurement
☐	☒	A statement on whether measurements were taken from distinct samples or whether the same sample was measured repeatedly
☐	☒	The statistical test(s) used AND whether they are one- or two-sided<br>*Only common tests should be described solely by name; describe more complex techniques in the Methods section.*
☐	☒	A description of all covariates tested
☐	☒	A description of any assumptions or corrections, such as tests of normality and adjustment for multiple comparisons
☐	☒	A full description of the statistical parameters including central tendency (e.g. means) or other basic estimates (e.g. regression coefficient) AND variation (e.g. standard deviation) or associated estimates of uncertainty (e.g. confidence intervals)
☐	☒	For null hypothesis testing, the test statistic (e.g. F, t, r) with confidence intervals, effect sizes, degrees of freedom and P value noted<br>*Give P values as exact values whenever suitable.*
☒	☐	For Bayesian analysis, information on the choice of priors and Markov chain Monte Carlo settings
☒	☐	For hierarchical and complex designs, identification of the appropriate level for tests and full reporting of outcomes
☐	☒	Estimates of effect sizes (e.g. Cohen's d, Pearson's r), indicating how they were calculated

Our web collection on statistics for biologists contains articles on many of the points above.

Software and code

Policy information about availability of computer code

Data collection	Compass for Simple Western, v 6.3 (Bio-Techne)<br>Harmony v 5.3 (Revvity)<br>Incucyte 2022B Rev2 (Sartorius)

Data analysis	PRISM v8-v10 (GraphPad)<br>Compass for Simple Western, v 6.3 (Bio-Techne)<br>Harmony v 5.3 (Revvity)<br>Incucyte 2022B Rev2 (Sartorius)<br>HALO (Indica Labs)<br>DrugZ (reference #85)<br>DESeq2 (https://bioconductor.org/packages/devel/bioc/html/DESeq2.html)<br>NGSCheckMate<br>Control-FREEC<br>CNVkit<br>GATK<br>STAR<br>RSEM<br>Gencode release v39

For manuscripts utilizing custom algorithms or software that are central to the research but not yet described in published literature, software must be made available to editors and reviewers. We strongly encourage code deposition in a community repository (e.g. GitHub). See the Nature Portfolio guidelines for submitting code & software for further information.

Data

Policy information about availability of data

All manuscripts must include a data availability statement. This statement should provide the following information, where applicable:

- Accession codes, unique identifiers, or web links for publicly available datasets
- A description of any restrictions on data availability
- For clinical datasets or third party data, please ensure that the statement adheres to our policy

CRISPR screen results are included as Supplementary Tables. Additional data will be available from the corresponding authors upon reasonable request.

Research involving human participants, their data, or biological material

Policy information about studies with human participants or human data. See also policy information about sex, gender (identity/presentation), and sexual orientation and race, ethnicity and racism.

Reporting on sex and gender	The whole genome sequencing data (WGS) utilized in this project was obtained through the Gabriella Miller Kids First Project (dbGaP phs001436.v1.p1). The cohort represent the expected distribution of male and female subjects.
Reporting on race, ethnicity, or other socially relevant groupings	The cohort represent the expected distribution of race and ethnicity in this rare cancer population.
Population characteristics	See above.
Recruitment	Sample from the genomic analyses were accessioned through the Children's Oncology Group Biobank after informed consent for tissue banking. All samples are deidentified. For the validation of 17q gain, we retrospectively identified high-risk neuroblastoma patients who underwent tumor sequencing as part of routine clinical care.
Ethics oversight	Validation of 17q copy number gain status in CHOP tumor samples was undertaken under 501 Children's Hospital of Philadelphia Institutional Review Board (IRB) protocol 25-023583.

Note that full information on the approval of the study protocol must also be provided in the manuscript.

Field-specific reporting

Please select the one below that is the best fit for your research. If you are not sure, read the appropriate sections before making your selection.

[x] Life sciences [ ] Behavioural & social sciences [ ] Ecological, evolutionary & environmental sciences

For a reference copy of the document with all sections, see nature.com/documents/nr-reporting-summary-flat.pdf

Life sciences study design

All studies must disclose on these points even when the disclosure is negative.

Sample size	No prospective sample size calculation was performed. Sample sizes were chosen based on standard values in the field (N>=3).
Data exclusions	No data points were excluded from analyses.
Replication	For all cell biology experiments, multiple independent experimental replicates or were performed, as indicated in respective Figures. Results were successfully reproduced in all cases. CRISPR screens were performed in technical replicates, as is the standard in the field. These replicates showed high correlation, confirming reproducibility.

	Mouse xenograft experiments were performed in N>3 mice per cohort.
Randomization	Samples in cell biology experiments were not randomized. In mouse xenograft experiments, tumor bearing mice were randomized into treatment cohorts.
Blinding	Investigators were not blinded for data analysis.

Reporting for specific materials, systems and methods

We require information from authors about some types of materials, experimental systems and methods used in many studies. Here, indicate whether each material, system or method listed is relevant to your study. If you are not sure if a list item applies to your research, read the appropriate section before selecting a response.

Materials & experimental systems

n/a	Involved in the study
☐	☒ Antibodies
☐	☒ Eukaryotic cell lines
☒	☐ Palaeontology and archaeology
☐	☒ Animals and other organisms
☒	☐ Clinical data
☒	☐ Dual use research of concern
☒	☐ Plants

Methods

n/a	Involved in the study
☒	☐ ChIP-seq
☒	☐ Flow cytometry
☒	☐ MRI-based neuroimaging

Antibodies

Antibodies used	The following antibodies and dilutions were used for immunoblotting (IB), immunofluorescence(IF) or capillary immunodetection (JESS): rabbit anti-PLK4 E6A7R (Cell Signaling Technologies71033; JESS 1:200), rabbit anti-TRIM37 (Bethyl A301-174A; JESS 1:50), mouse anti-p53 DO-1(sc-126; IB 1:1000), rabbit anti-p21 12D1 (Cell Signaling Technologies 2947; IF 1:500, JESS1:300), rabbit anti-g-Tubulin EPR16793 (Abcam 179503; IF 1:500), mouse anti-H3pS10 3H10(Sigma Aldrich 05-806; IF 1:1000), rabbit anti-KIFC1 (ProteinTech 20790-1-AP; JESS 1:50), rabbitanti-DYKDDDDK tag (FLAG) D6W5B (Cell Signaling Technologies 14793; IB 1:1000), rabbit anti-Vinculin E1E9V (Cell Signaling Technologies 13901; IB 1:5000), Alexa Fluor 488/555/647-conjugated goat anti-rabbit or antimouse<br>IgG (H+L) (Thermo Fisher Scientific A-11008/A-21428/A-21245/A-11001/A-21422/A-21235; IF 1:500-1:1000), HRP-conjugated goat anti-mouseIgG (BioRad L005680; 1:5000 IB), HRP-conjugated goat anti-rabbit IgG (Jackson ImmunoResearch 111-035-144; IB 1:5000), HRP-conjugated anti-rabbit secondary antibody (Bio-Techne 042-206; JESS undiluted).
Validation	PLK4, p53, p21, TRIM37 and KIFC1 antibodies were validated using target knockout/knockdown or overexpression, as indicated in respective Figures. Validation of all other antibodies was provided by respective vendors and is available on the vendors' websites.

Eukaryotic cell lines

Policy information about cell lines and Sex and Gender in Research

Cell line source(s)	Cell lines were purchased from the following vendors: RPE1, MCF7, CHP212, SHSY5Y, SKNAS, SKNDZ, SKNFI, SKNSH, HK2 - ATCC; KELLY, MCF10A – Sigma Aldrich; CHP134, IMR32 – DSMZ, COL-hTERT, BRONCH2 – Applied Biological Materials. RPE1-hTERT Cas9 TP53-WT and TP53-KO were described in references 34 and 75. Breast cancer cell line panel in Extended Data Figure 2A was maintained by Crown Bio, Inc.
Authentication	Cell line authentication was provided by the respective vendors.
Mycoplasma contamination	All cell lines tested negative for mycoplasma.
Commonly misidentified lines<br>(See ICLAC register)	None commonly misidentified cell lines were used.

Animals and other research organisms

Policy information about studies involving animals; ARRIVE guidelines recommended for reporting animal research, and Sex and Gender in Research

Laboratory animals	MCF7 xenografts were engrafted into female, 6-8 week old BALB/c Nude mice (Charles River Laboratories) .<br>CHP134 WT, TRIM37-KO and TP53-KO xenografts were engrafted into female, 6-8 week old CB17 SCID mice (Charles River Laboratories).<br>Human neuroblastoma-derived xenograft models were engrafted into 6–8-week-old female Fox Chase CB17 SCID mice (CB17/Icr-Prkdcscid/IcrIcoC) purchased from Charles River Laboratories<br>Th-MYCN transgenic mice (Tg(Th-MYCN)41Waw, 129/SvJTer backcross) were bred for this study.

Wild animals	N/A
Reporting on sex	For the human xenografts, only female mice were used. For Th-MYCN colony studies both male and female mice were used.
Field-collected samples	N/A
Ethics oversight	MCF7 mouse xenograft studies were performed at Oncodesign under regulations from the Canadian Council on Animal Care and the National Research Council Guide.<br>CHP134 mouse xenograft studies were performed at Repare Therapeutics in a vivarium accredited by the Canadian Council on Animal Care with an Institutional Animal Care Committee-approved protocol.<br>Neuroblastoma xenograft experiments were conducted at the Children's Hospital of Philadelphia (CHOP) and approved by the Institutional Animal Care and Use Committee (IACUC #000643).<br>Experiments involving Th-MYCN mice were conducted at the Children's Cancer Institute and approved by the University of New South Wales Animal Care and Ethics Committee (ACEC #22/145B), in accordance with the Animal Research Act 1985 (New South Wales, Australia) and the Australian Code for the Care and Use of Animals for Scientific Purposes (2013).

Note that full information on the approval of the study protocol must also be provided in the manuscript.

Plants

Seed stocks	N/A
Novel plant genotypes	N/A
Authentication	N/A
