# Supplementary figures and images for: The PLK4 inhibitor RP-1664 demonstrates potent efficacy in neuroblastoma preclinical models through a dual mechanism of sensitivity
Source: Nat Commun. 2026 Jun 13;17:7531. doi: 10.1038/s41467-026-74061-5 (PMC13408883; doi:10.1038/s41467-026-74061-5)

Figure 1A.

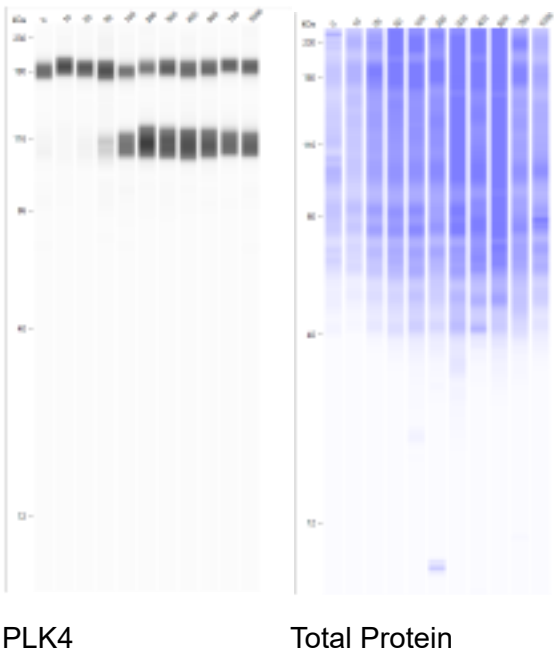

Figure 1E.

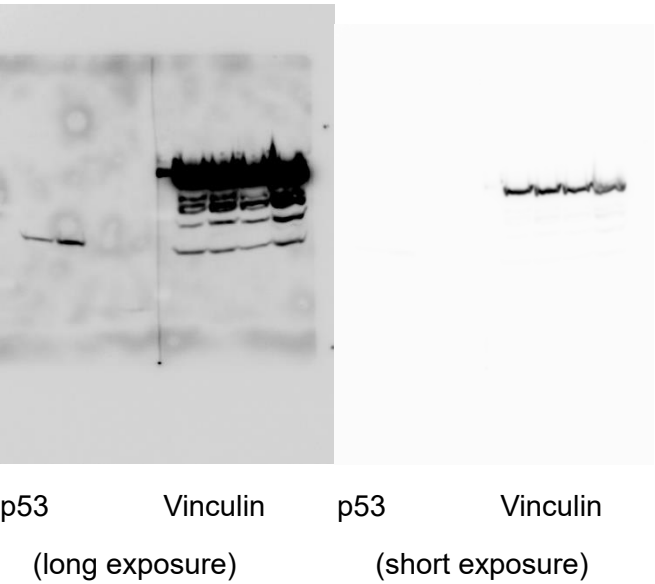

Figures 1E and 3D.

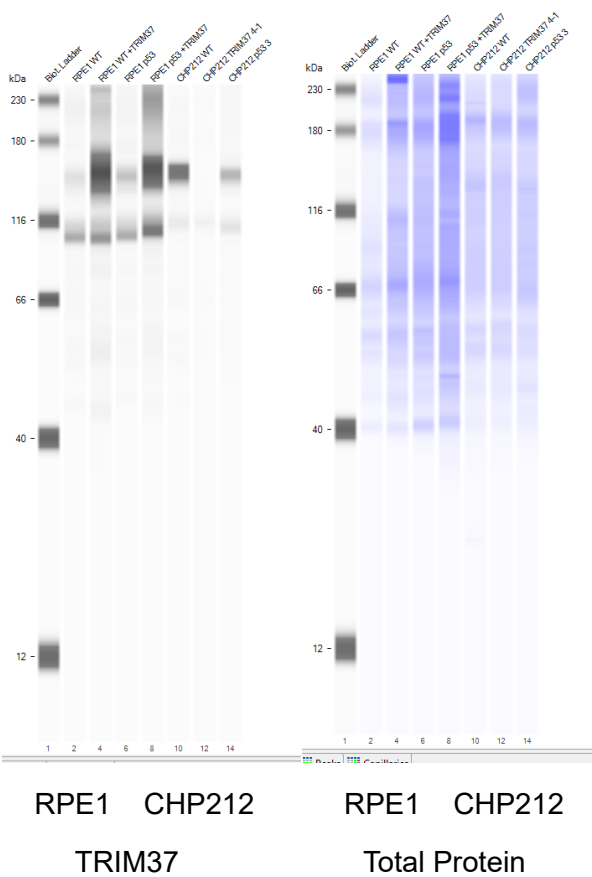

**Figure 3C.**

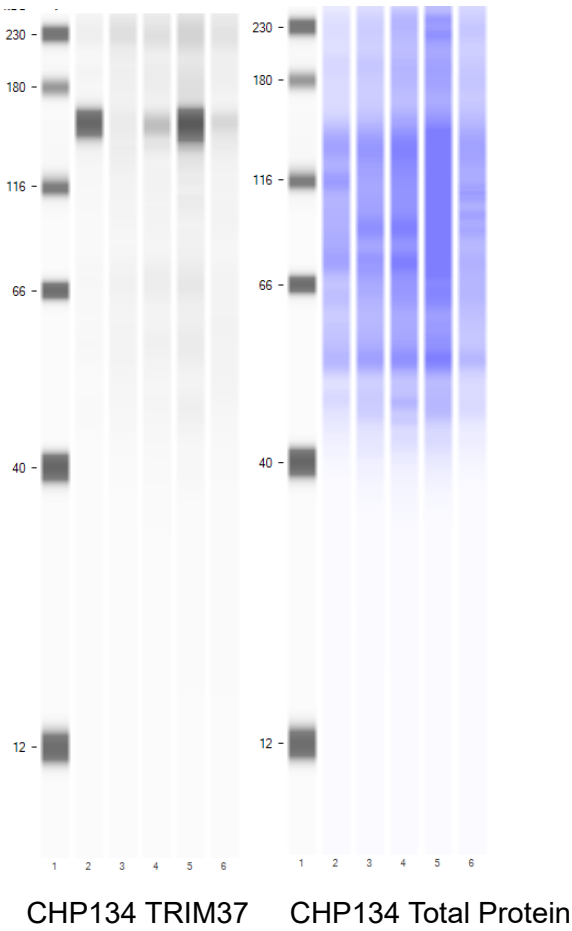

**Figure 5C.**

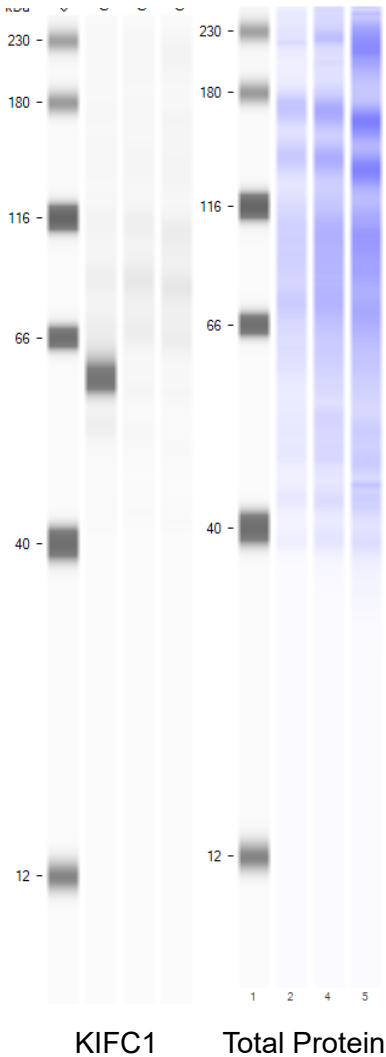

**Figures 3C and 3D.**

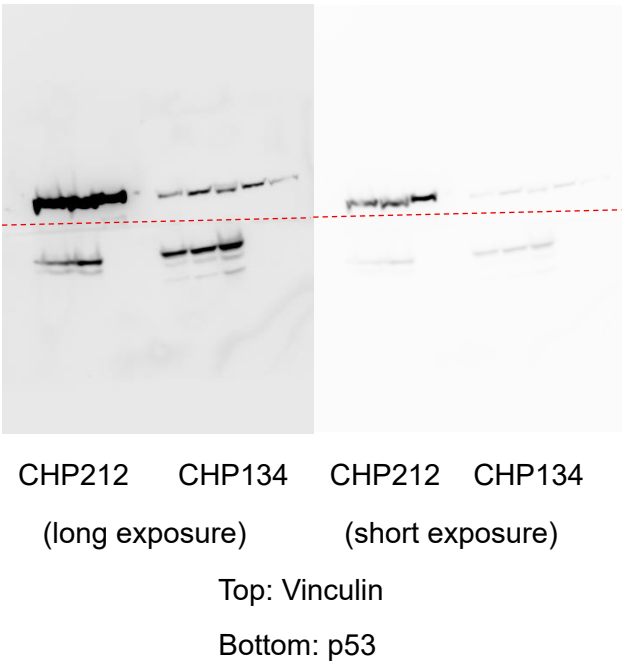

Supplement: Supplementary file 14 — Source Data [file 41467_2026_74061_MOESM14_ESM.zip › SourceData_uncropped_blots.pdf]
