# Supplementary material for: The PLK4 inhibitor RP-1664 demonstrates potent efficacy in neuroblastoma preclinical models through a dual mechanism of sensitivity
Source: Nat Commun. 2026 Jun 13;17:7531. doi: 10.1038/s41467-026-74061-5 (PMC13408883; doi:10.1038/s41467-026-74061-5)
Supplement: Supplementary file 13 — Transparent Peer Review file [file 41467_2026_74061_MOESM13_ESM.pdf]

nature portfolio

Peer Review File

PLK4 inhibitor RP-1664 demonstrates efficacy in neuroblastoma preclinical models through a dual mechanism of sensitivity

Corresponding Author: Dr John Maris

This file contains all reviewer reports in order by version, followed by all author rebuttals in order by version.

Version 0:

Reviewer comments:

Reviewer #1

(Remarks to the Author)
Review of Manuscript
In this study, the authors characterize the PLK4 inhibitor RP-1664 as a potential therapeutic agent. They examine how this inhibitor affects centriole depletion or amplification in a dose-dependent manner and show that it impacts cell proliferation in a TRIM37- and p53-dependent manner. The authors highlight that TRIM37 gain is a feature of neuroblastoma and further investigate the effect of RP-1664 on neuroblastoma growth. They find that both low and high concentrations of RP-1664 reduce neuroblastoma growth through two distinct mechanisms: high concentrations cause centrosome depletion, which is synthetic lethal with TRIM37 gain, whereas low concentrations induce centrosome amplification, leading to multipolar mitosis—a phenomenon attributed to neuroblastoma cells' inability to cluster amplified centrosomes. Finally, the authors test RP-1664 efficacy in patient-derived xenograft models and a neuroblastoma mouse model.

Overall, the authors convincingly show the effects of RP-1664 on centrosome dynamics and neuroblastoma sensitivity. The manuscript would be further strengthened by more clearly establishing and emphasizing the novel aspects of these findings.

Strengths
1. A key novel finding is that low doses of RP-1664 induce centrosome amplification and consequent multipolar mitosis. Neuroblastoma cells are particularly sensitive to centrosome amplification due to their inability to cluster extra centrosomes, revealing a TRIM37- and p53-independent vulnerability.
2. RP-1664 appears to have improved pharmacokinetic properties, making it suitable for in vivo studies, unlike Centrinone.

Weaknesses / Areas for Improvement
1. The novel finding that low doses of RP-1664 induce neuroblastoma-specific sensitivity could be explored in more depth. Specifically, the manuscript does not fully address why neuroblastoma cells are uniquely sensitive or whether this effect might extend to other cell types. Additional mechanistic insight would strengthen the claim of specificity.
2. Some parts of the manuscript recapitulate previously reported observations with other PLK4 inhibitors, including synthetic lethality with TRIM37 gain/amplification (PMID: 32908304; PMID: 32908313) and activation of the mitotic surveillance ("stopwatch") pathway leading to p53-mediated cell cycle arrest or death (PMID: 38547292; PMID: 27371829; PMID: 27432897; PMID: 27432896). While these findings are important for context, clarifying the novel contributions of RP-1664 relative to prior studies would help emphasize the manuscript's originality.

Major Comments

1. A key novelty of this manuscript is the observation that neuroblastoma cells fail to cluster centrosomes. Determining the underlying defect should be experimentally feasible and would substantially strengthen the novelty and mechanistic depth of the study. This point needs to be addressed. A possible approach is to test for defects in known clustering mechanisms (e.g., HSET/KIFC1, dynein–NuMA cortical anchoring, microtubule stability, spindle geometry). Rescue experiments with candidate factors could confirm causality.

2. The genetic screen in CHP134 TRIM37-low TP53-KO cells identifies multiple PIDDosome components, suggesting that cell death in this context may be mediated by PIDDosome activation rather than solely by multipolar mitosis. A recent study (PMID: 39475598) demonstrates that the PIDDosome can trigger apoptosis via BID cleavage, providing a plausible alternative mechanism. The authors should evaluate the contribution of PIDDosome signaling by assessing BID cleavage, caspase activation, and performing rescue experiments through genetic or pharmacological inhibition of PIDDosome components.

3. None of the hits from the CHP134 screen have been validated by independent experiments. It is standard practice to confirm relevant hits from such screens, and unvalidated hits should not be used as the basis for conclusions. Validating at least one of the PIDDosome hits (see previous point) would strengthen confidence in the screen results and could also uncover a relevant mechanism underlying the sensitivity to low concentrations of PLK4 inhibitor.

4. The authors highlight PCNT and CDK5RAP2 in the RPE1 screen hits, but don't mention them at all. A paper from the Desai lab (PMID: 33170211) describes that PCNT and CDK5RAP2 are essential for cell division in PLK4-inhibited cells. Thus, impaired PCNT or CDK5RAP2 function in neuroblastoma could explain the sensitivity to PLK4 inhibition as well. The authors should investigate this possibility.

5. The authors claim that neuroblastoma cells are uniquely sensitive to centrosome amplification; however, the only comparison provided is with RPE1 cells. This is insufficient to establish neuroblastoma specificity. Additional comparisons with other cancer cell types are necessary to determine whether this is truly a neuroblastoma-specific vulnerability or a more general phenomenon. A possible approach is to extend the analysis to other cancer cell lines, ideally including both clustering-competent and clustering-deficient examples, to determine whether this is a neuroblastoma-specific vulnerability or a broader cancer cell phenotype.

6. The claims regarding the concentration ranges that cause centrosome depletion are inconsistent. The data show that 100 nM RP-1664 induces centrosome amplification, while 250 nM causes depletion, yet the text states that anything slightly above 100 nM leads to depletion. The authors should either present data for intermediate concentrations (100–250 nM) or revise their statements to align with the available evidence.

7. The authors must provide direct evidence that the concentrations used in the xenograft and models indeed result in centrosome amplification or depletion in vivo. Without such confirmation, it is difficult to interpret the mechanistic relevance of the in vivo findings. Suggested approach: Perform immunofluorescence on tumor sections for centrosome markers (e.g., γ-tubulin, centrin) to directly assess centrosome number in treated vs. control tumors.

8. Mechanistic basis of RP-1664 effects in the neuroblastoma mouse model is unclear. The in vivo effects of RP-1664 on neuroblastoma growth are insufficiently characterized. It remains unclear whether the observed outcomes can be attributed to centrosome amplification, centrosome depletion, or mechanisms entirely independent of centrosome number. As TRIM37 is likely not amplified in this model, it becomes even more important to investigate the mechanism underlying the drug's efficacy. Directly assessing centrosome status and related pathways in tumor tissue would strengthen the mechanistic link between the in vitro and in vivo findings.

Other Comments

1. Is the change in centrosome amplification significant in Figure 5B?

2. Figure 5A supposedly showing "centrosome clustering" doesn't provide any evidence for it. The weak spot to which the arrow points towards could be anything.

3. Line 82: Centriole duplication and de novo procentriole formation are two different things. A de novo formation relates to cells, which don't have a centrosome as template.

4. Figure 1A-B: The concentrations of RP-1664 used in this experiment should be shown.

5. In most experiments, the duration of RP-1664 treatment is not indicated. This information is essential to distinguish between short- and long-term effects of PLK4 inhibition. For example, centrosome depletion requires several days, whereas PLK4 stabilization can occur within just a few hours. Providing the treatment durations would help clarify the interpretation of the results.

6. Figure 8: The labeling of the X-axis "Time to Medium Palpable Tumor (days)" is confusing. As written, it could be interpreted to mean that mice die a certain number of days before the tumor becomes palpable. Perhaps the authors intended to indicate "Time since palpable tumor (days)" or a similar measure. Please clarify.

Reviewer #2

(Remarks to the Author)
Previous studies established that PLK4 inhibition induces synthetic lethality in TRIM37-high cancers. This manuscript extends that paradigm by characterizing the novel PLK4 inhibitor RP-1664 in high-risk neuroblastoma, a highly lethal pediatric cancer with a five-year survival rate of only ~50%. Neuroblastoma is marked by recurrent gain of chromosome 17q, which encompasses the TRIM37 gene.
The authors present compelling evidence of robust preclinical efficacy for RP-1664 across neuroblastoma cell lines, patient-derived xenografts, and a genetically engineered mouse model. Importantly, they uncover dual mechanisms of sensitivity that expand beyond the canonical TRIM37–PLK4 synthetic lethal interaction. At sub-saturating concentrations, RP-1664 induces centriole amplification through stabilization of PLK4 protein. Unlike normal cells, neuroblastoma cells are unable to cluster supernumerary centrosomes, resulting in lethal multipolar mitoses. This vulnerability was shown to be independent of TRIM37 status. At higher concentrations, RP-1664 fully blocks PLK4 catalytic activity, leading to centrosome depletion, which is synthetic lethal in the context of TRIM37 overexpression. This dual mechanism broadens the therapeutic scope of PLK4 inhibition in neuroblastoma and possibly other cancers.
Overall, this study provides a rigorous preclinical evaluation of PLK4 inhibition as a therapeutic strategy in neuroblastoma. While the underlying biology aligns with previously reported concepts, the work is likely to interest both scientists and clinicians given the strength of the in vivo data and the therapeutic implications. Additional mechanistic clarifications and targeted experiments could further enhance the impact of the manuscript.
Major Comments
1. The paper would benefit from additional characterization of the neuroblastoma models, particularly with respect to their centrosome clustering capacity. While it is clear that these cells fail to cluster supernumerary centrosomes (in contrast to RPE1), the underlying basis for this deficiency remains undefined. Assessing KIFC1 expression (at the genomic or protein level) could provide useful insights, and rescue experiments through KIFC1 overexpression or other approaches to promote clustering would directly test whether impaired clustering drives the observed phenotype. Although the authors discuss several possible breakdowns, demonstrating rescue through one of these routes would substantially strengthen the conclusions overall.

The authors conclude that neuroblastoma cells are deficient in centrosome clustering; however, their comparison is limited to RPE1 cells, a single non-transformed cell line. This narrow reference point may not fully capture the broader variability of centrosome clustering capacity across normal cell types. If centrosome clustering is generally restricted, the therapeutic window for low-dose PLK4 inhibition could be narrower than anticipated. To strengthen this conclusion, it would be valuable to assess centrosome clustering in additional non-transformed cell lines exposed to low concentrations of PLK4 inhibitor.

2. The authors suggest that cytotoxicity in TP53-mutant neuroblastoma may proceed through a PIDDosome–caspase-2 pathway independent of p53, but this remains largely inferential. It would strengthen the argument to test whether caspase-2 activation persists when PIDDosome components (e.g., PIDD1, RAIDD) are deleted in the neuroblastoma model (CHP134) used for the screen, and whether caspase-2 and downstream apoptotic effectors are engaged, or whether MDM2 cleavage is not observed in these contexts.

3. The xenograft models demonstrate clear efficacy, but direct staining of tumors to assess centrosome counts, rather than inferring mechanism by matching RP-1664 plasma levels in vivo to dose–response data from cell lines, would provide direct validation of whether efficacy reflects centriole depletion versus amplification.

4. The dual-mechanism model raises an important translational question: should clinical dosing aim for centrosome amplification (low dose) or depletion (high dose)? The discussion section would be strengthened by elaborating on how this informs dose selection or treatment regimens, and whether distinct strategies are warranted across cancer types—for example, amplification as a potential strategy in neuroblastoma versus depletion as the strategy in breast cancers harboring 17q23 amplification, where low doses may still permit pseudobipolar divisions.
Minor Comments
1. The manuscript does not provide specific information on the selectivity profile of the PLK4 inhibitor with respect to known off-targets, such as Aurora kinases. While these data may be available in prior publications on this molecule, it would be useful to include a brief summary or citation here for reference and context.
2. It would be helpful if the manuscript provided additional context regarding the tolerability concerns that necessitated reduced dosing of RP-1664. Specifically, is there evidence that RP-1664 inhibits mouse PLK4, and do the authors have any histopathological data that might clarify the basis of the observed body weight loss in treated mice? Including such information would strengthen the translational relevance of the preclinical findings.
3. In Extended Data Fig. 4b, “p21” appears to be misannotated and should be “p53.”
4. In Extended Data Fig. 7c, it would ease interpretation if mice requiring treatment interruption due to weight loss were clearly indicated in the figure.
5. The boxed label of PLK4 in Fig. 1c is not clearly visible, and it is unclear whether this is intentional, which could lead to misinterpretation.

Reviewer #3

(Remarks to the Author)
This is a highly interesting and very detailed study of the therapeutic potential of PLK4 inhibition in the context of high-risk neuroblastoma marked by 17q/TRIM37 gain.

I would like to express my deep appreciation of the in-depth functional analyses of the intriguing bimodal impact of low versus high PLK4 inhibitor concentrations as well as the extensive in vivo analysis involving CDX, PDX and immunocompetent murine models.

However, I would like to address that in comparison to established literature that the obtained findings are not truly original including:

(1) 17q as pathognomic biomarker of high-risk neuroblastoma (including ref: https://pubmed.ncbi.nlm.nih.gov/10379019/)

(2) Moreover, the mechanistic dissection of the PLK4-TRIM37 has been extensively demonstrated by Meitinger et al. (Nature 2020; TRIM37 controls cancer-specific
vulnerability to PLK4 inhibition, PMID: 32908304), including exploration of the therapeutic potential of PLK4 inhibition neuroblastoma CDX models. Moreover, similar mechanistic findings were elaborated on in breast cancer (Yeow et al., Nature, 2020, PMID: 32908313).

(3) Last, the key mechanistic link with KIF1C was demonstrated for the first time by Zhou et al. in endometrial cancer (KIFC1 depends on TRIM37-mediated ubiquitination of PLK4 to promote centrosome amplification in endometrial cancer; PMID: 39349439).

It would be very interesting if the authors could further elaborate functionally/mechanistically on the link with the USP28-53BP1 axis as well as the PIDDosome?

Given the very important and elaborate preclinical work on PLK4 inhibition in this manuscript with various neuroblastoma murine models, I would recommend to transfer this publication eg to Cell Reports Medicine.

Furthermore, although the data provided in this manuscript show a strong case for single PLK4 inhibition, I think it is always valuable to also scrutinize for potential synergistic drug interactions and would be of great added value to this manuscript.

Reviewer #4

(Remarks to the Author)
I co-reviewed this manuscript with one of the reviewers who provided the listed reports. This is part of the Nature Communications initiative to facilitate training in peer review and to provide appropriate recognition for Early Career Researchers who co-review manuscripts.

Version 1:

Reviewer comments:

Reviewer #1

(Remarks to the Author)
Unfortunately, I do not believe that this manuscript provides substantial novel mechanistic insight. Rather, it largely confirms previously established findings showing that either TRIM37 amplification or activation of the mitotic surveillance pathway (USP28–TP53BP1–p53) can sensitize cancer cells to PLK4 inhibition.

The authors report that CHP134 cells are sensitive to RP-1664–induced centrosome amplification and present this as a novel observation. However, their genetic screen identifies BID, which has already been shown to mediate apoptosis in response to centrosome amplification through a PIDDosome-dependent but p53-independent mechanism (PMID: 39475598). Thus, the primary conclusion of the manuscript appears to be that BID-mediated apoptosis contributes to cell death specifically in CHP134 cells. While this mechanism can explain most of the reported observations, the manuscript does not sufficiently acknowledge or discuss this prior work, and the relevance of BID-mediated apoptosis is not given appropriate attention in the discussion.

In addition, the manuscript contains several incorrect or misleading statements. A few examples are listed below.

1. Misrepresentation of prior work
The abstract states that PLK4 inhibition was recently shown to be synthetic lethal with TRIM37 amplification in a p53-dependent manner. This statement is incorrect. The synthetic lethality between TRIM37 amplification and PLK4 inhibition has been clearly demonstrated to be p53-independent. The abstract therefore presents the prior literature inaccurately and creates the misleading impression that the authors are reporting a new p53-independent mechanism.

2. Overstated translational claims
Because the manuscript does not establish a novel mechanism, several claims in the abstract appear overstated or insufficiently supported by the data. For example, the authors conclude that “these data support biomarker-directed clinical

development of PLK4 inhibitors for high-risk neuroblastoma and other cancers with somatically acquired TRIM37 overexpression." However, the manuscript simultaneously attempts to argue that sensitivity to PLK4 inhibition is independent of TRIM37 status. Moreover, the proposed link to centrosome clustering defects is not supported by the identification of a clear biomarker. MYCN is mentioned, but no direct mechanistic connection between MYCN expression and PLK4 inhibitor sensitivity is demonstrated. Making biomarker claims without clear supporting evidence is therefore premature.

3. Insufficient evidence for TRIM37- and p53-independent sensitivity
To convincingly claim that PLK4 inhibitor sensitivity is independent of both TRIM37 and p53 in a clinically relevant context, additional experimental evidence would be required. For example, the authors could demonstrate sensitivity in double knockout models lacking both TRIM37 and p53 (e.g., in the context of Figure 3G), or alternatively test additional CDX or PDX models with normal TRIM37 levels and p53 inactivation.

Despite these concerns, I believe that the study contains valuable data and could make a useful contribution if the manuscript were reframed. Rather than presenting the findings as a fundamentally new mechanism, the authors could position the work as a systematic evaluation of multiple pathways that contribute to sensitivity to PLK4 inhibition.

A possible restructuring could include the following points:

1. Use the data currently shown in Figures 1 and 4 to confirm previously described mechanisms through which PLK4 inhibition can induce cell death, including
i)TRIM37 amplification
ii) Mitotic surveillance (USP28–TP53BP1–p53 pathway)
iii) PIDDosome activation in either a p53-dependent manner (via MDM2 cleavage) or a p53-independent manner (via BID cleavage).

2. Present the data in Figure 3 as preliminary evidence that multipolar mitosis and defects in centrosome clustering may additionally contribute to sensitivity.

3. Highlight the analysis of TRIM37 amplification in high-risk neuroblastoma (currently Figure 2) to emphasize the value of TRIM37 as a biomarker.

4. Present the xenograft experiments in TRIM37-amplified neuroblastoma models (currently Figure 6).

5. Finally, include the data suggesting that PLK4 inhibition may also have efficacy beyond TRIM37-amplified tumors (currently Figure 7).

Reframing the manuscript in this manner would provide a clearer and more balanced presentation of the data while aligning the conclusions more closely with the existing literature.

Reviewer #2

(Remarks to the Author)
The authors have made substantial efforts to address the reviewers' concerns, noting that some limitations were unavoidable due to the recent lab closure at Repare Therapeutics. I have one remaining concern that should be resolved prior to publication.

In response to my question regarding tolerability, the authors state: "the dosing of PLK4 inhibitors is known to be limited by myelosuppression, which is also likely the reason necessitating intermittent dosing in our current experiments (PMID: 40378279, 38114624)." However, one of the cited references characterizes CFI-400945, a molecule widely recognized to have significant Aurora B inhibitory activity; thus, any observed immunosuppression phenotype in that context is likely attributable to Aurora B inhibition rather than selective PLK4 targeting. The second cited study, from the Repare team, does not report myelosuppression, although it does note that continuous dosing with RP-1664 at 600 ppm was not tolerated.

Given this, I strongly recommend that the authors include data demonstrating the effects of RP-1664 on peripheral blood counts in mice. Such data would be highly informative and would provide important support for the anticipated on-mechanism hematologic toxicities associated with PLK4 inhibition in the clinical setting.

Reviewer #3

(Remarks to the Author)
no further comments, acceptance for publication

Open Access This Peer Review File is licensed under a Creative Commons Attribution 4.0 International License, which permits use, sharing, adaptation, distribution and reproduction in any medium or format, as long as you give appropriate credit to the original author(s) and the source, provide a link to the Creative Commons license, and indicate if changes were made.
In cases where reviewers are anonymous, credit should be given to 'Anonymous Referee' and the source.
The images or other third party material in this Peer Review File are included in the article's Creative Commons license, unless indicated otherwise in a credit line to the material. If material is not included in the article's Creative Commons license and your intended use is not permitted by statutory regulation or exceeds the permitted use, you will need to obtain permission directly from the copyright holder.
To view a copy of this license, visit https://creativecommons.org/licenses/by/4.0/

We thank the Reviewers for their thorough and insightful comments and provide our point-by-point responses below, pointing to changes in the clean version of the revised manuscript whenever possible. While we made an earnest effort to address all comments, we would like to note that, as our revisions were in progress, the laboratory responsible for most functional cell biology studies (under supervision of M. Zimmermann at Repare Therapeutics) was shut down due to company restructuring. As such, our capability to address points surrounding mechanistic centrosome biology and cell death pathways was severely limited. Together with the fact that the some of the experiments as suggested by Reviewers are rather extensive in scope and may constitute a wholly separate line of investigation (as outlined below in more detail), we strongly favor leaving these for future studies. On the other hand, we have substantially strengthened the manuscript by including new data on i) drug sensitivity window between neuroblastoma and non-transformed cell lines; ii) evidence of RP-1664-induced multipolar mitosis *in vivo*; and iii) unprecedented combinatorial anti-tumor activity of RP-1664 and standard-of-care chemoimmunotherapy showing a clear path for clinical translation. We think that these data provide significant new evidence supporting the paper's conclusions and clinical development of PLK4 inhibition in high-risk neuroblastoma. Most importantly, this manuscript will provide all the preclinical justification required for a clinical trial being developed for children with relapsed high-risk neuroblastoma.

Reviewer #1

Major Comments

1. A key novelty of this manuscript is the observation that neuroblastoma cells fail to cluster centrosomes. Determining the underlying defect should be experimentally feasible and would substantially strengthen the novelty and mechanistic depth of the study. **This point needs to be addressed.** A possible approach is to test for defects in known clustering mechanisms (e.g., HSET/KIFC1, dynein–NuMA cortical anchoring, microtubule stability, spindle geometry). Rescue experiments with candidate factors could confirm causality.

To address this point, we mined public cell line databases (DepMap and The Protein Atlas [TPA]) for RNA expression of genes known to influence centrosome clustering. Among these candidates, MYO10, a gene encoding an atypical microtubule-binding myosin, displays the most pronounced downregulation in neuroblastoma cell lines both in DepMap as well as TPA (**Rebuttal Figure 1A,B**).

While we think that this approach may provide initial insights into the mechanism underlying centrosome clustering defects in neuroblastoma cells, a full elucidation of this mechanism as suggested by the Reviewer is important but arguably beyond the scope of the current study. In fact, it may constitute a separate manuscript altogether. In our current paper, we sought to demonstrate the point that PLK4 inhibition is a promising candidate therapeutic strategy for high-risk neuroblastoma and to provide extensive pre-clinical validation of this hypothesis in a range of highly disease-relevant models, well beyond what is provided in existing literature. We also show mechanistically that this is not only due to *TRIM37* overexpression, as suggested by prior studies, but also due to the contribution of a *TRIM37*-independent pathway. At the same time, unraveling the molecular details of this pathway will require substantial investigation, which we would prefer to leave for future studies, as we now emphasize in the discussion **on pages 18-19, lines 459-464**, and copied in below:

"In addition to KIFC1, over 30 genes are already known to facilitate centrosome clustering[48], including factors involved in microtubule organization, the spindle assembly checkpoint, sister chromatid cohesion, as well as the actin cytoskeleton and cell adhesion[47,49,57–61]. Future studies

will be needed to assess the mutational status or altered expression of these genes in neuroblastoma and whether these contribute to centrosome clustering defects."

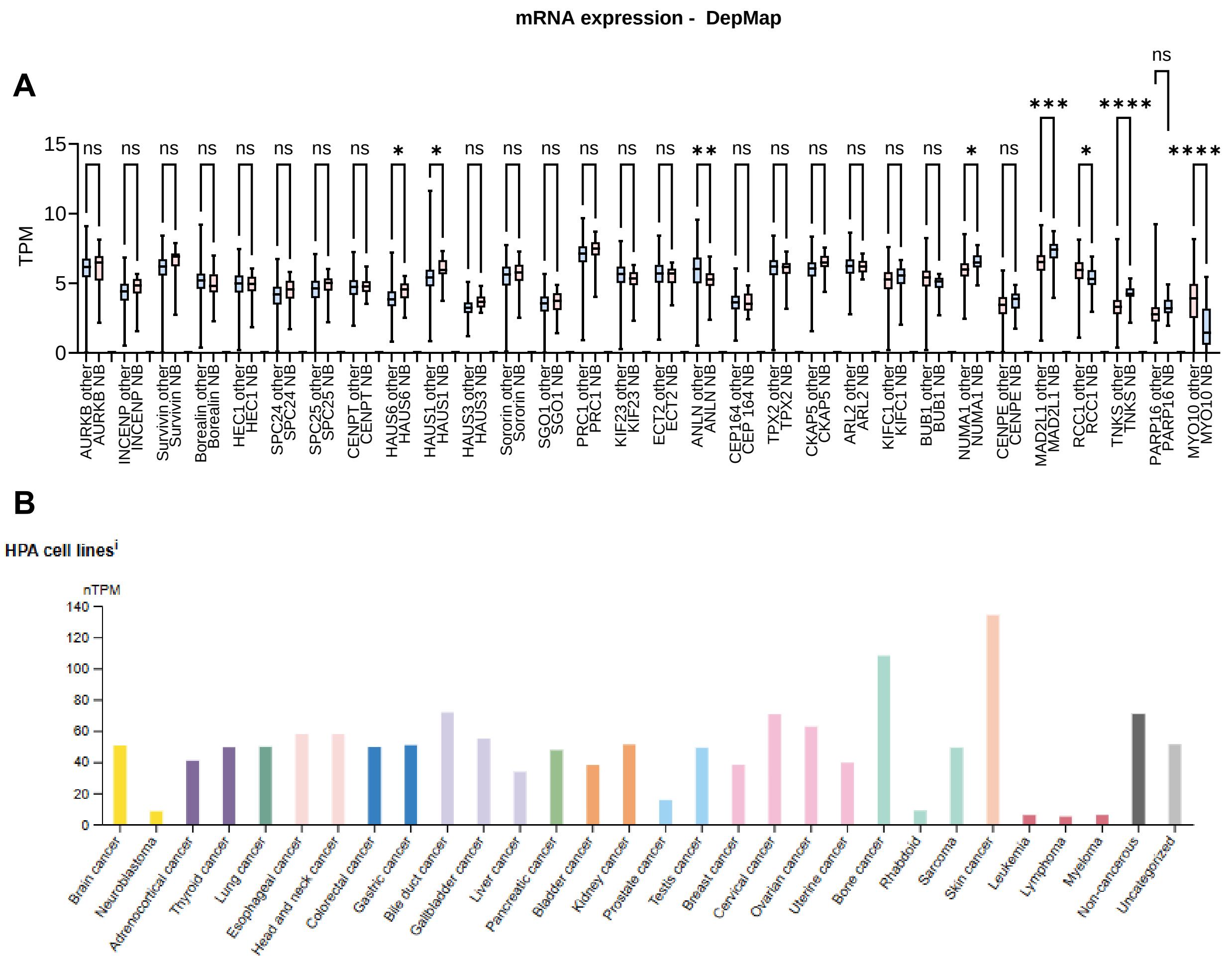

Rebuttal Figure 1: A. mRNA expression of selected genes related to centrosome clustering in DepMap in neuroblastoma cell lines vs. other cell lineages. B. MYO10 mRNA expression across cell line lineages in The Protein Atlas.

2. The genetic screen in CHP134 TRIM37-low TP53-KO cells identifies multiple PIDDosome components, suggesting that cell death in this context may be mediated by PIDDosome activation rather than solely by multipolar mitosis. A recent study (PMID: 39475598) demonstrates that the PIDDosome can trigger apoptosis via BID cleavage, providing a plausible alternative mechanism. The authors should evaluate the contribution of PIDDosome signaling by assessing BID cleavage, caspase activation, and performing rescue experiments through genetic or pharmacological inhibition of PIDDosome components.

We agree that this is an important point and that PIDDosome-mediated signaling represents a plausible additional mechanism of RP-1664–induced cell death. We attempted to downregulate PIDD1 in neuroblastoma cells to evaluate the effects on RP-1664 sensitivity, p53 activation, and cell death. Unfortunately, we were only able to achieve transient and incomplete PIDD1 depletion that did not allow us to reliably assess its contribution to BID cleavage, caspase activation, or RP-1664–induced cell death in these models. As we include extensive new data supporting the translational relevance of our studies and due to operational reasons outlined above, we therefore

favor leaving a detailed mechanistic dissection of PIDDosome-dependent versus PIDDosome-independent cell death pathways to future studies.

3. None of the hits from the CHP134 screen have been validated by independent experiments. It is standard practice to confirm relevant hits from such screens, and unvalidated hits should not be used as the basis for conclusions. Validating at least one of the PIDDosome hits (see previous point) would strengthen confidence in the screen results and could also uncover a relevant mechanism underlying the sensitivity to low concentrations of PLK4 inhibitor.

Please see our response to the previous point.

4. The authors highlight PCNT and CDK5RAP2 in the RPE1 screen hits, but don't mention them at all. A paper from the Desai lab (PMID: 33170211) describes that PCNT and CDK5RAP2 are essential for cell division in PLK4-inhibited cells. Thus, impaired PCNT or CDK5RAP2 function in neuroblastoma could explain the sensitivity to PLK4 inhibition as well. The authors should investigate this possibility.

Analogous to our response to point 1, we have compared DepMap mRNA expression data of PCNT and CDK5RAP2 in neuroblastoma versus all other cancer cell lines (**Rebuttal Figure 2**). This preliminary analysis did not reveal impaired CDK5RAP2 and PCNT expression in neuroblastoma – in fact their expression may be slightly increased. While this result will need to be confirmed functionally, it argues against the hypothesis of impaired CDK5RAP2 or PCNT being the drivers of neuroblastoma sensitivity to RP-1664. For the reasons argued above, we prefer to leave this exploration for future studies.

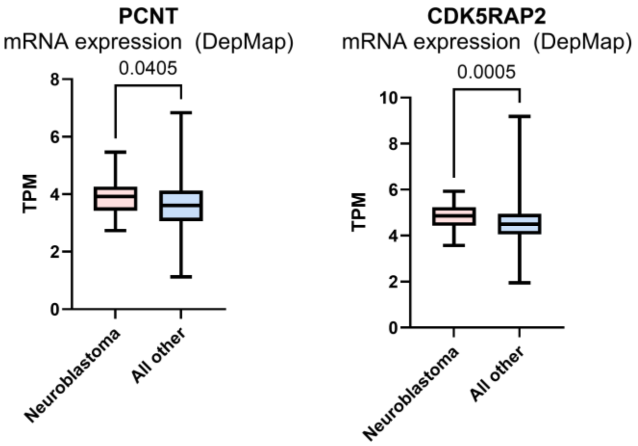

Rebuttal Figure 2: A. mRNA expression of PCNT and CDK5RAP2 in DepMap in neuroblastoma cell lines vs. other cell lineages.

5. The authors claim that neuroblastoma cells are uniquely sensitive to centrosome amplification; however, the only comparison provided is with RPE1 cells. This is insufficient to establish neuroblastoma specificity. Additional comparisons with other cancer cell types are necessary to determine whether this is truly a neuroblastoma-specific vulnerability or a more general phenomenon. A possible approach is to extend the analysis to other cancer cell lines, ideally including both clustering-competent and clustering-deficient examples, to determine whether this is a neuroblastoma-specific vulnerability or a broader cancer cell phenotype.

We agree with the Reviewer that establishing whether this represents a neuroblastoma-specific vulnerability is a key point. We have therefore now compared the sensitivity of TRIM37 gain-carrying neuroblastoma cell lines to a panel of nine breast cancer cell lines, which also carry at least one extra copy of *TRIM37*. As shown in revised **Extended Data Figure 2A and Rebuttal Figure 3**, neuroblastoma cell lines are overall more sensitive to RP-1664 than breast cancer cell lines. In fact, only 4 breast cancer cell lines show IC_{50} values at or below 100nM RP-1664, the concentration range required for centrosome amplification, compared to 8 of 9 of neuroblastoma cell lines. Neuroblastoma cell sensitivity is also increased when compared to four non-transformed cell lines (**Extended Data Figure 2A and Rebuttal Figure 3)**, supporting both a neuroblastoma-biased vulnerability and the potential for a favorable therapeutic index.

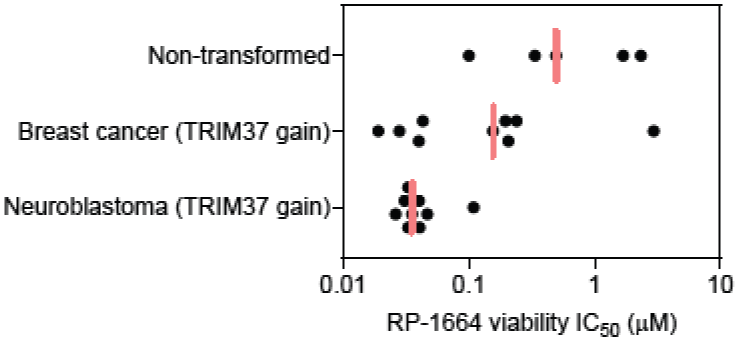

Rebuttal Figure 3: RP-1664 sensitivity (CellTiter Glo or Incucyte growth assays) in non-transformed cell lines, breast cancer cell lines with TRIM37 copy number gain and neuroblastoma cell lines with TRIM37 copy number gain.

Finally, we think that sensitivity to centriole amplification may not be strictly limited to neuroblastoma. In our hands, most neuroblastoma cell lines are sensitive to centriole amplification, which makes this disease an attractive candidate patient population, but does not exclude the likelihood of finding additional tumor types showing similar sensitivity. We are currently investigating the prevalence of this phenomenon across tumor types, as its presence has been indicated e.g. by a recent Moreno-Marin and colleagues 2023 bioRxiv pre-print: https://doi.org/10.1101/2023.03.13.532472 reporting widespread dependence on centrosome clustering in several non-neuroblastoma malignancies. As these investigations are quite extensive in nature and constitute a separate study, our strong preference would be to keep the current manuscript focused on neuroblastoma, and we now address this important point in the discussion on **page 19, lines 473-476**, and copied in below:

"Of note, in a recent pre-print, Moreno-Marin et al. describe a remarkable diversity in centrosome clustering proficiency among the NCI60 cancer cell line panel, suggesting that sensitivity to centrosome overduplication may be found in additional tumor lineages[69]."

6. The claims regarding the concentration ranges that cause centrosome depletion are inconsistent. The data show that 100 nM RP-1664 induces centrosome amplification, while 250 nM causes depletion, yet the text states that anything slightly above 100 nM leads to depletion. The authors should either present data for intermediate concentrations (100–250 nM) or revise their statements to align with the available evidence.

We thank the Reviewer for pointing out the apparent discrepancies. While we think that concentrations as low as 150nM lead to centriole and centrosome depletion, as evidenced by inactivation of TRIM37, TP53BP1 and USP28 causing resistance to 150nM RP-1664 in the RPE1 CRISPR/Cas9 screen in **Figure 4C**, we acknowledge that we did not test concentrations between

100 and 250nM in immunofluorescence assays and therefore cannot experimentally document the precise transition point between amplification and depletion in this range. We have now (i) updated the concentration range visualizations in our growth assay figures and (ii) revised the wording in the main text on **page 6, lines 144-146** to state that centrosome depletion is observed at 250 nM, while functional assays suggest that the onset of depletion likely occurs between 150 and 250 nM RP-1664, thereby aligning our statements with the available evidence.

7. The authors must provide direct evidence that the concentrations used in the xenograft and models indeed result in centrosome amplification or depletion in vivo. Without such confirmation, it is difficult to interpret the mechanistic relevance of the in vivo findings. Suggested approach: Perform immunofluorescence on tumor sections for centrosome markers (e.g., γ-tubulin, centrin) to directly assess centrosome number in treated vs. control tumors.

We attempted to analyze centriole and centrosome numbers in FFPE sections, which proved very challenging due to the three-dimensional nature of the tissue – only a subset of centrosomes was quantifiable in any given tissue section. This is consistent with observations in the literature (e.g., https://pubmed.ncbi.nlm.nih.gov/36069081/), which highlight similar technical limitations when attempting to quantify centrosomes in FFPE material.

To overcome this limitation, we quantified multipolar mitoses in H&E-stained FFPE sections, as a proximal readout of centrosome amplification. As **Extended Data Figure 6I,J** and **Rebuttal Figure 4** show, RP-1664 treatment at an efficacious dose (450ppm) induces multipolar cell division in a PDX tumor model, suggesting that centrosome amplification may indeed contribute to the compound's anti-tumor activity *in vivo* and that the concentrations used in the xenograft experiments are sufficient to perturb centrosome number.

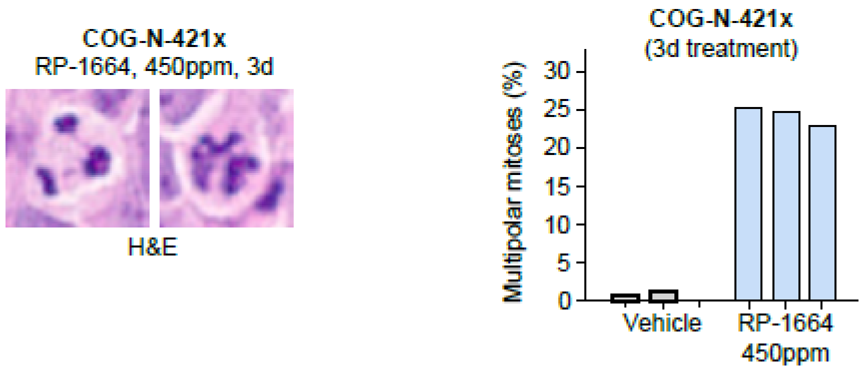

Rebuttal Figure 4: Left – example images of multipolar mitoses in RP-1664-treated COG-N-421x tumors (H&E stain). Right – frequency of multipolar cell division in vehicle- or RP-1664-treated tumors.

8. Mechanistic basis of RP-1664 effects in the neuroblastoma mouse model is unclear. The in vivo effects of RP-1664 on neuroblastoma growth are insufficiently characterized. It remains unclear whether the observed outcomes can be attributed to centrosome amplification, centrosome depletion, or mechanisms entirely independent of centrosome number. As TRIM37 is likely not amplified in this model, it becomes even more important to investigate the mechanism underlying the drug's efficacy. Directly assessing centrosome status and related pathways in tumor tissue would strengthen the mechanistic link between the in vitro and in vivo findings.

To demonstrate target engagement in our Th-MYCN mouse neuroblastoma model, we now show in revised **Extended Data Figure 8B,C** and **Rebuttal Figure 5** that efficacious doses of RP-1664 lead to an expected increase in PLK4 protein.

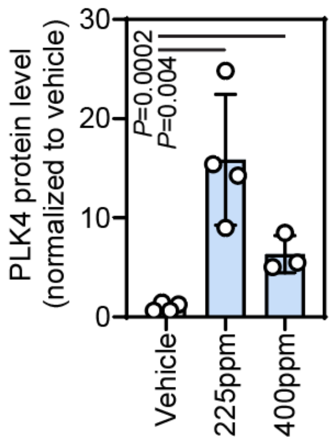

Rebuttal Figure 5: PLK4 protein level in vehicle- and RP-1664-treated Th-MYCN tumors.

Other Comments

1. Is the change in centrosome amplification significant in Figure 5B?

The frequencies of multipolar mitoses in neuroblastoma cell lines and RPE1 cells are indeed significant. We have added *P* values to a revised **Figure 5B**.

2. Figure 5A supposedly showing “centrosome clustering” doesn’t provide any evidence for it. The weak spot to which the arrow points towards could be anything.

We have revised the figure with increased temporal resolution, making the centrosome clustering more apparent. We also point the Reviewer to **Supplementary Video 4**.

3. Line 82: Centriole duplication and de novo procentriole formation are two different things. A de novo formation relates to cells, which don’t have a centrosome as template.

We thank the Reviewer for pointing out this mistake. We have revised the respective sentence to read “duplication of the parent centriole”.

4. Figure 1A-B: The concentrations of RP-1664 used in this experiment should be shown.

We have updated **Figure 1A** with RP-1664 concentrations.

5. In most experiments, the duration of RP-1664 treatment is not indicated. This information is essential to distinguish between short- and long-term effects of PLK4 inhibition. For example, centrosome depletion requires several days, whereas PLK4 stabilization can occur within just a few hours. Providing the treatment durations would help clarify the interpretation of the results.

This is an important point, and we have now added information regarding treatment duration in all applicable Figures. Generally, we use 48-72h (amounting to ~2 cell doublings) for functional assays (centrosome numbers, p21 activation, apoptosis…) and 4-12 days (~4-6 doublings) for growth assays *in vitro*. *In vitro* PLK4 stabilization and live cell experiments were conducted after 24h treatments.

6. Figure 8: The labeling of the X-axis “Time to Medium Palpable Tumor (days)” is confusing. As written, it could be interpreted to mean that mice die a certain number of days before the tumor becomes palpable. Perhaps the authors intended to indicate “Time since palpable tumor (days)” or a similar measure. Please clarify.

To avoid ambiguity and to ensure consistency with other animal experiments which simply label the x-axis with time units, we have altered the X-axis label to “Days”. The parameters for treatment start and endpoint are clearly explained in the methods.

Reviewer #2 (Remarks to the Author):

Major Comments

1. The paper would benefit from additional characterization of the neuroblastoma models, particularly with respect to their centrosome clustering capacity. While it is clear that these cells fail to cluster supernumerary centrosomes (in contrast to RPE1), the underlying basis for this deficiency remains undefined. Assessing KIFC1 expression (at the genomic or protein level) could provide useful insights, and rescue experiments through KIFC1 overexpression or other approaches to promote clustering would directly test whether impaired clustering drives the observed phenotype. Although the authors discuss several possible breakdowns, demonstrating rescue through one of these routes would substantially strengthen the conclusions overall.

As indicated in our response to a similar comment from Reviewer 1, we have assessed RNA expression of centrosome clustering-related genes in neuroblastoma cell lines in the DepMap and TPA databases. The gene *MYO10* showed significant downregulation in neuroblastoma cell lines as compared to other cell lineages (**Rebuttal Figure 1 A,B**), supporting the notion that altered expression of clustering regulators may contribute to the impaired centrosome clustering capacity of these models. However, we think that a full mechanistic validation including direct manipulation of KIFC1 or other clustering factors to rescue centrosome clustering and RP-1664 responses, lies beyond of the scope of the current study and would be better addressed in a dedicated follow-up manuscript (please see our response to Reviewer 1, point 1).

The authors conclude that neuroblastoma cells are deficient in centrosome clustering; however, their comparison is limited to RPE1 cells, a single non-transformed cell line. This narrow reference point may not fully capture the broader variability of centrosome clustering capacity across normal cell types. If centrosome clustering is generally restricted, the therapeutic window for low-dose PLK4 inhibition could be narrower than anticipated. To strengthen this conclusion, it would be valuable to assess centrosome clustering in additional non-transformed cell lines exposed to low concentrations of PLK4 inhibitor.

We agree with the Reviewer that additional non-transformed cell lines are informative, and we now show in revised **Extended Data Figure 2A/Rebuttal Figure 3** that non-cancerous cell lines (RPE1, MCF10A – originating from a fibrocystic mammary epithelium, COL-hTERT – an hTERT-immortalized colon cell line, BRONCH2 – SV40-immortalized bronchial epithelial cells, and HK2 – E6/E7-immortalized kidney epithelium) show on average substantially lower sensitivity to RP-1664 at concentrations that induce centrosome amplification as compared to neuroblastoma.

2. The authors suggest that cytotoxicity in TP53-mutant neuroblastoma may proceed through a PIDDosome–caspase-2 pathway independent of p53, but this remains largely inferential. It would strengthen the argument to test whether caspase-2 activation persists when PIDDosome components (e.g., PIDD1, RAIDD) are deleted in the neuroblastoma model (CHP134) used for the screen, and whether caspase-2 and downstream apoptotic effectors are engaged, or whether MDM2 cleavage is not observed in these contexts.

As outlined in our response to Reviewer #1, point 2, we have attempted to deplete PIDD1 from neuroblastoma cells to address this question, but were limited by incomplete and transient downregulation. We would prefer to leave the investigation of cell death mechanisms to future studies.

3. The xenograft models demonstrate clear efficacy, but direct staining of tumors to assess centrosome counts, rather than inferring mechanism by matching RP-1664 plasma levels in vivo to dose–response data from cell lines, would provide direct validation of whether efficacy reflects centriole depletion versus amplification.

In our experience, directly analyzing centrosome numbers in FFPE samples suffers from significant technical challenges (please see response to Reviewer 1, comment 7); therefore, we instead quantified multipolar mitoses in RP-1664-treated tumor sections as an indirect but functional readout of centrosome amplification. As shown in **Rebuttal Figure 4** and **Extended Data Figure 6I,J,** RP-1664-treated tumors display an elevated frequency of multipolar cell division compared with vehicle-treated controls, suggesting that they undergo centrosome overduplication at therapeutically effective doses of RP-1664 in vivo.

4. The dual-mechanism model raises an important translational question: should clinical dosing aim for centrosome amplification (low dose) or depletion (high dose)? The discussion section would be strengthened by elaborating on how this informs dose selection or treatment regimens, and whether distinct strategies are warranted across cancer types—for example, amplification as a potential strategy in neuroblastoma versus depletion as the strategy in breast cancers harboring 17q23 amplification, where low doses may still permit pseudobipolar divisions.

We thank the Reviewer for this suggestion and have amended the Discussion to reflect this important point as follows (**page 20, lines 492-500**):

"The contribution of centriole overduplication to PLK4 inhibitor efficacy raises important translational questions. It is not unreasonable to imagine that in mice and humans both mechanisms of sensitivity may operate in parallel, depending on the dose, schedule, pharmacokinetic profile of a given inhibitor and local concentration of RP-1664 in each tumor area. While monitoring centrosome numbers in tumor tissues is non-trivial[72], emergence of multipolar mitoses may provide a useful pharmacodynamic readout during PLK4 inhibitor clinical dose optimization and should be taken into consideration when interpreting clinical data in distinct patient populations (e.g. neuroblastoma vs. other TRIM37-overexpressing tumor types, in which higher-dose depletion strategies may predominate)."

Minor Comments

1. The manuscript does not provide specific information on the selectivity profile of the PLK4 inhibitor with respect to known off-targets, such as Aurora kinases. While these data may be available in prior publications on this molecule, it would be useful to include a brief summary or citation here for reference and context.

We thank the Reviewer for the important comment. While the selectivity profile of RP-1664 has been discussed in detail in Vallée et al., 2025, J. Med. Chem. (reference 27 in the current manuscript), we have amended the Introduction to summarize the selectivity and oral bioavailability of RP-1664 (**page 4, lines 104-107**) as follows:

"RP-1664 is highly selective for PLK4: it does not engage the majority of ~300 kinases in mammalian cells and shows over 2000-fold selectivity against the structurally related Aurora A and B kinases[27]. RP-1664 also displays favorable ADME properties and oral bioavailability in multiple pre-clinical mammalian species[27],…"

2. It would be helpful if the manuscript provided additional context regarding the tolerability concerns that necessitated reduced dosing of RP-1664. Specifically, is there evidence that RP-1664 inhibits mouse PLK4, and do the authors have any histopathological data that might clarify the basis of the observed body weight loss in treated mice? Including such information would strengthen the translational relevance of the preclinical findings.

The dosing of PLK4 inhibitors is known to be limited by myelosuppression, which is also likely the reason necessitating intermittent dosing in our current experiments (PMID: 40378279, 38114624). We show in revised **Extended Data Figure 8B,C/Rebuttal Figure 5** that RP-1664 indeed engages mouse PLK4, as witnessed by the protein's stabilization in PLK4-treated Th-MYCN mouse tumors.

3. In Extended Data Fig. 4b, "p21" appears to be misannotated and should be "p53."

The blot indeed shows p21 level in the xenograft model. We used p21 as a functional readout of p53 activity.

4. In Extended Data Fig. 7c, it would ease interpretation if mice requiring treatment interruption due to weight loss were clearly indicated in the figure.

We have revised Extended Fig. 7C accordingly.

5. The boxed label of PLK4 in Fig. 1c is not clearly visible, and it is unclear whether this is intentional, which could lead to misinterpretation.

We thank for pointing this out - the blurring of the PLK4 box was meant to indicate different levels of PLK4 inactivation. We however agree that the design was not very clear and have changed it in the revised figure.

Reviewer #3

(1) 17q as pathognomic biomarker of high-risk neuroblastoma (including ref: https://pubmed.ncbi.nlm.nih.gov/10379019/)

We and others showed decades ago that unbalanced gain of chromosome 17q is a biomarker of high-risk neuroblastoma, but estimates on the exact frequency vary widely in the literature. Here we show that using deep whole genome sequencing methods with careful quality control of tumor cell content that essentially all cases studies have evidence for 17q gain. While nothing is 100% in biology, we make the point that essentially all high-risk neuroblastoma patients will harbor *TRIM37* gain, making it unique amongst human cancers.

(2) Moreover, the mechanistic dissection of the PLK4-TRIM37 has been extensively demonstrated by Meitinger et al. (Nature 2020; TRIM37 controls cancer-specific vulnerability to PLK4 inhibition, PMID: 32908304), including exploration of the therapeutic potential of PLK4 inhibition neuroblastoma CDX models. Moreover, similar mechanistic findings were elaborated on in breast cancer (Yeow et al., Nature, 2020, PMID: 32908313).

We fully agree with the Reviewer that the synthetic lethality paradigm between PLK4 and TRIM37 has been well described in the landmark publications cited above and we do not attempt to claim novelty in that respect. We think that the novelty of our study is primarily two-fold: i) RP-1664 is the first truly selective PLK4 inhibitor that shows oral bioavailability in mammals, which allowed us to extensively validate the tolerability and therapeutic potential of pharmacological PLK4 inhibition in a range of *in vivo* pre-clinical models of neuroblastoma (CDX, PDX, GEMM), well beyond what was present in prior literature (tumor-specific shRNA-mediated downregulation of PLK4 in a single CDX in Meitinger et al., 2020). Moreover, we now added new data showing the combination potential of RP-1664 (see below), further expanding the translational relevance of our study. ii) We demonstrate for the first time that centriole amplification contributes to PLK4i sensitivity in neuroblastoma independently of TRIM37, which is a truly novel finding, and extends the existing PLK4-TRIM37 synthetic lethality framework to a TRIM37-independent mechanism of tumor cell cytotoxicity.

(3) Last, the key mechanistic link with KIF1C was demonstrated for the first time by Zhou et al. in endometrial cancer (KIFC1 depends on TRIM37-mediated ubiquitination of PLK4 to promote centrosome amplification in endometrial cancer; PMID: 39349439).

As in the previous point, we agree that the link between KIFC1 and centrosome clustering is more than well established. In fact, we use genetic inactivation of KIFC1 as a proof-of-principle that blunting a cell's centrosome clustering ability leads to sensitivity to PLK4 inhibition.

It would be very interesting if the authors could further elaborate functionally/mechanistically on the link with the USP28-53BP1 axis as well as the PIDDosome?

As highlighted in our responses to Reviewer #1, point 2 and Reviewer #2, point 2, we were regrettably not successful in evaluating the contribution of PIDD1 to neuroblastoma cell death upon RP-1664 treatment. We aim to address this important question in future studies.

Given the very important and elaborate preclinical work on PLK4 inhibition in this manuscript with various neuroblastoma murine models, I would recommend to transfer this publication eg to Cell Reports Medicine.

We thank the Reviewer for the suggestion but think that Nature Communication is a wonderful home for our manuscript, being in our view an important advance of significance to specialists within our field.

Furthermore, although the data provided in this manuscript show a strong case for single PLK4 inhibition, I think it is always valuable to also scrutinize for potential synergistic drug interactions and would be of great added value to this manuscript.

We thank the Reviewer for raising an important point on synergistic drug combinations. We tested combinations of RP-1664 with two chemo-immunotherapy combination regimens used as standard-of-care in high-risk neuroblastoma: anti-GD2 immunotherapy + irinotecan + temozolomide and anti-GD2 + topotecan + cyclophosphamide. We show in revised **Figure 7C,D** and **Extended Data Figure 8D** a significant combination benefit of RP-1664 + anti-GD2+irinotecan+temozolomide at well-tolerated doses, as compared to both RP-1664 or anti-GD2+irinotecan+temozolomide alone. We suggest that these data substantially strengthen the translational relevance of the manuscript and suggest a path towards clinical testing of standard-of-care regimens combined with PLK4 inhibition. Indeed, this is actively being pursued and a clinical trial based on the data in this manuscript is being prepared with the New Approaches to Neuroblastoma Therapy (NANT; www.nant.org) consortium (Maris PI of NCI P01 that support the NANT).

Reviewer #1

Unfortunately, I do not believe that this manuscript provides substantial novel mechanistic insight. Rather, it largely confirms previously established findings showing that either TRIM37 amplification or activation of the mitotic surveillance pathway (USP28–TP53BP1–p53) can sensitize cancer cells to PLK4 inhibition.

The authors report that CHP134 cells are sensitive to RP-1664–induced centrosome amplification and present this as a novel observation. However, their genetic screen identifies BID, which has already been shown to mediate apoptosis in response to centrosome amplification through a PIDDosome-dependent but p53-independent mechanism (PMID: 39475598). Thus, the primary conclusion of the manuscript appears to be that BID-mediated apoptosis contributes to cell death specifically in CHP134 cells. While this mechanism can explain most of the reported observations, the manuscript does not sufficiently acknowledge or discuss this prior work, and the relevance of BID-mediated apoptosis is not given appropriate attention in the discussion.

In addition, the manuscript contains several incorrect or misleading statements. A few examples are listed below.

1. Misrepresentation of prior work

The abstract states that PLK4 inhibition was recently shown to be synthetic lethal with TRIM37 amplification in a p53-dependent manner. This statement is incorrect. The synthetic lethality between TRIM37 amplification and PLK4 inhibition has been clearly demonstrated to be p53-independent. The abstract therefore presents the prior literature inaccurately and creates the misleading impression that the authors are reporting a new p53-independent mechanism.

We have removed statements about p53-dependency from the abstract, focusing solely on TRIM37, to avoid any potential misrepresentations.

2. Overstated translational claims

Because the manuscript does not establish a novel mechanism, several claims in the abstract appear overstated or insufficiently supported by the data. For example, the authors conclude that “these data support biomarker-directed clinical development of PLK4 inhibitors for high-risk neuroblastoma and other cancers with somatically acquired TRIM37 overexpression.” However, the manuscript simultaneously attempts to argue that sensitivity to PLK4 inhibition is independent of TRIM37 status. Moreover, the proposed link to centrosome clustering defects is not supported by the identification of a clear biomarker. MYCN is mentioned, but no direct mechanistic connection between MYCN expression and PLK4 inhibitor sensitivity is demonstrated. Making biomarker claims without clear supporting evidence is therefore premature.

Here we make the claim in Figure 2 that high-risk neuroblastoma is unique amongst human cancers because of the chromosome arm 17q is seen in essentially all samples sequenced thus making this condition an outstanding candidate for clinical development as screening for 17q gain will not be required. If this drug is to be further developed in other malignancies such as breast cancer, selection of patients with 17q gain will likely be required to maximize potential for patient benefit. We make no claims on *MYCN* amplification status as a biomarker and rather show that PLK4 inhibition can potently regress *MYCN* amplified xenografts as monotherapy and shows curative efficacy in a very aggressive transgenic neuroblastoma model driven by *MYCN* when combined with chemoimmunotherapy. As relapsed *MYCN* amplified disease is typically rapidly

fatal, we highlight the opportunity to develop this novel therapeutic approach in these patients and those whose tumors do not harbor *MYCN* amplification. The trial will include robust correlative immunobiologic endpoints to further understand mechanisms of anti-tumor activity.

3. Insufficient evidence for TRIM37- and p53-independent sensitivity

To convincingly claim that PLK4 inhibitor sensitivity is independent of both TRIM37 and p53 in a clinically relevant context, additional experimental evidence would be required. For example, the authors could demonstrate sensitivity in double knockout models lacking both TRIM37 and p53 (e.g., in the context of Figure 3G), or alternatively test additional CDX or PDX models with normal TRIM37 levels and p53 inactivation.

We think these experiments are beyond the scope of this paper that focuses on providing the preclinical proof-of-concept for clinical development of RP-1664 with chemoimmunotherapy for relapsed high-risk neuroblastoma, with the goal of integrating it into front line therapy where chemoimmunotherapy is now being studied (NCT06172296). Neuroblastoma CDX or PDX models with "normal" TRIM37 levels do not exist, and very few have *TP53* mutations/inactivation. Of note, we have attempted xenograft studies with our double knockout cells but were thwarted by suboptimal growth properties of this cell line *in vivo*. That said, we clearly show *in vitro* that TRIM37-low/TP53-KO cells show similar PLK4i sensitivity as each single knockout (Fig 3E).

Despite these concerns, I believe that the study contains valuable data and could make a useful contribution if the manuscript were reframed. Rather than presenting the findings as a fundamentally new mechanism, the authors could position the work as a systematic evaluation of multiple pathways that contribute to sensitivity to PLK4 inhibition.

A possible restructuring could include the following points:

1. Use the data currently shown in Figures 1 and 4 to confirm previously described mechanisms through which PLK4 inhibition can induce cell death, including

i)TRIM37 amplification

ii) Mitotic surveillance (USP28–TP53BP1–p53 pathway)

iii) PIDDosome activation in either a p53-dependent manner (via MDM2 cleavage) or a p53-independent manner (via BID cleavage).

2. Present the data in Figure 3 as preliminary evidence that multipolar mitosis and defects in centrosome clustering may additionally contribute to sensitivity.

3. Highlight the analysis of TRIM37 amplification in high-risk neuroblastoma (currently Figure 2) to emphasize the value of TRIM37 as a biomarker.

4. Present the xenograft experiments in TRIM37-amplified neuroblastoma models (currently Figure 6).

5. Finally, include the data suggesting that PLK4 inhibition may also have efficacy beyond TRIM37-amplified tumors (currently Figure 7).

Reframing the manuscript in this manner would provide a clearer and more balanced presentation of the data while aligning the conclusions more closely with the existing literature.

Based on our responses above and below, we respectfully prefer the current structure of our manuscript that we think proves the point that we have developed a data package that clearly justifies the clinical development of RP-1664 with chemoimmunotherapy in high-risk neuroblastoma.

Reviewer #2 (Remarks to the Author):

The authors have made substantial efforts to address the reviewers' concerns, noting that some limitations were unavoidable due to the recent lab closure at Repare Therapeutics. I have one remaining concern that should be resolved prior to publication.

In response to my question regarding tolerability, the authors state: "the dosing of PLK4 inhibitors is known to be limited by myelosuppression, which is also likely the reason necessitating intermittent dosing in our current experiments (PMID: 40378279, 38114624)." However, one of the cited references characterizes CFI-400945, a molecule widely recognized to have significant Aurora B inhibitory activity; thus, any observed immunosuppression phenotype in that context is likely attributable to Aurora B inhibition rather than selective PLK4 targeting. The second cited study, from the Repare team, does not report myelosuppression, although it does note that continuous dosing with RP-1664 at 600 ppm was not tolerated.

Given this, I strongly recommend that the authors include data demonstrating the effects of RP-1664 on peripheral blood counts in mice. Such data would be highly informative and would provide important support for the anticipated on-mechanism hematologic toxicities associated with PLK4 inhibition in the clinical setting.

While we regrettably no longer have samples available from our mouse studies to analyze complete blood counts, nor do we have the RP-16664 mouse chow to repeat these experiments, we would like to point out that as of late 2025, initial safety results from the LIONS clinical trial evaluating RP-1664 in advanced solid tumors have been posted on clinicaltrials.gov (https://clinicaltrials.gov/study/NCT06232408?tab=results). These data show emergence of manageable hematological toxicities at higher doses with only one hematologic serious adverse event.

Reviewer #3 (Remarks to the Author): no further comments, acceptance for publication
